# Supplementary figures and images for: MiR-31 improves spinal cord injury in mice by promoting the migration of bone marrow mesenchymal stem cells
Source: PLoS One. 2022 Sep 6;17(9):e0272499. doi: 10.1371/journal.pone.0272499 (PMC9447891; doi:10.1371/journal.pone.0272499)

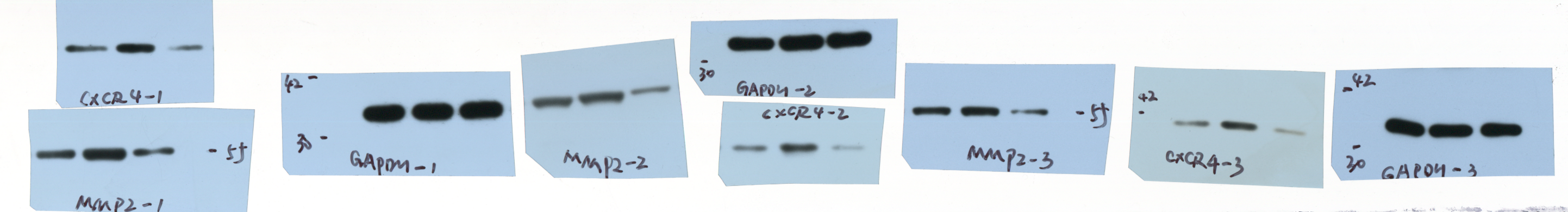

Supplement: S1 File — (ZIP) [file pone.0272499.s001.zip › supporting information/WB/animal-WB.tif]

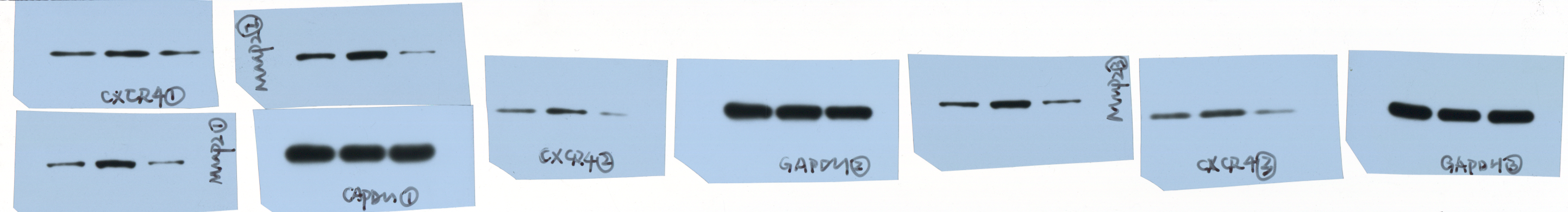

Supplement: S1 File — (ZIP) [file pone.0272499.s001.zip › supporting information/WB/cell-WB.tif]

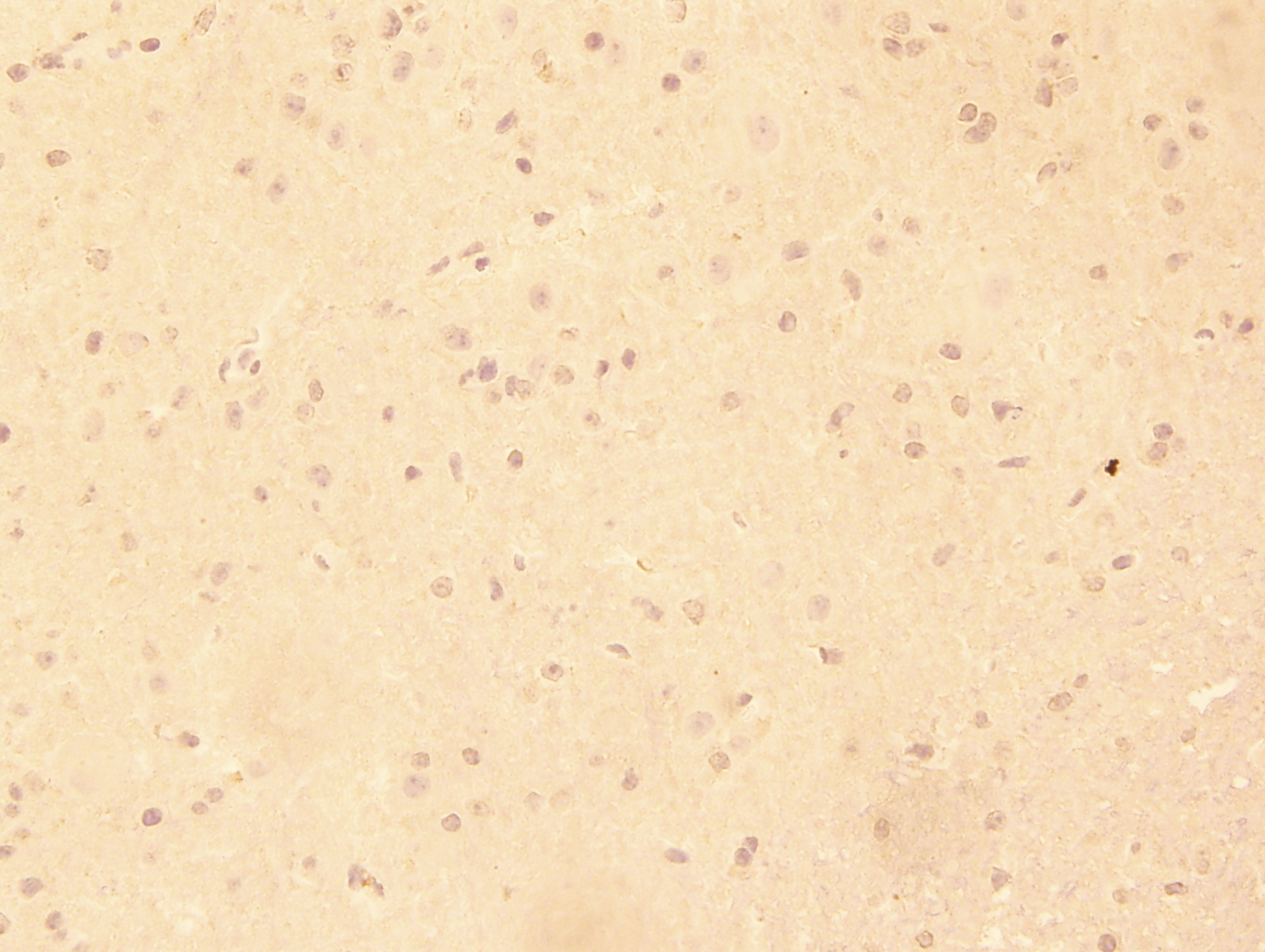

Supplement: S1 File — (ZIP) [file pone.0272499.s001.zip › supporting information/immunohistochemical/controlCXCR4/5days/1.jpg]

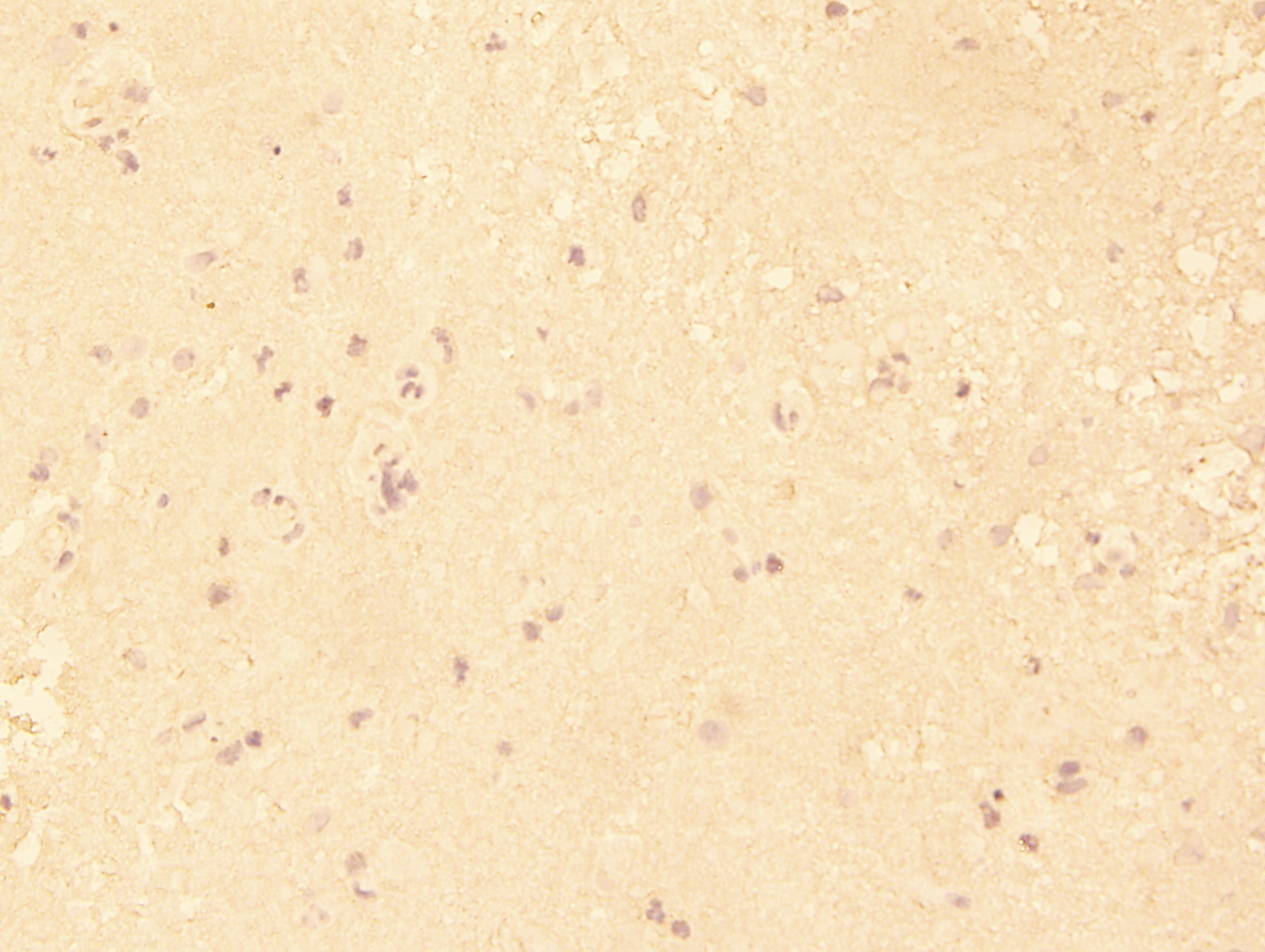

Supplement: S1 File — (ZIP) [file pone.0272499.s001.zip › supporting information/immunohistochemical/controlCXCR4/5days/2.jpg]

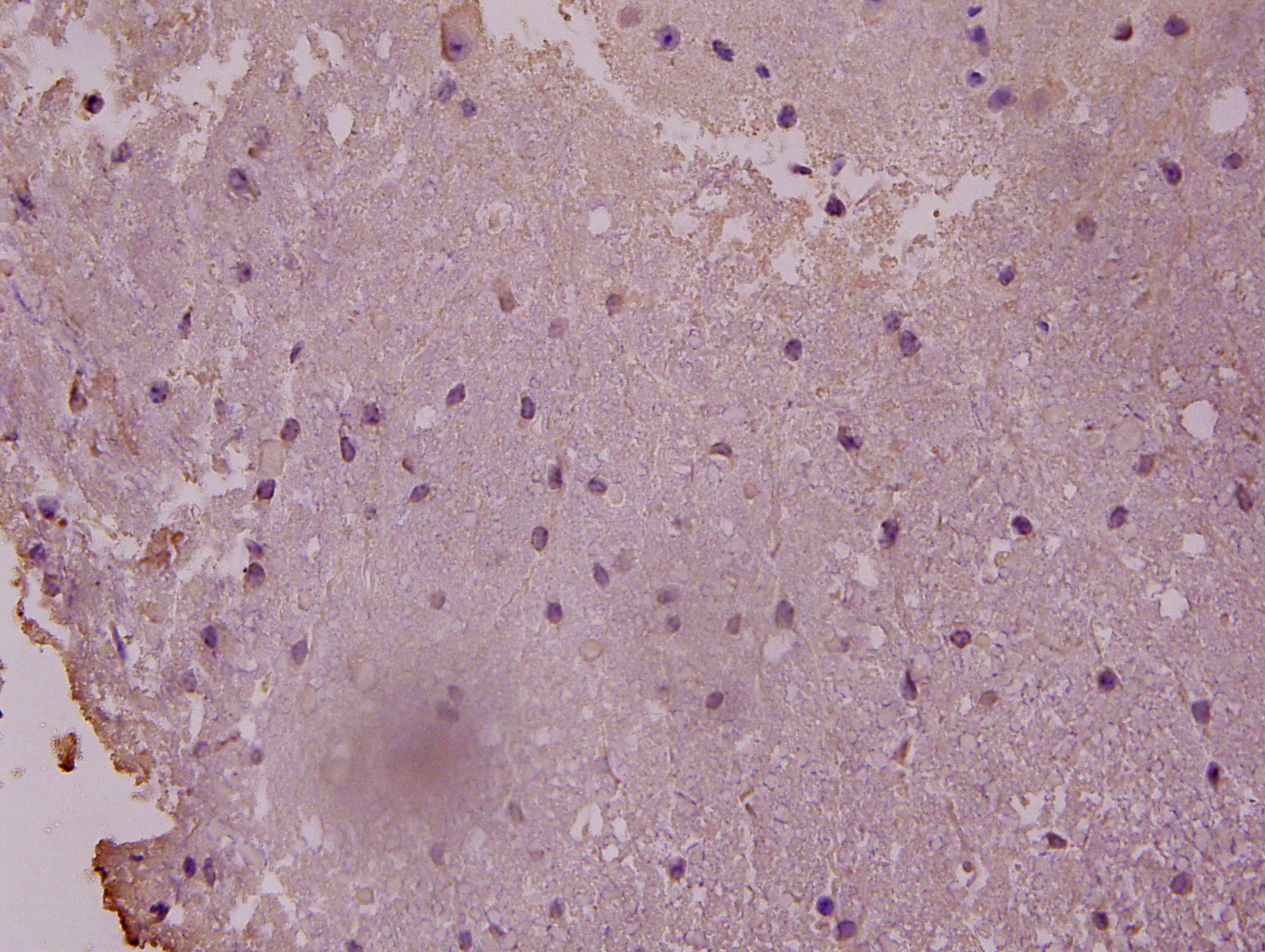

Supplement: S1 File — (ZIP) [file pone.0272499.s001.zip › supporting information/immunohistochemical/controlCXCR4/5days/3.jpg]

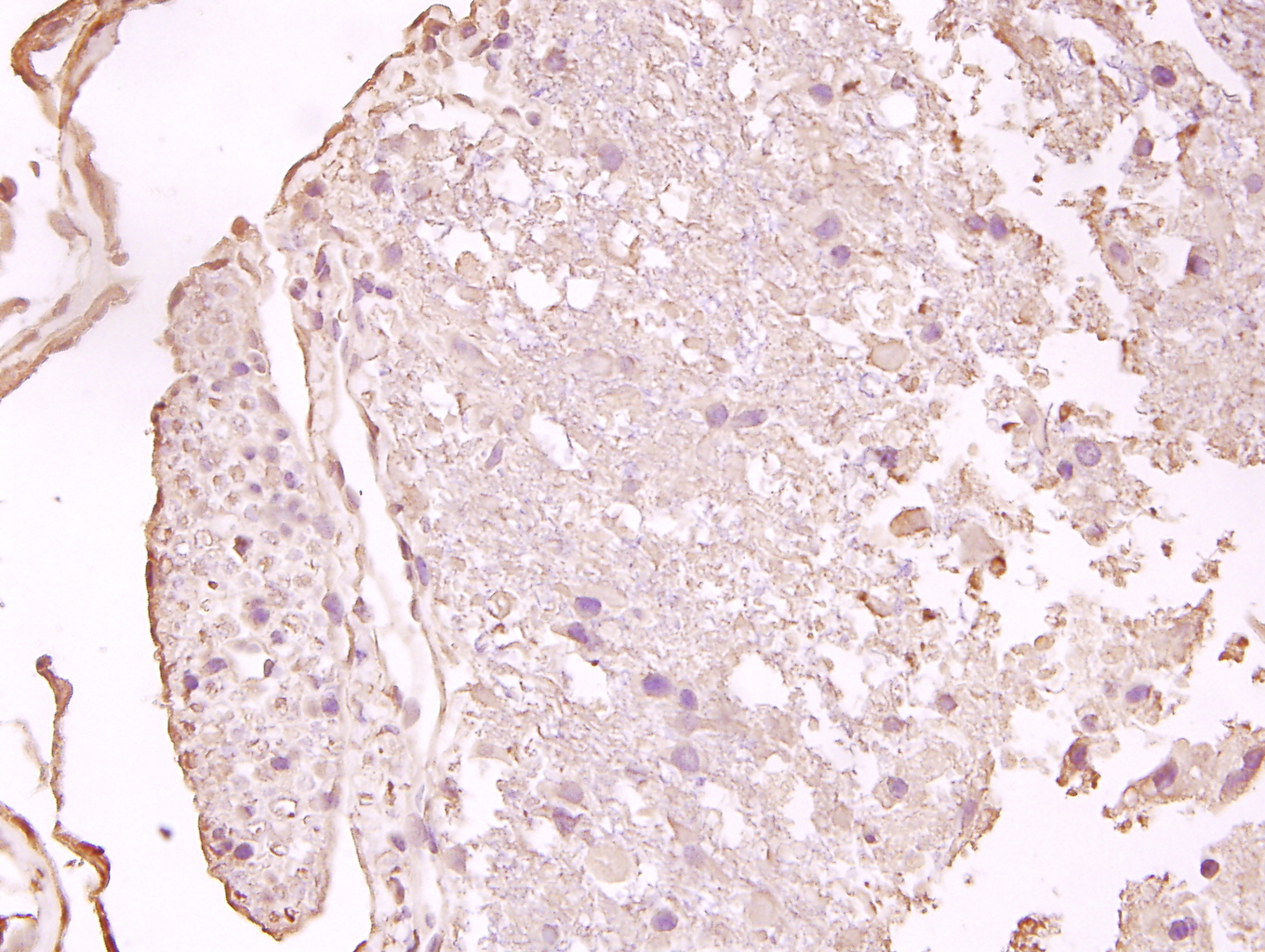

Supplement: S1 File — (ZIP) [file pone.0272499.s001.zip › supporting information/immunohistochemical/controlCXCR4/5days/4.jpg]

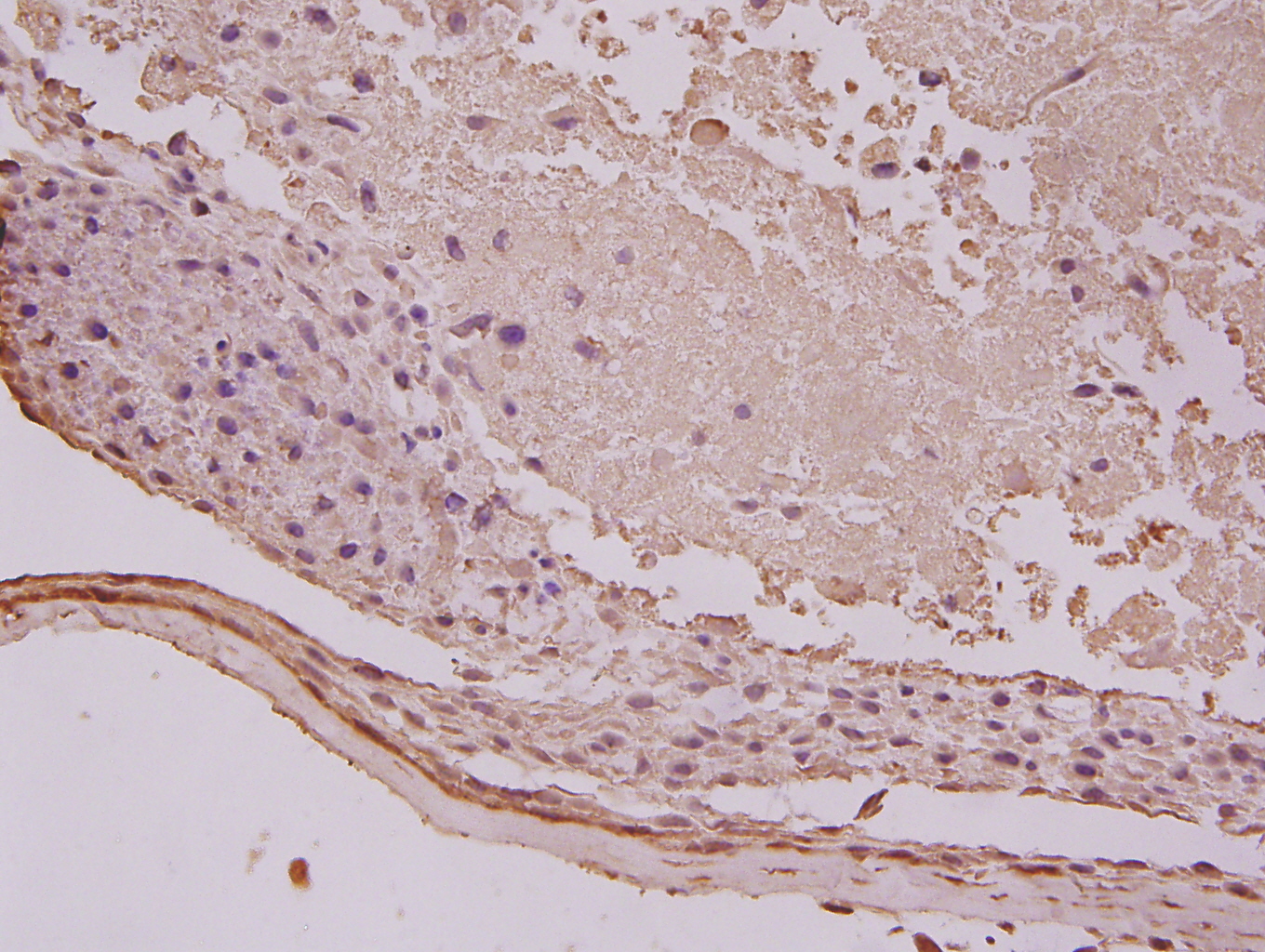

Supplement: S1 File — (ZIP) [file pone.0272499.s001.zip › supporting information/immunohistochemical/controlCXCR4/5days/5.jpg]

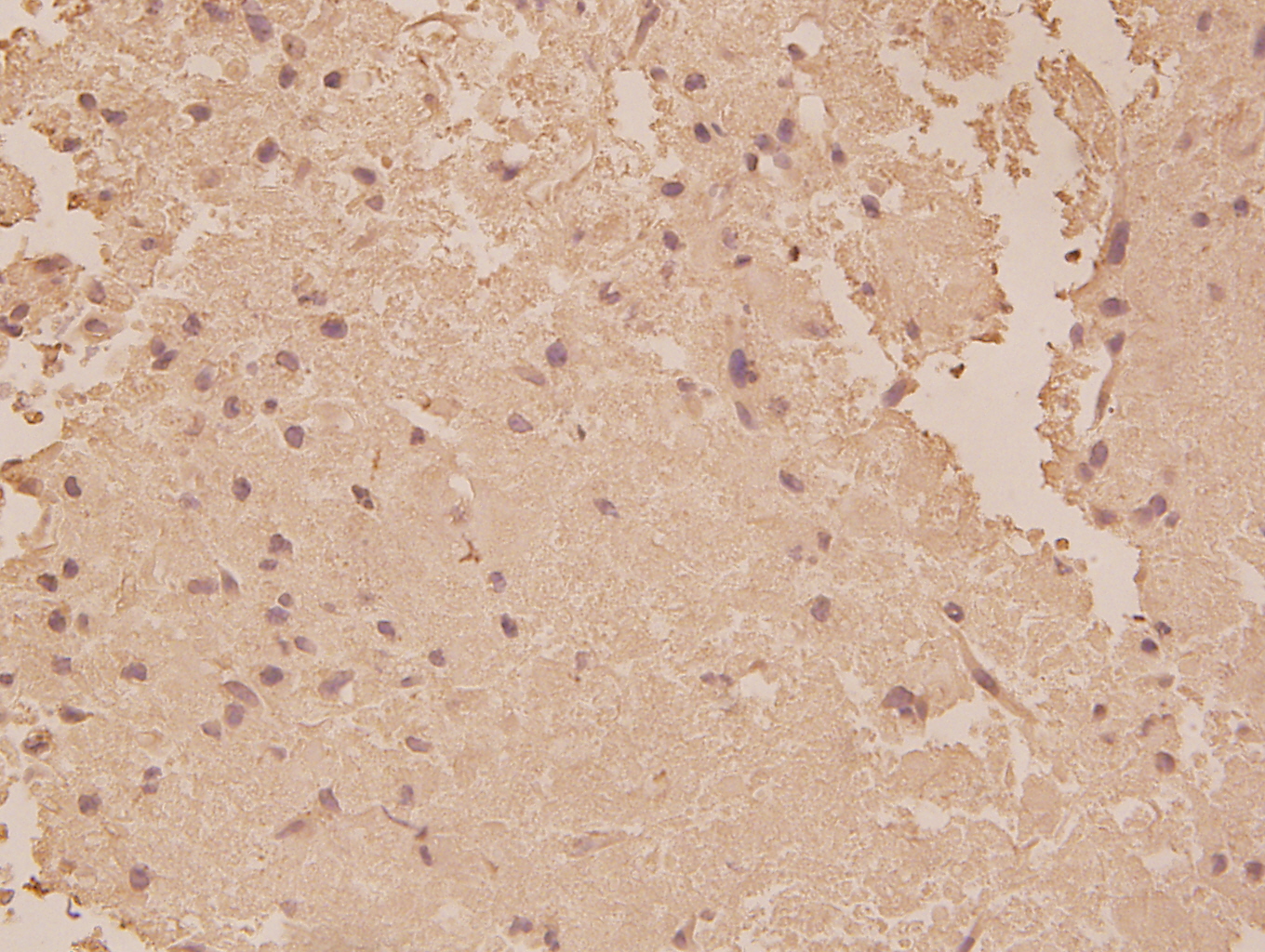

Supplement: S1 File — (ZIP) [file pone.0272499.s001.zip › supporting information/immunohistochemical/controlCXCR4/7days/1.jpg]

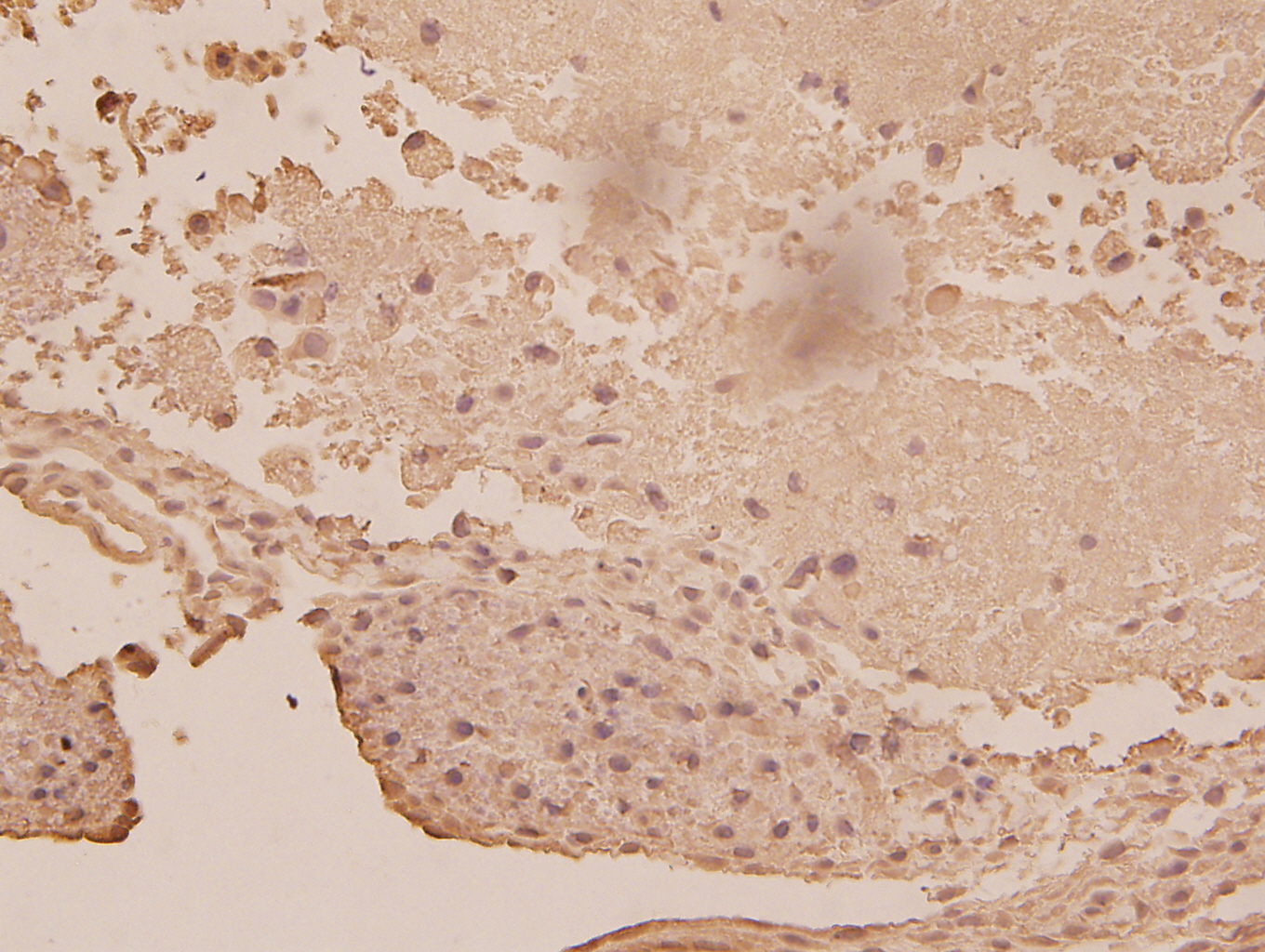

Supplement: S1 File — (ZIP) [file pone.0272499.s001.zip › supporting information/immunohistochemical/controlCXCR4/7days/2.jpg]

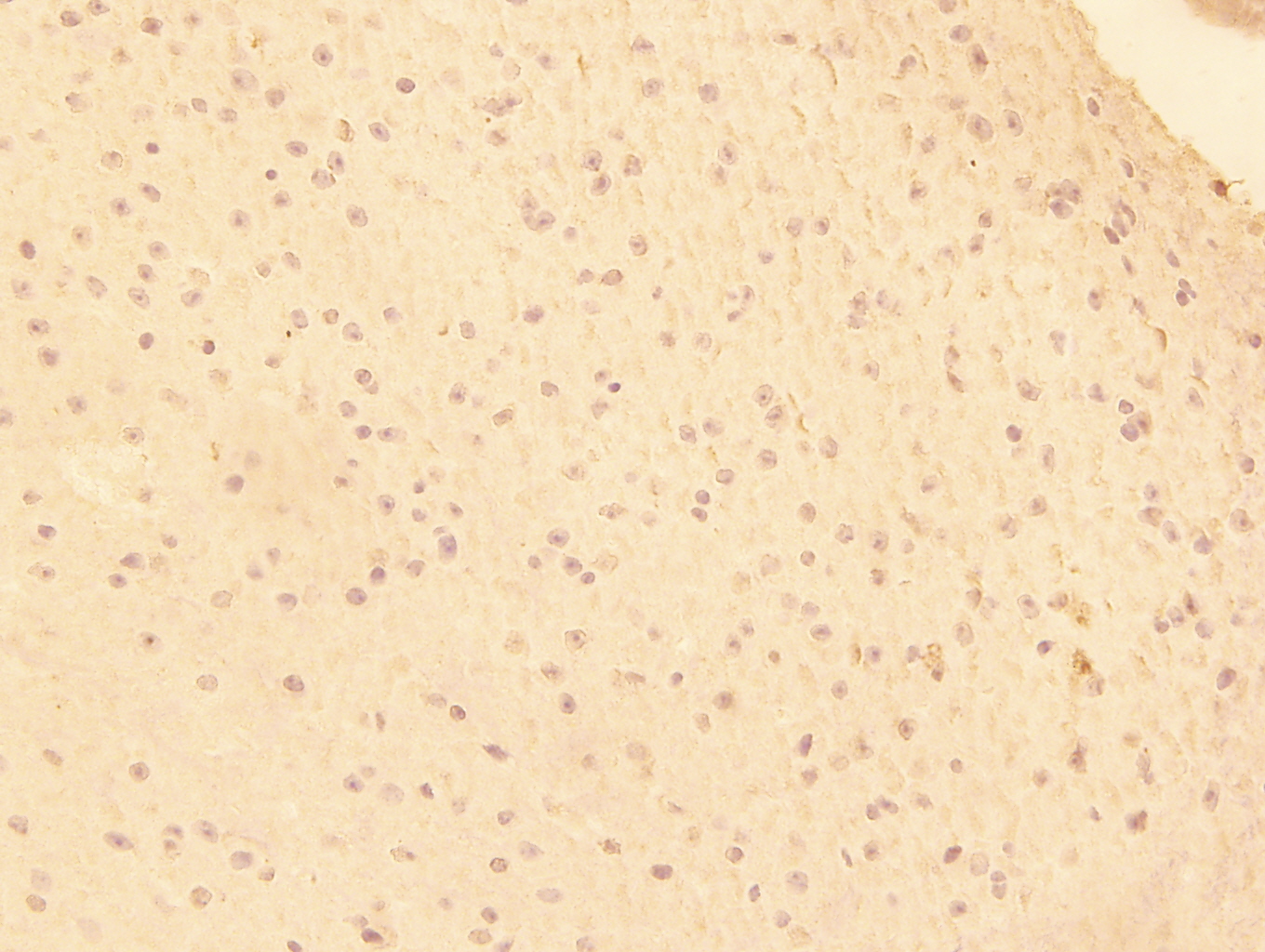

Supplement: S1 File — (ZIP) [file pone.0272499.s001.zip › supporting information/immunohistochemical/controlCXCR4/7days/3.jpg]

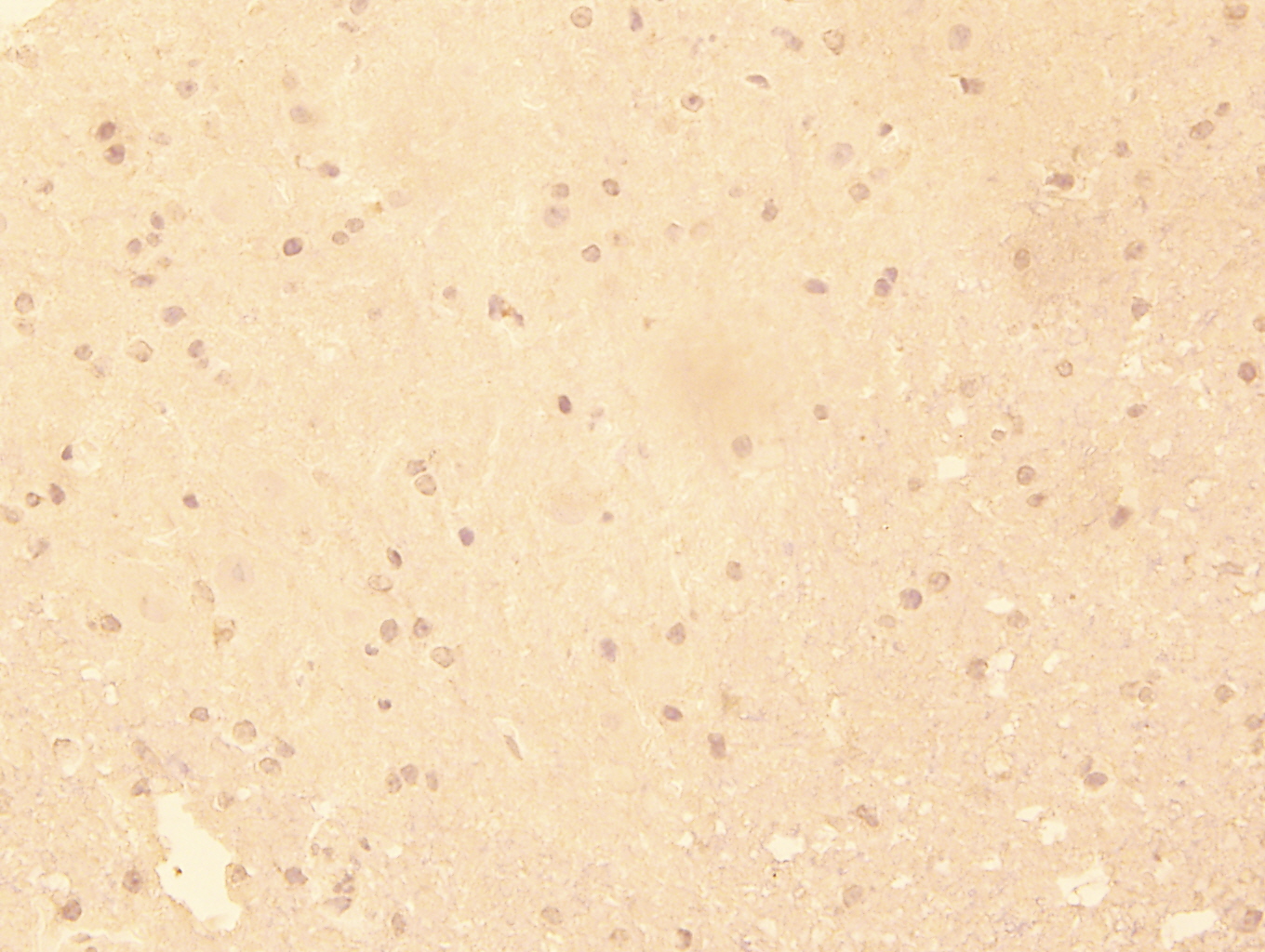

Supplement: S1 File — (ZIP) [file pone.0272499.s001.zip › supporting information/immunohistochemical/controlCXCR4/7days/4.jpg]

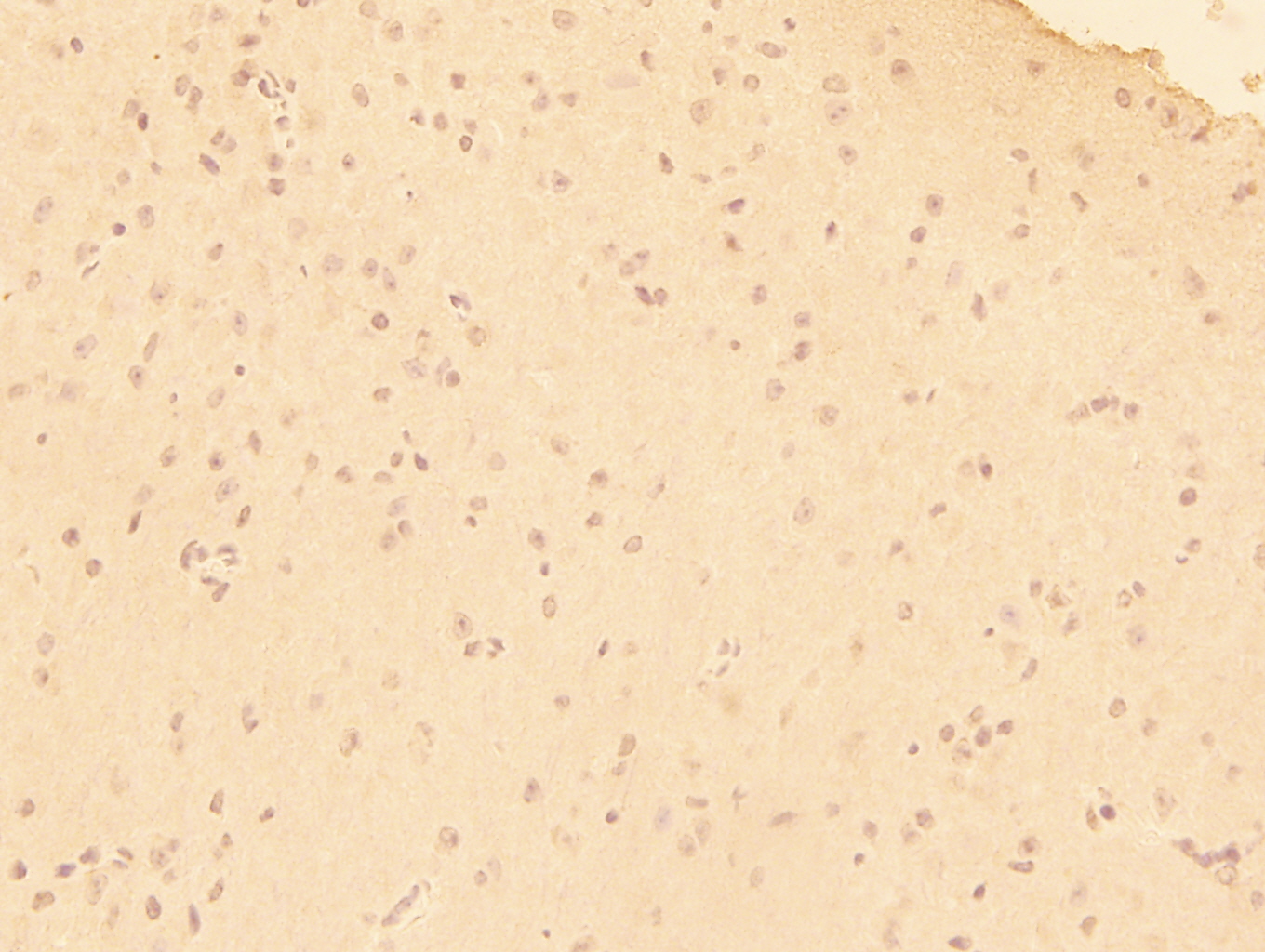

Supplement: S1 File — (ZIP) [file pone.0272499.s001.zip › supporting information/immunohistochemical/controlCXCR4/7days/5.jpg]

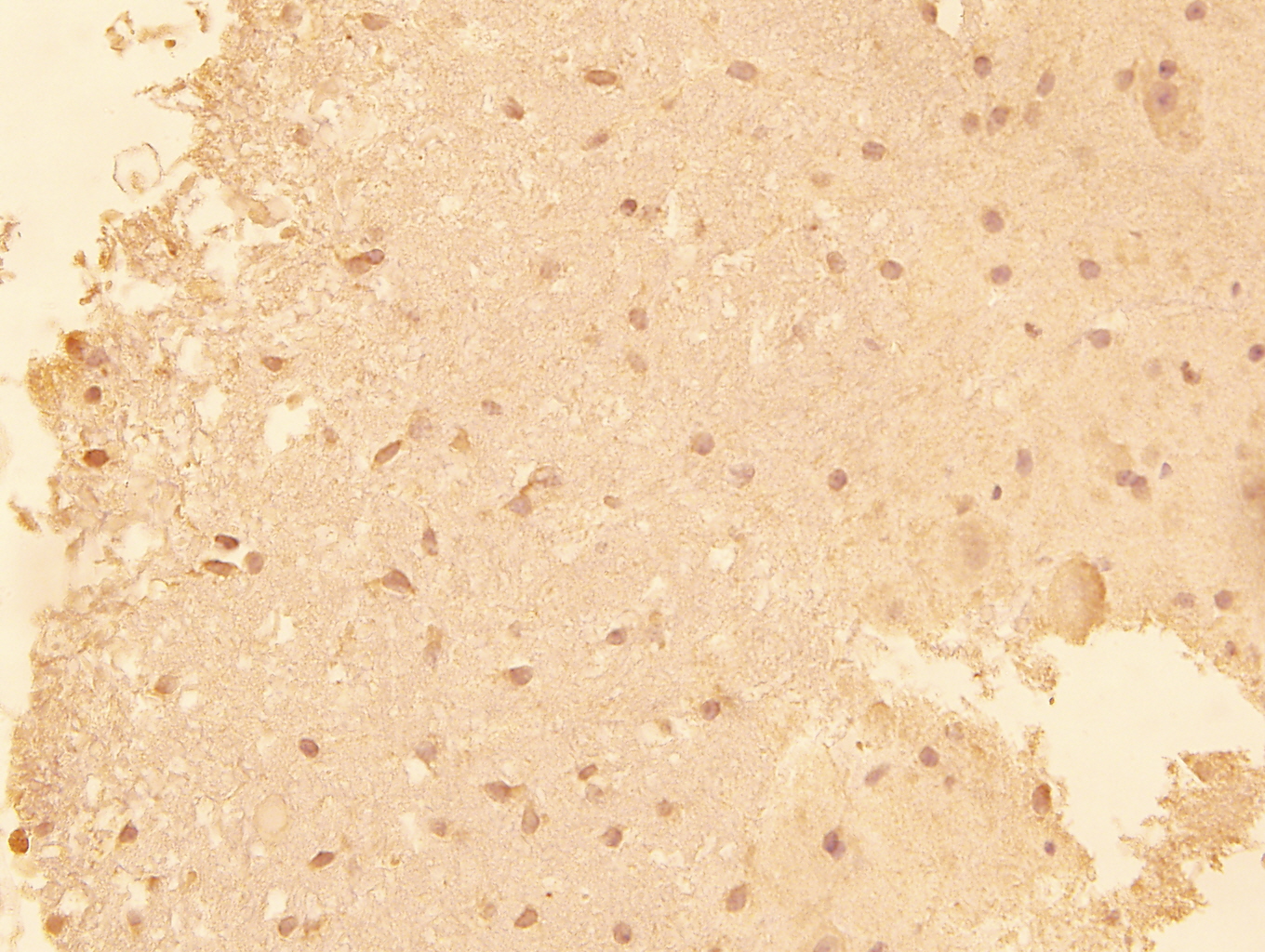

Supplement: S1 File — (ZIP) [file pone.0272499.s001.zip › supporting information/immunohistochemical/controlMMP2/5days/1.jpg]

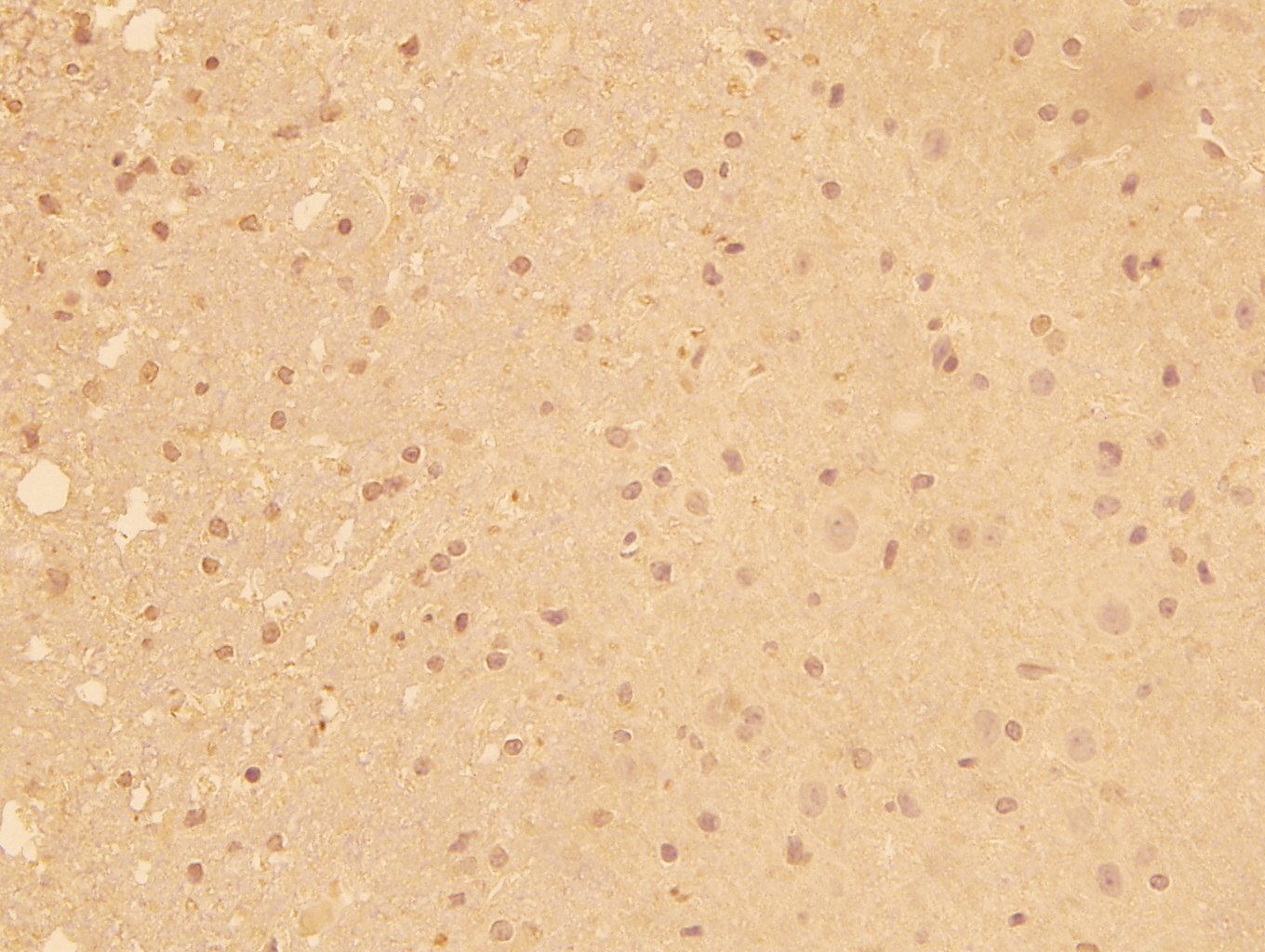

Supplement: S1 File — (ZIP) [file pone.0272499.s001.zip › supporting information/immunohistochemical/controlMMP2/5days/2.jpg]

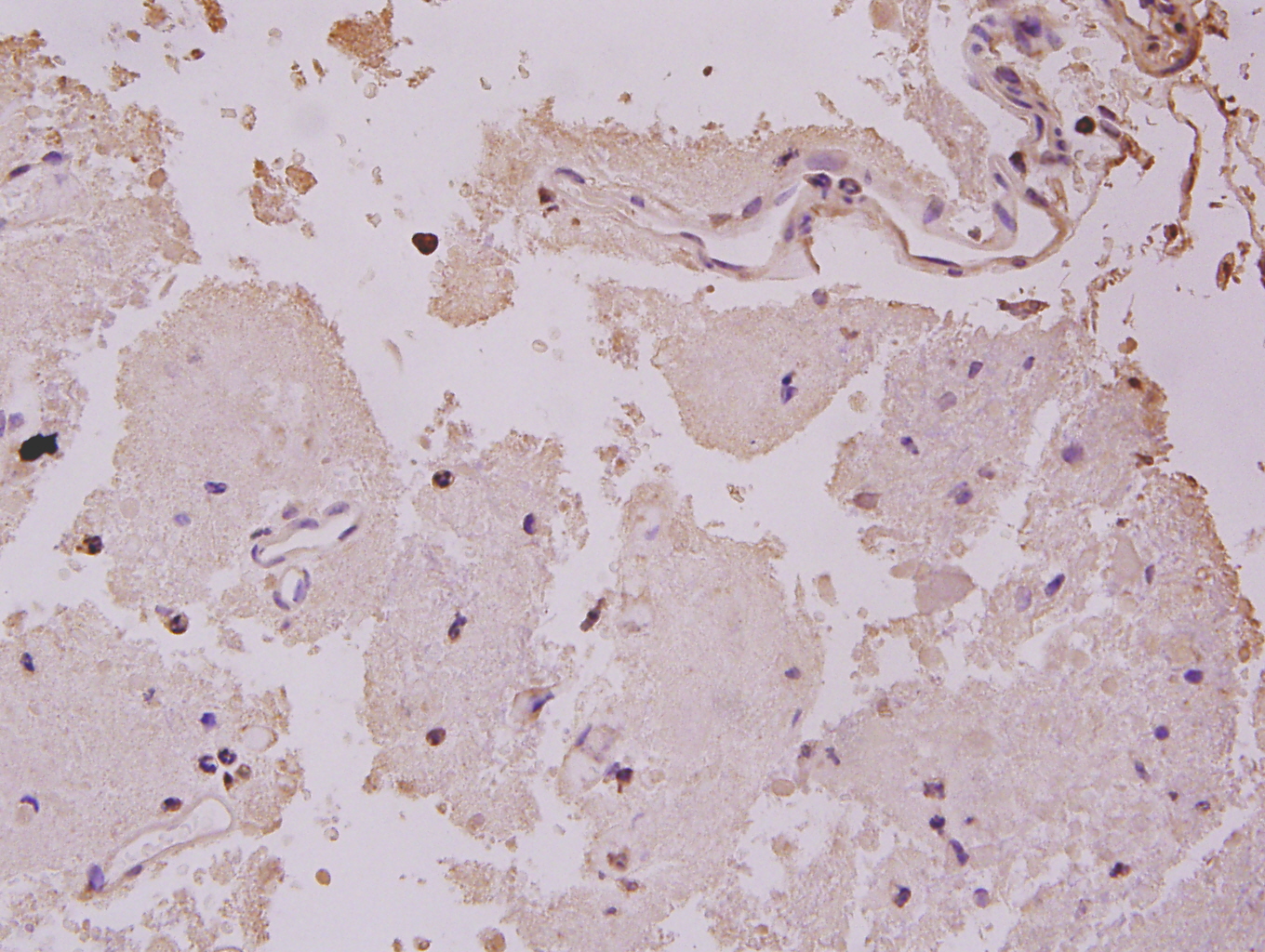

Supplement: S1 File — (ZIP) [file pone.0272499.s001.zip › supporting information/immunohistochemical/controlMMP2/5days/3.jpg]

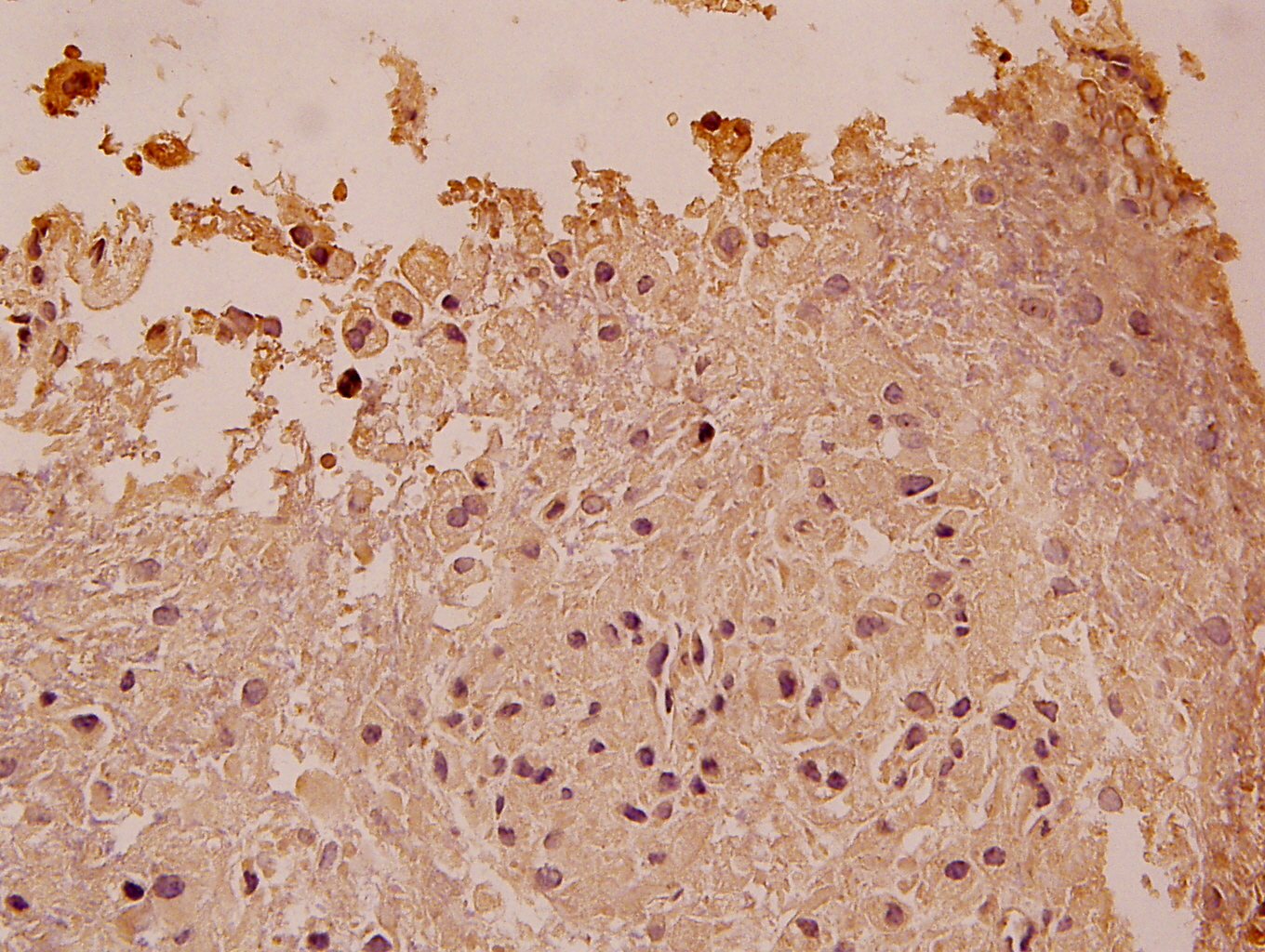

Supplement: S1 File — (ZIP) [file pone.0272499.s001.zip › supporting information/immunohistochemical/controlMMP2/5days/4.jpg]

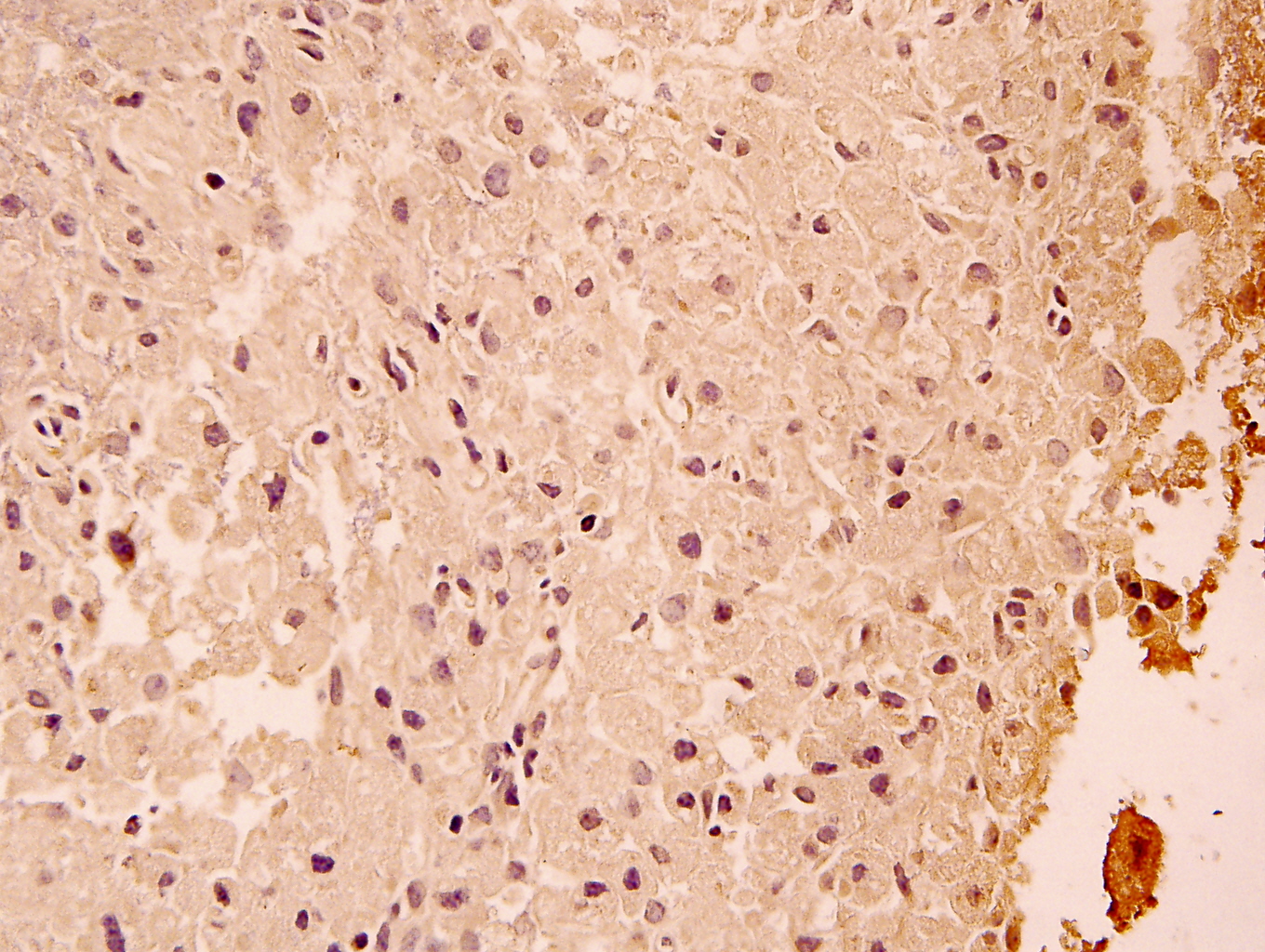

Supplement: S1 File — (ZIP) [file pone.0272499.s001.zip › supporting information/immunohistochemical/controlMMP2/5days/5.jpg]

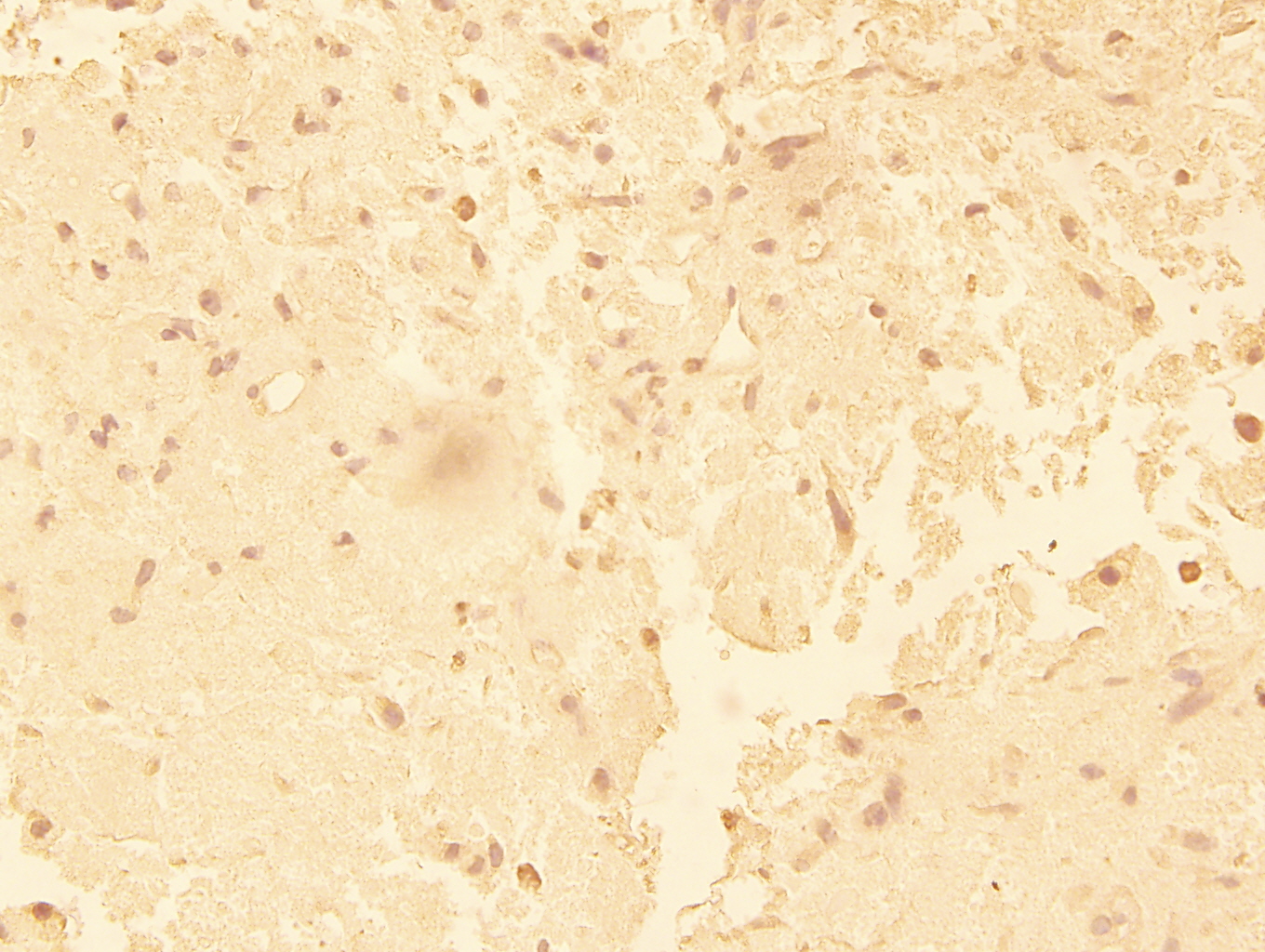

Supplement: S1 File — (ZIP) [file pone.0272499.s001.zip › supporting information/immunohistochemical/controlMMP2/7days/1.jpg]

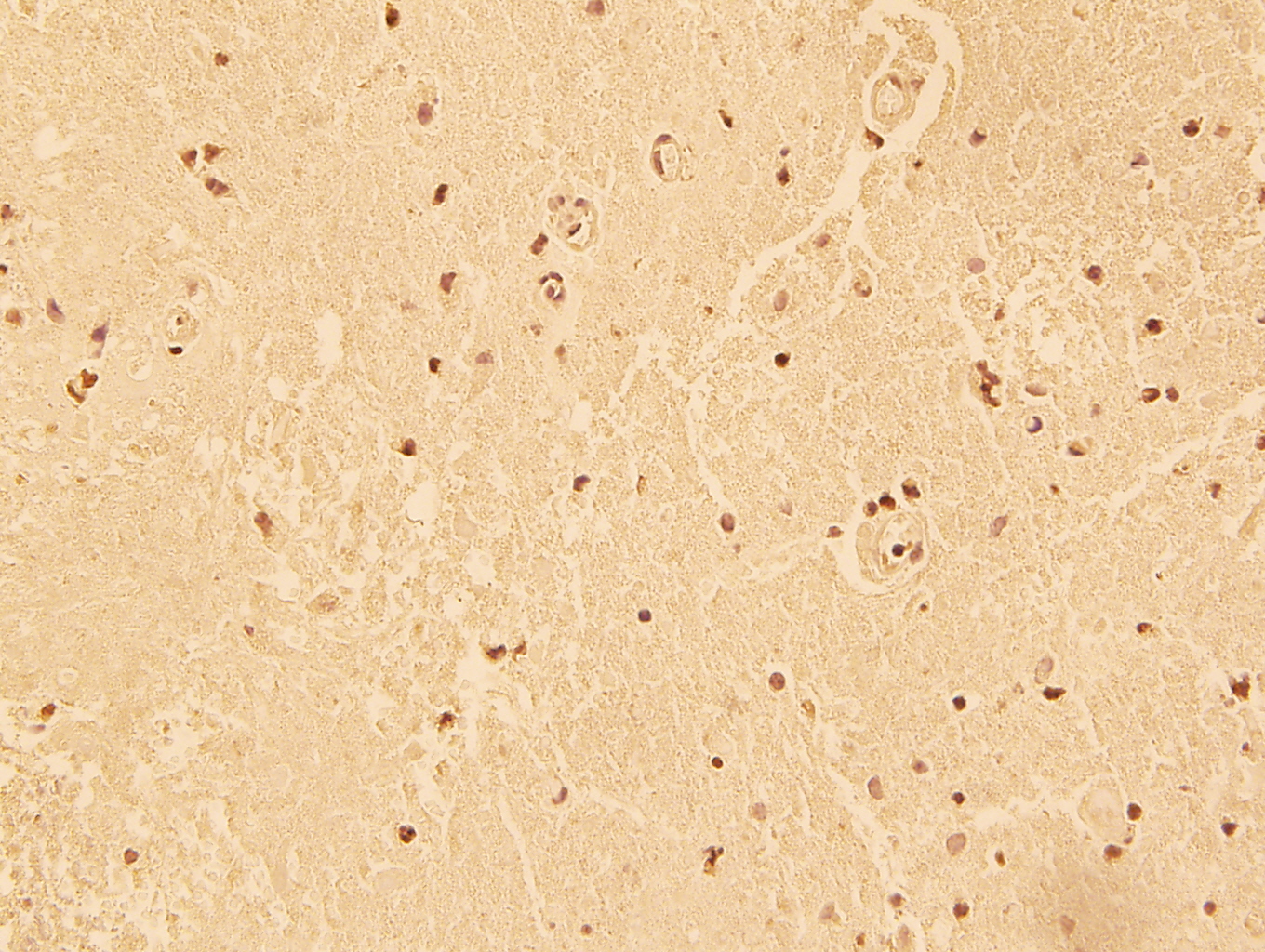

Supplement: S1 File — (ZIP) [file pone.0272499.s001.zip › supporting information/immunohistochemical/controlMMP2/7days/2.jpg]

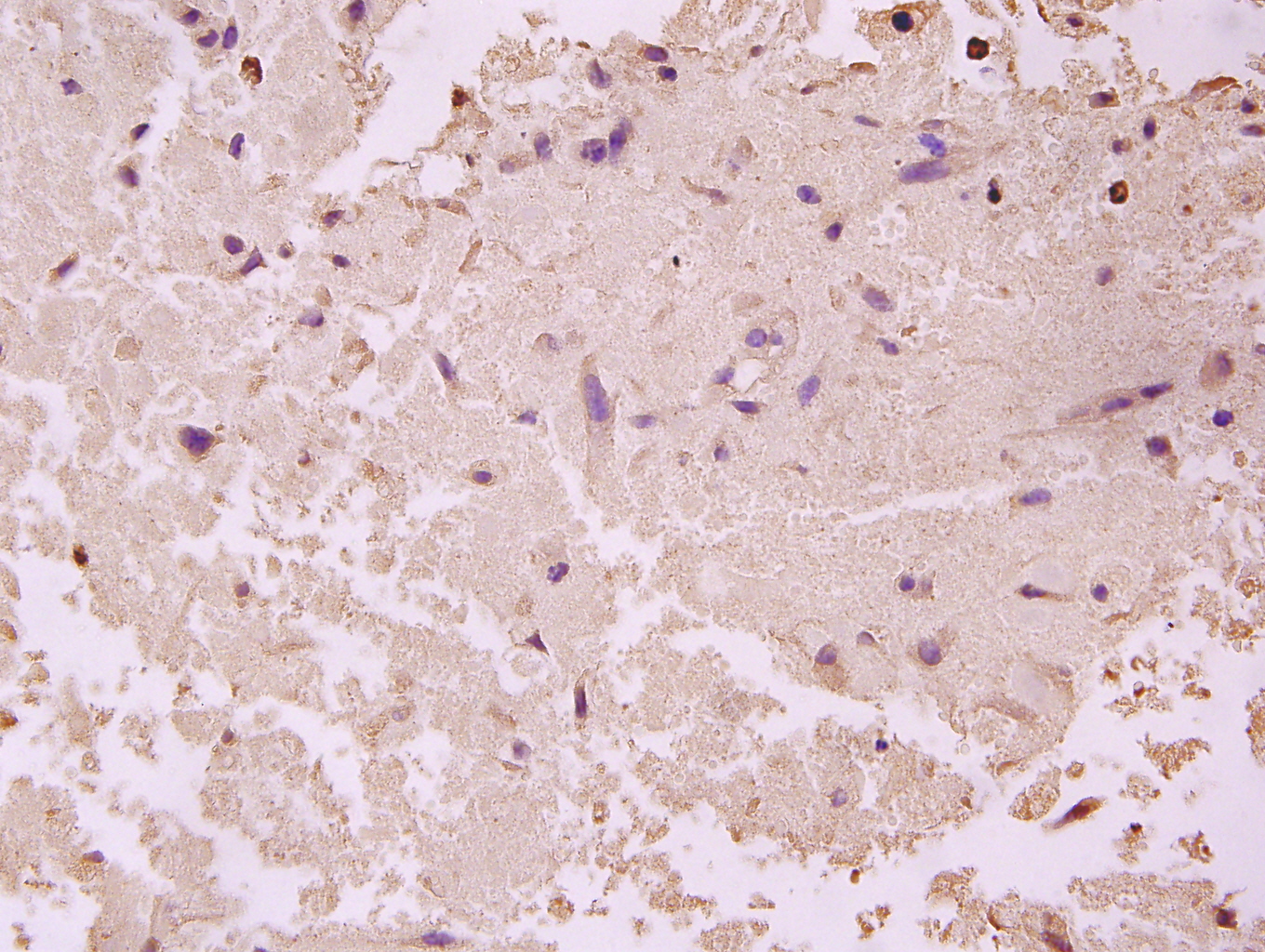

Supplement: S1 File — (ZIP) [file pone.0272499.s001.zip › supporting information/immunohistochemical/controlMMP2/7days/3.jpg]

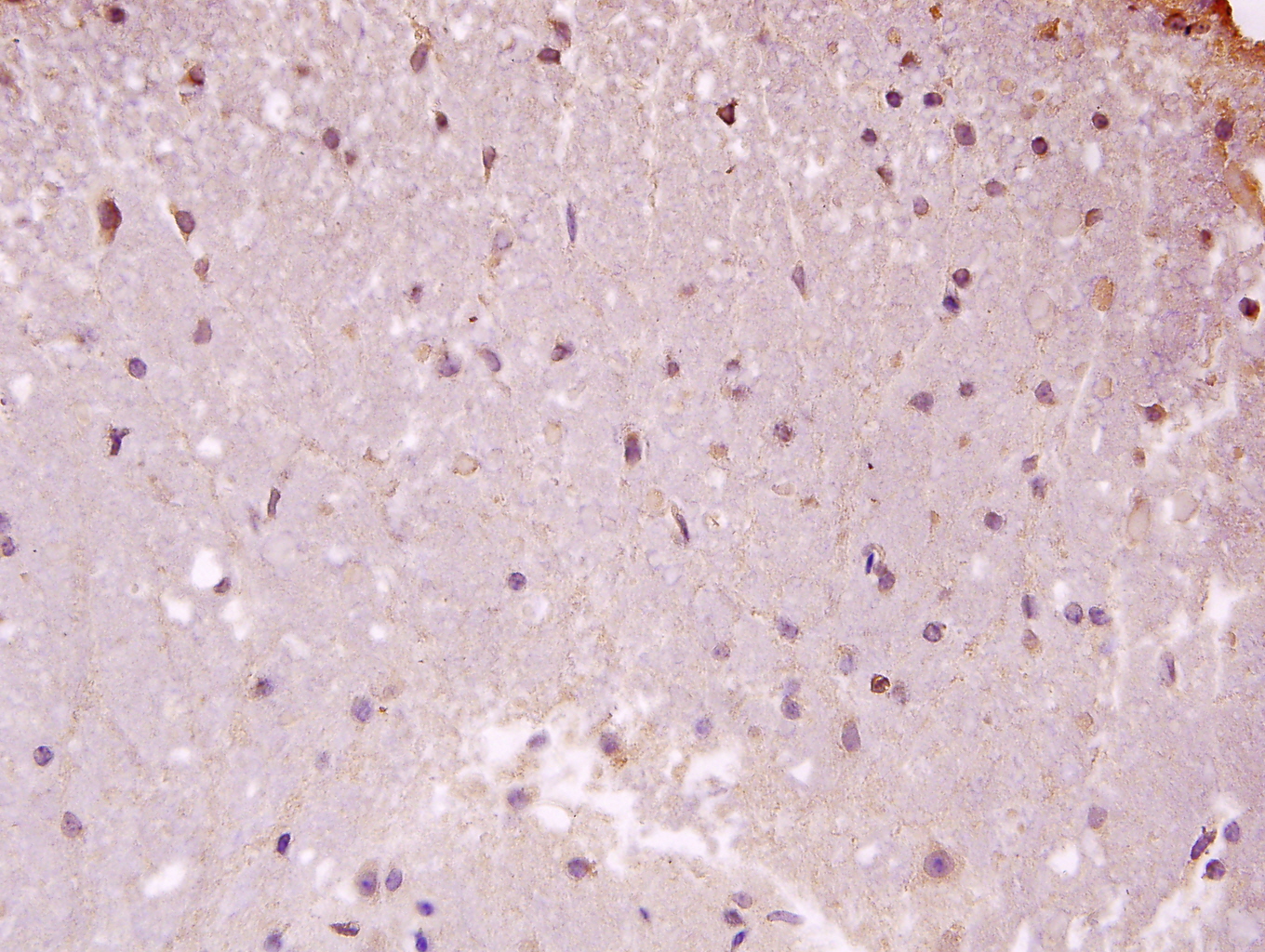

Supplement: S1 File — (ZIP) [file pone.0272499.s001.zip › supporting information/immunohistochemical/controlMMP2/7days/4.jpg]

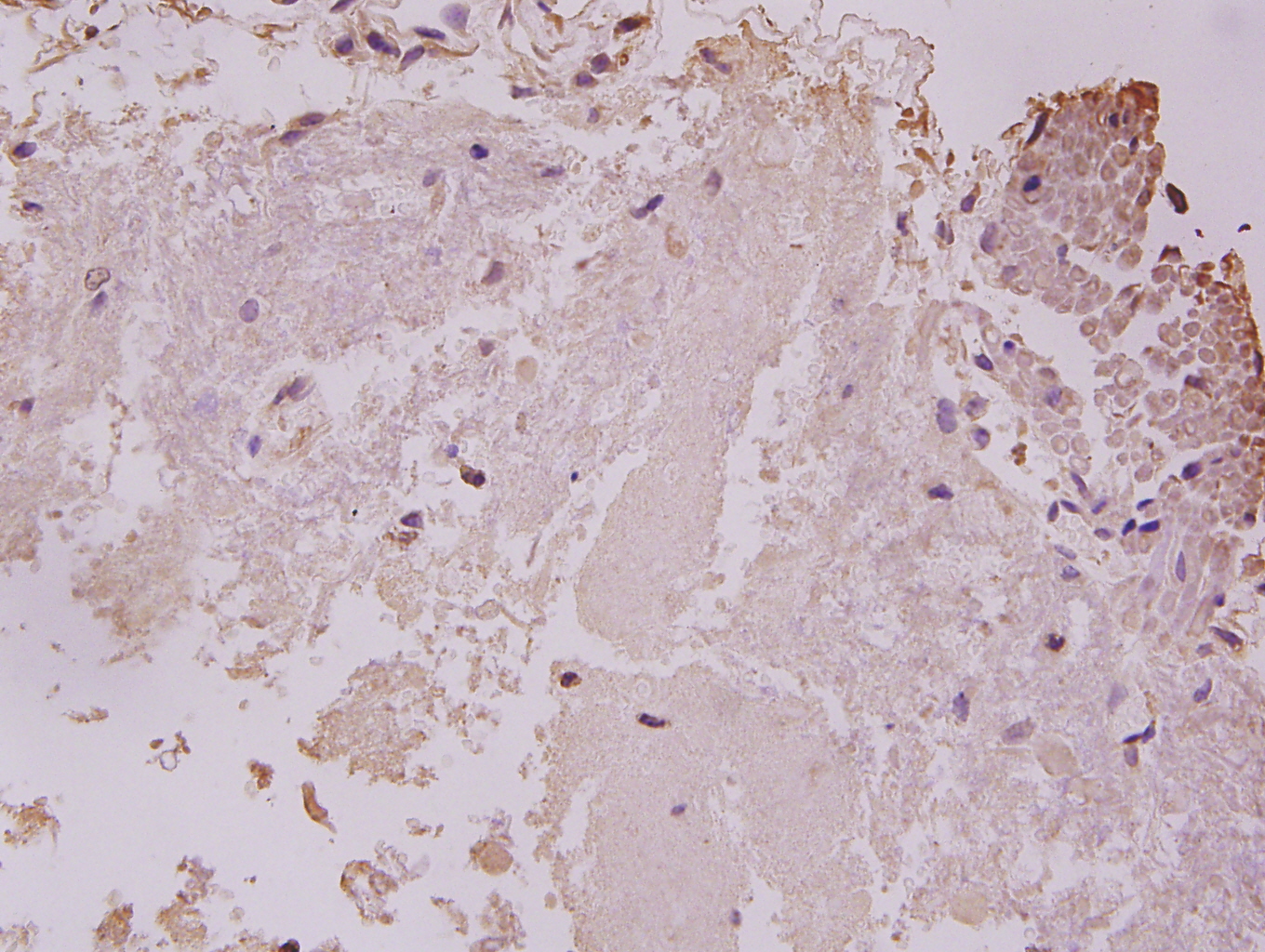

Supplement: S1 File — (ZIP) [file pone.0272499.s001.zip › supporting information/immunohistochemical/controlMMP2/7days/5.jpg]

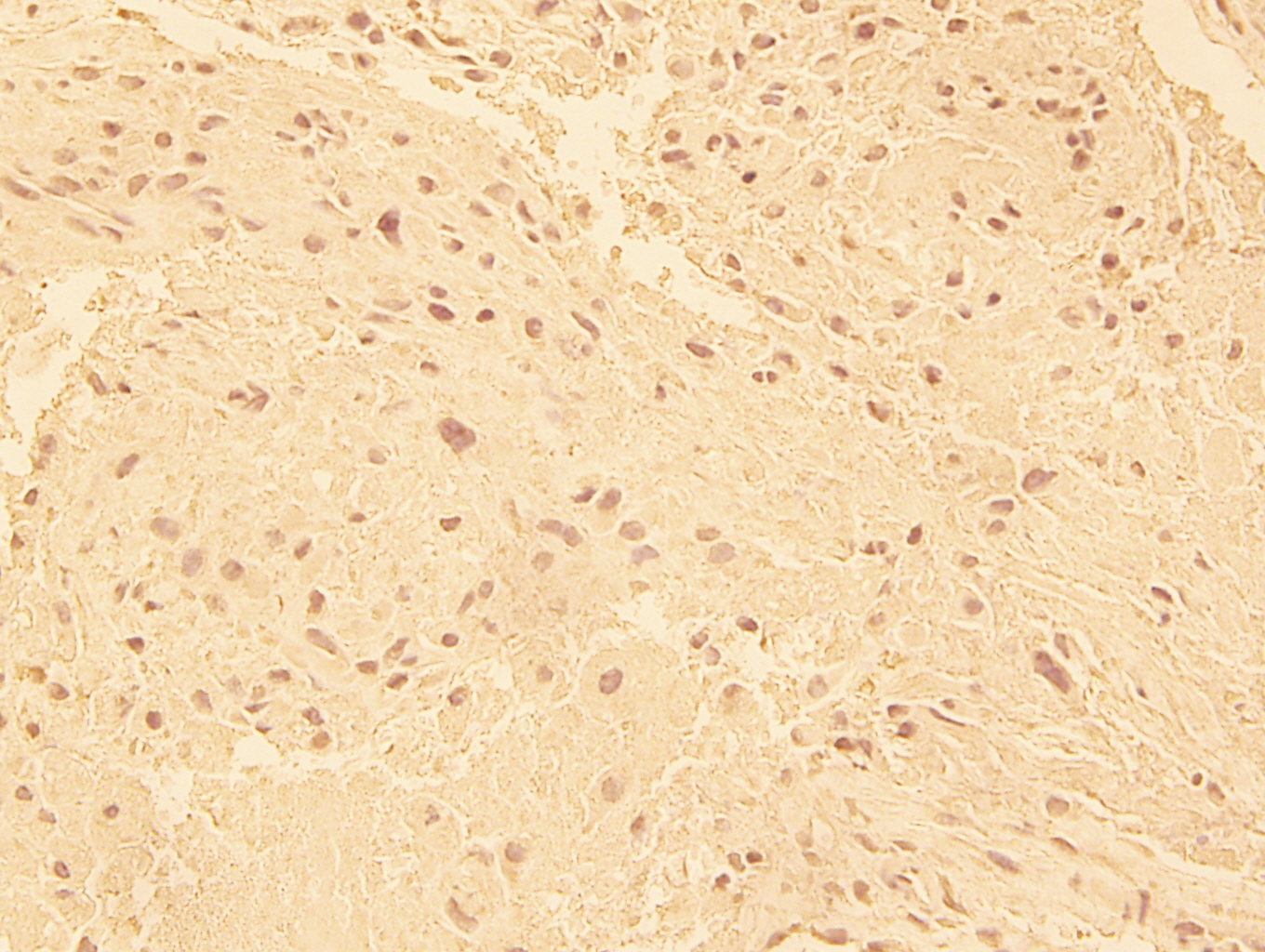

Supplement: S1 File — (ZIP) [file pone.0272499.s001.zip › supporting information/immunohistochemical/miR-31agomir CXCR4/5days/1.jpg]

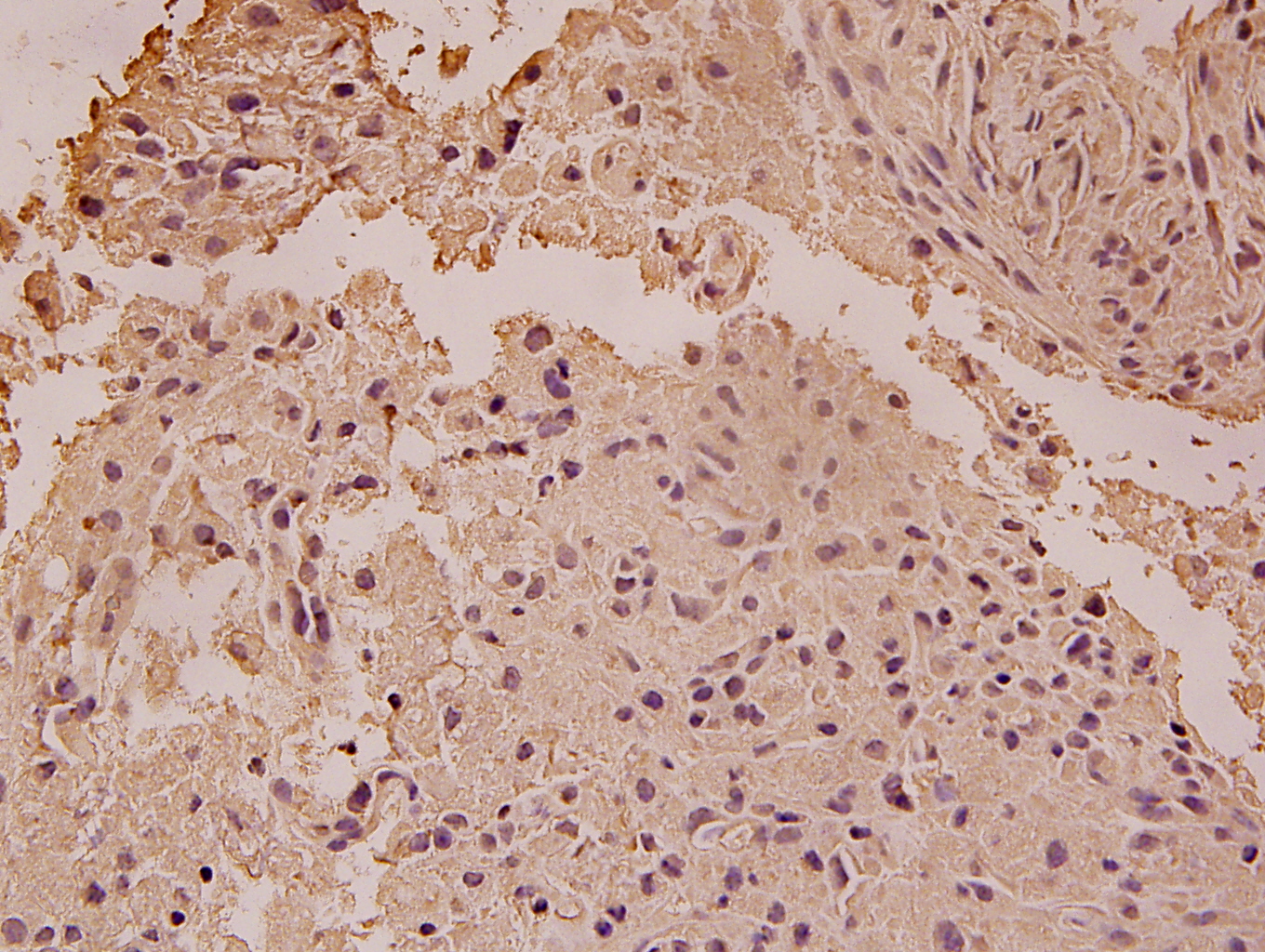

Supplement: S1 File — (ZIP) [file pone.0272499.s001.zip › supporting information/immunohistochemical/miR-31agomir CXCR4/5days/2.jpg]

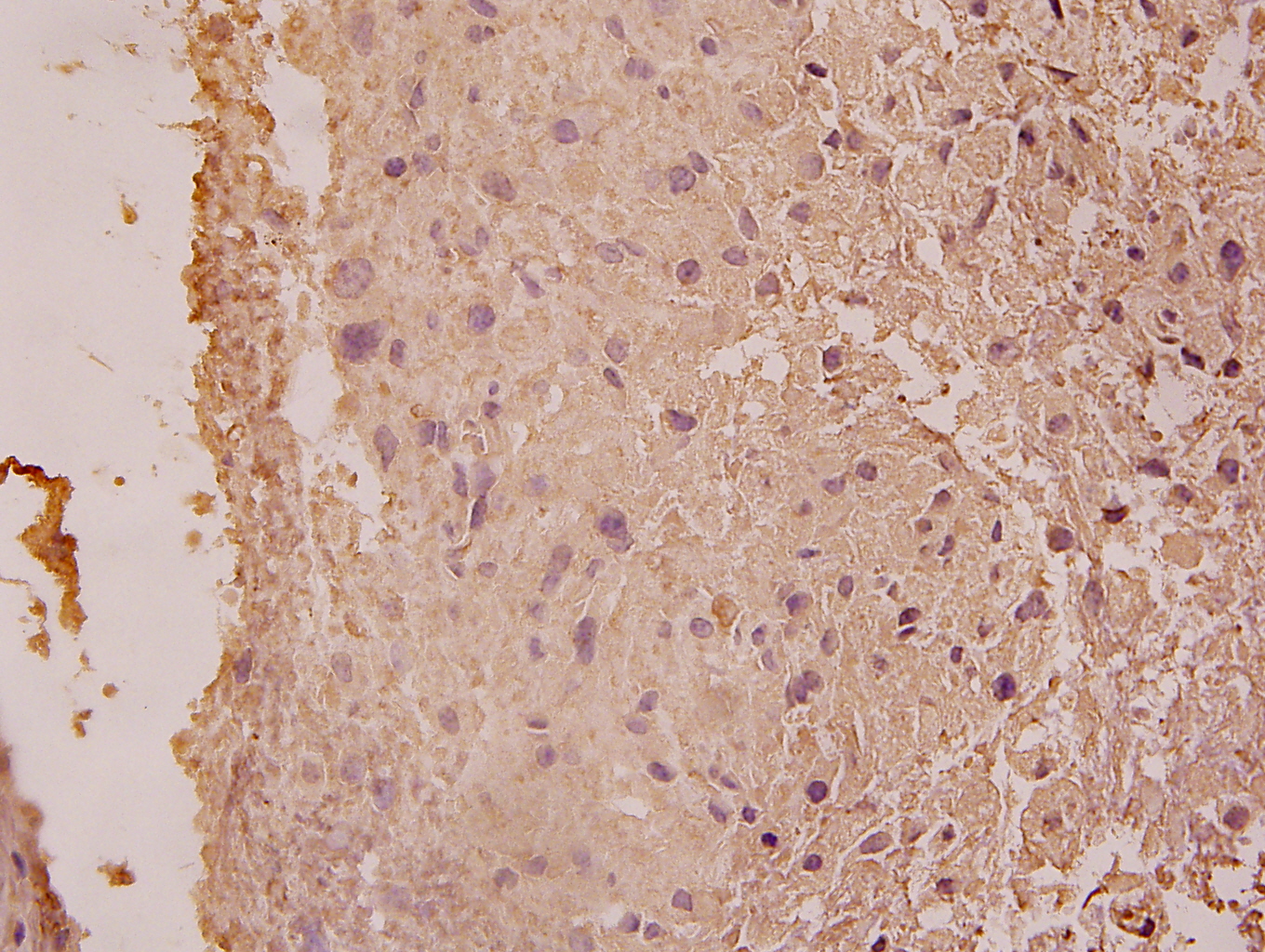

Supplement: S1 File — (ZIP) [file pone.0272499.s001.zip › supporting information/immunohistochemical/miR-31agomir CXCR4/5days/3.jpg]

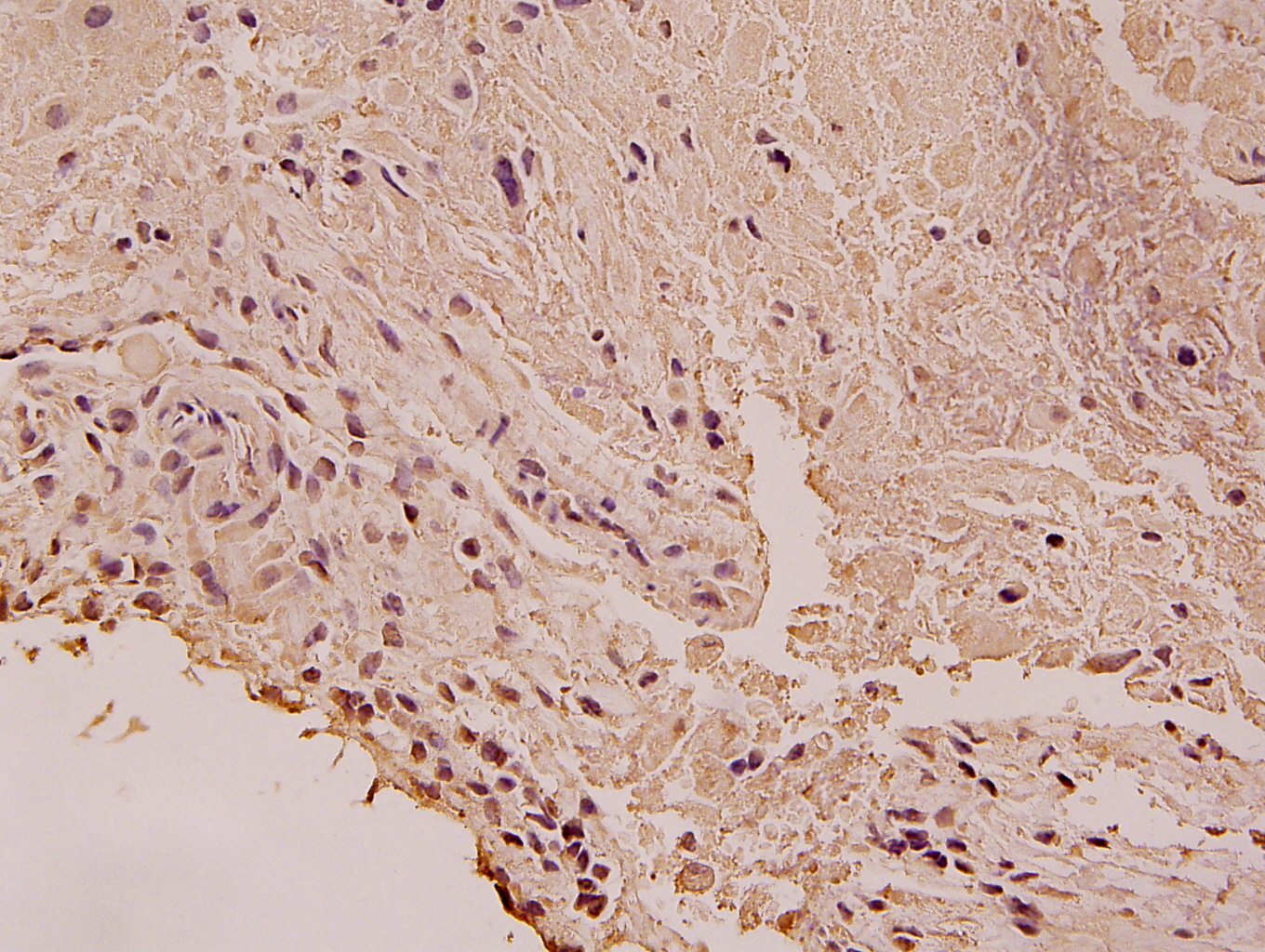

Supplement: S1 File — (ZIP) [file pone.0272499.s001.zip › supporting information/immunohistochemical/miR-31agomir CXCR4/5days/4.jpg]

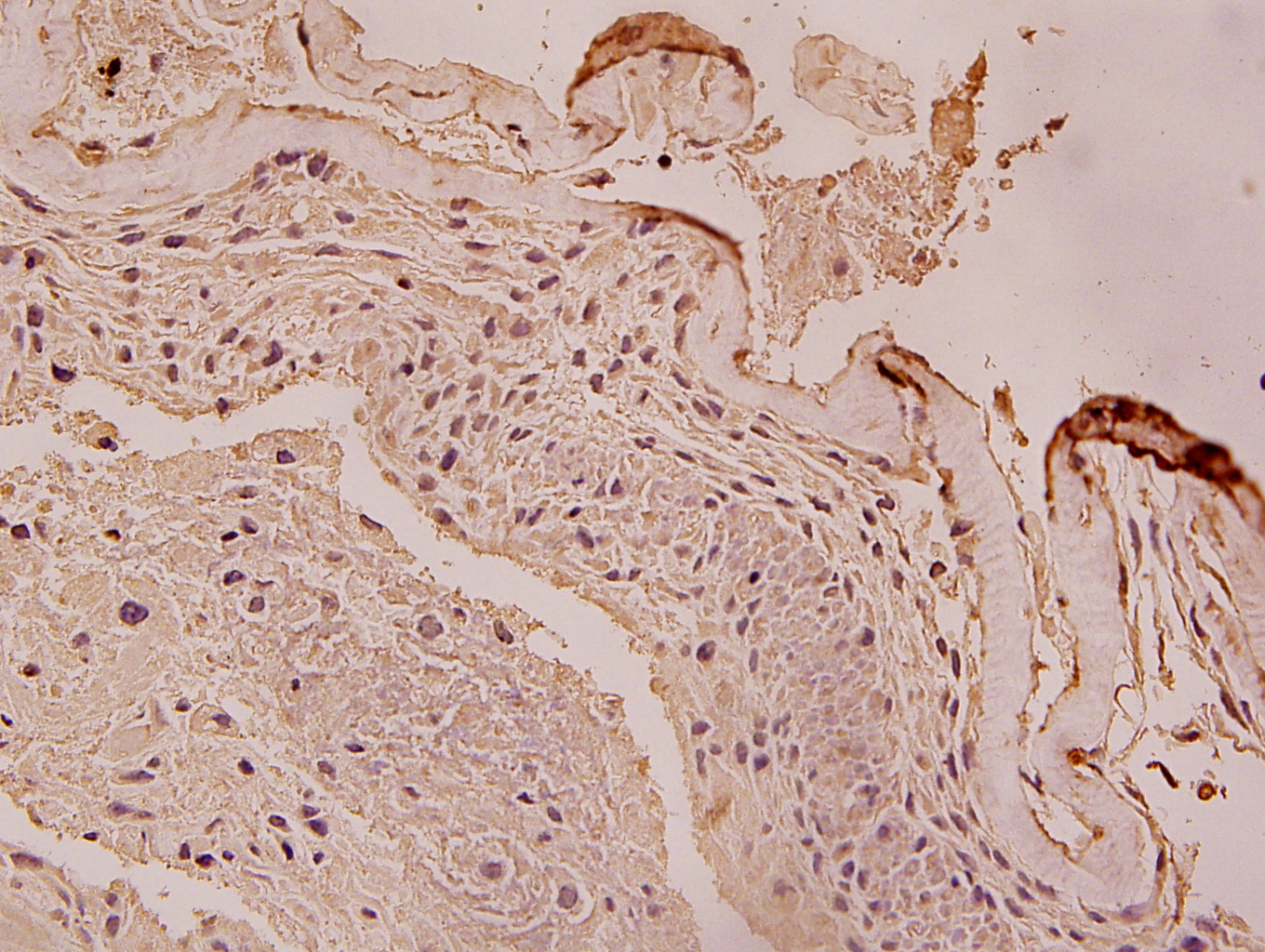

Supplement: S1 File — (ZIP) [file pone.0272499.s001.zip › supporting information/immunohistochemical/miR-31agomir CXCR4/5days/5.jpg]

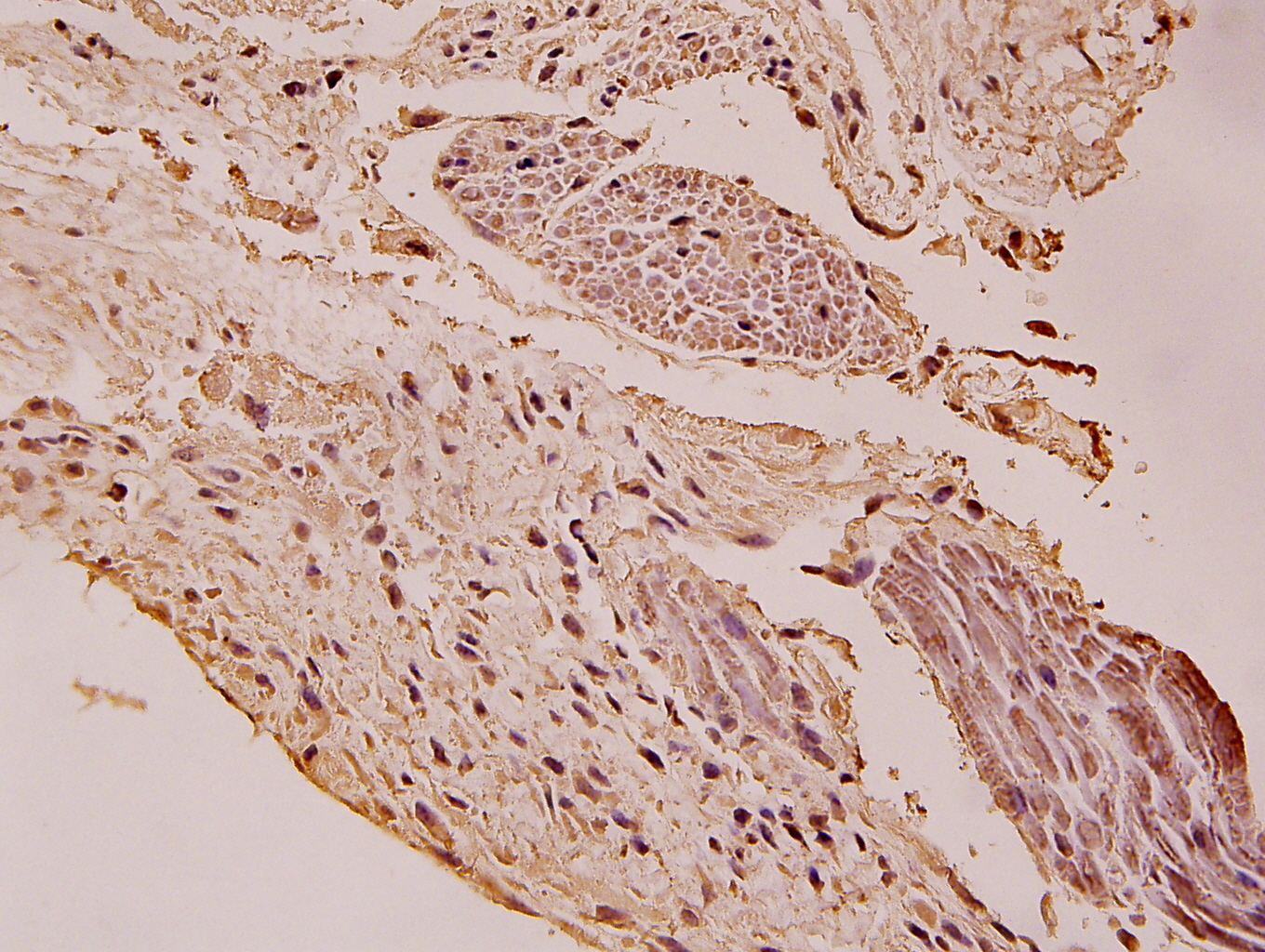

Supplement: S1 File — (ZIP) [file pone.0272499.s001.zip › supporting information/immunohistochemical/miR-31agomir CXCR4/7days/1.jpg]

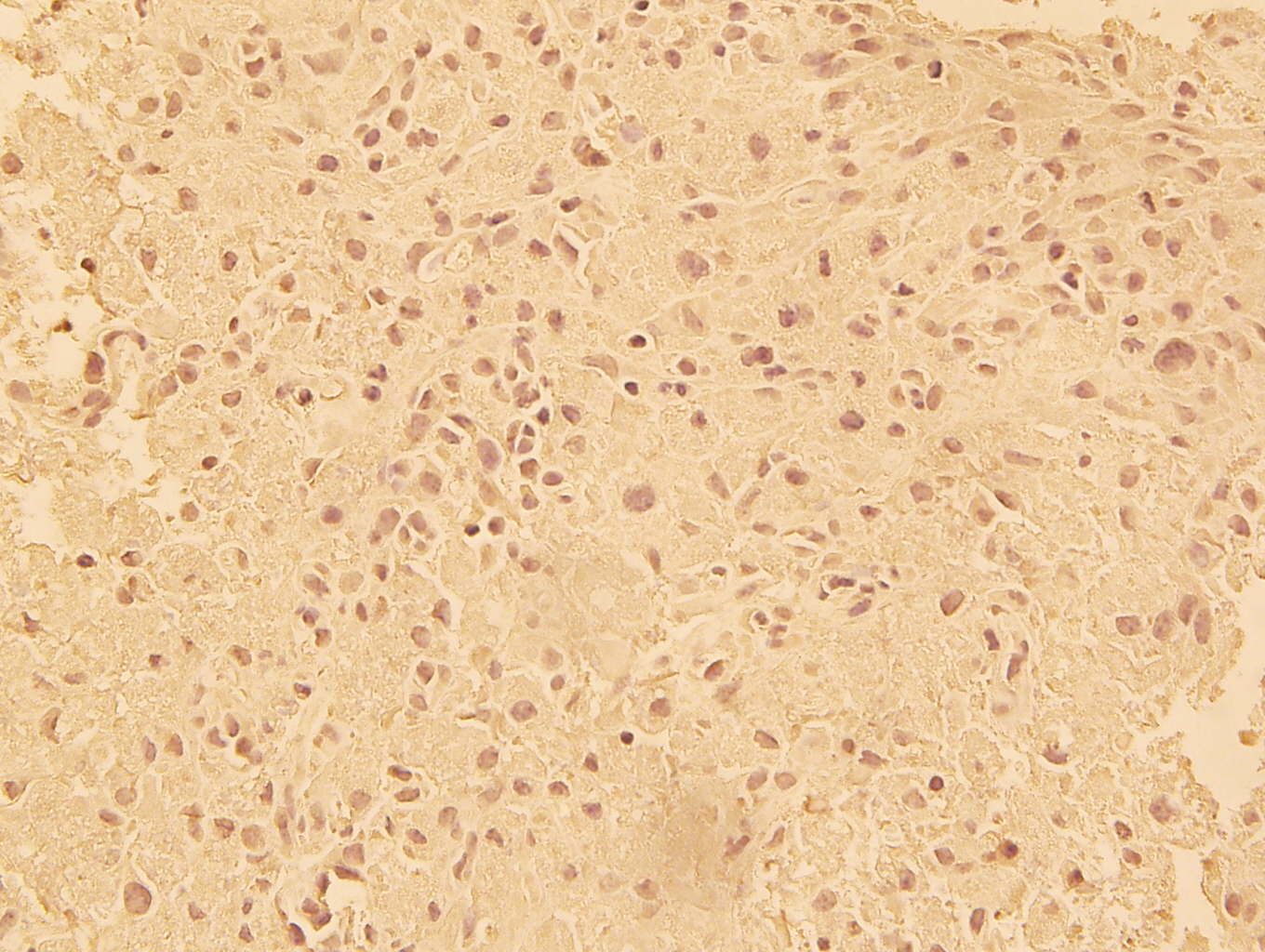

Supplement: S1 File — (ZIP) [file pone.0272499.s001.zip › supporting information/immunohistochemical/miR-31agomir CXCR4/7days/2.jpg]

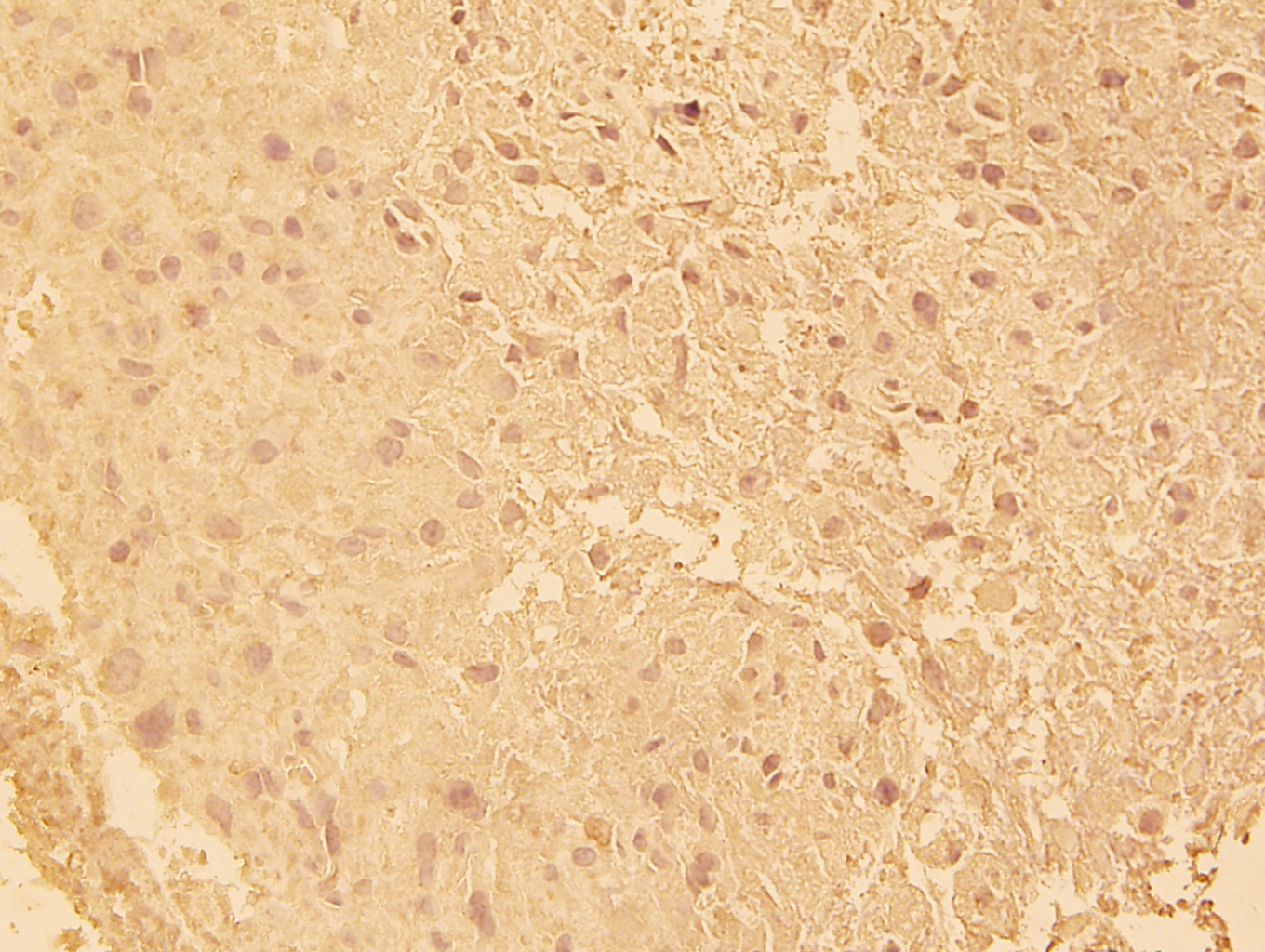

Supplement: S1 File — (ZIP) [file pone.0272499.s001.zip › supporting information/immunohistochemical/miR-31agomir CXCR4/7days/3.jpg]

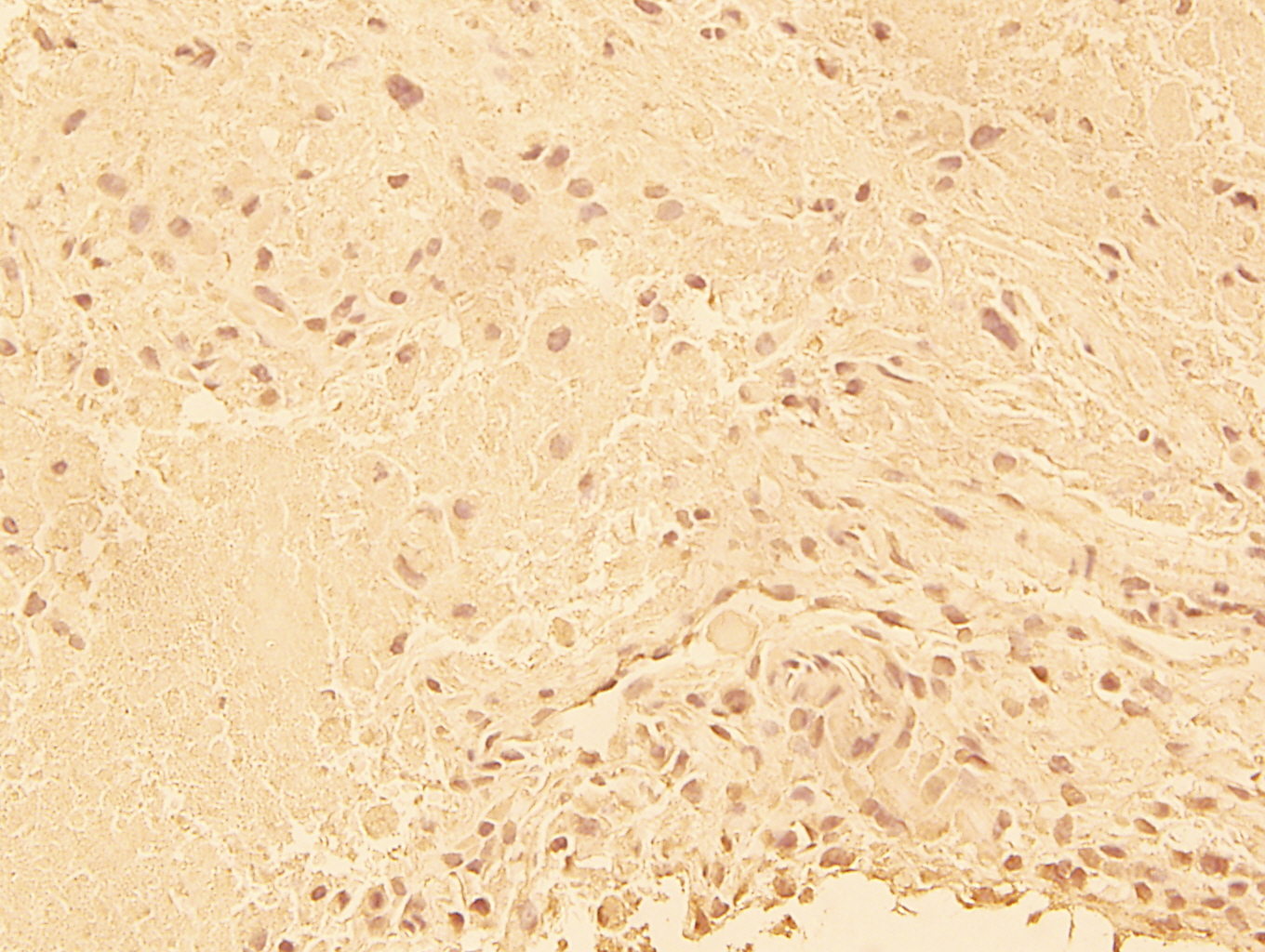

Supplement: S1 File — (ZIP) [file pone.0272499.s001.zip › supporting information/immunohistochemical/miR-31agomir CXCR4/7days/4.jpg]

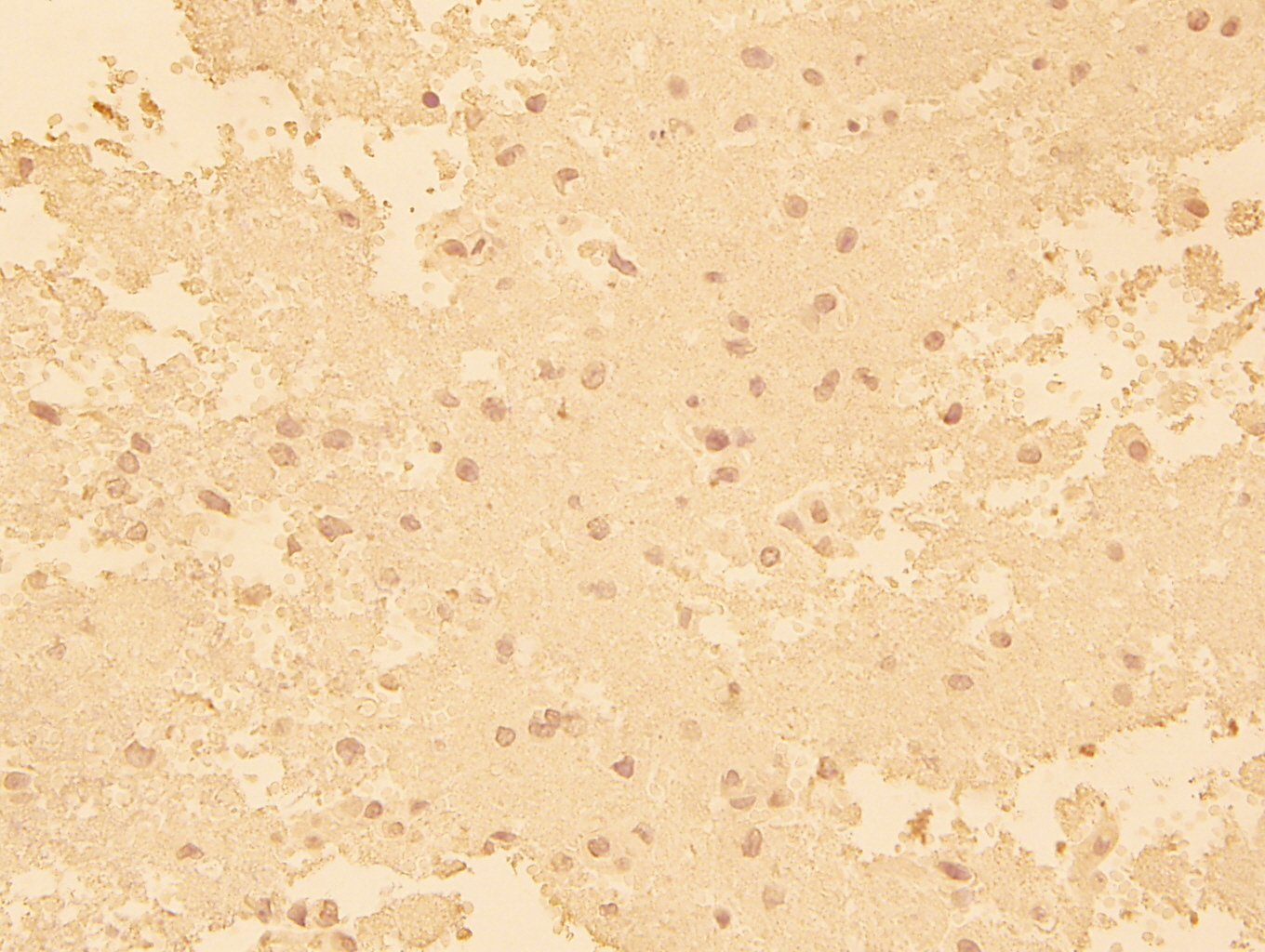

Supplement: S1 File — (ZIP) [file pone.0272499.s001.zip › supporting information/immunohistochemical/miR-31agomir CXCR4/7days/5.jpg]

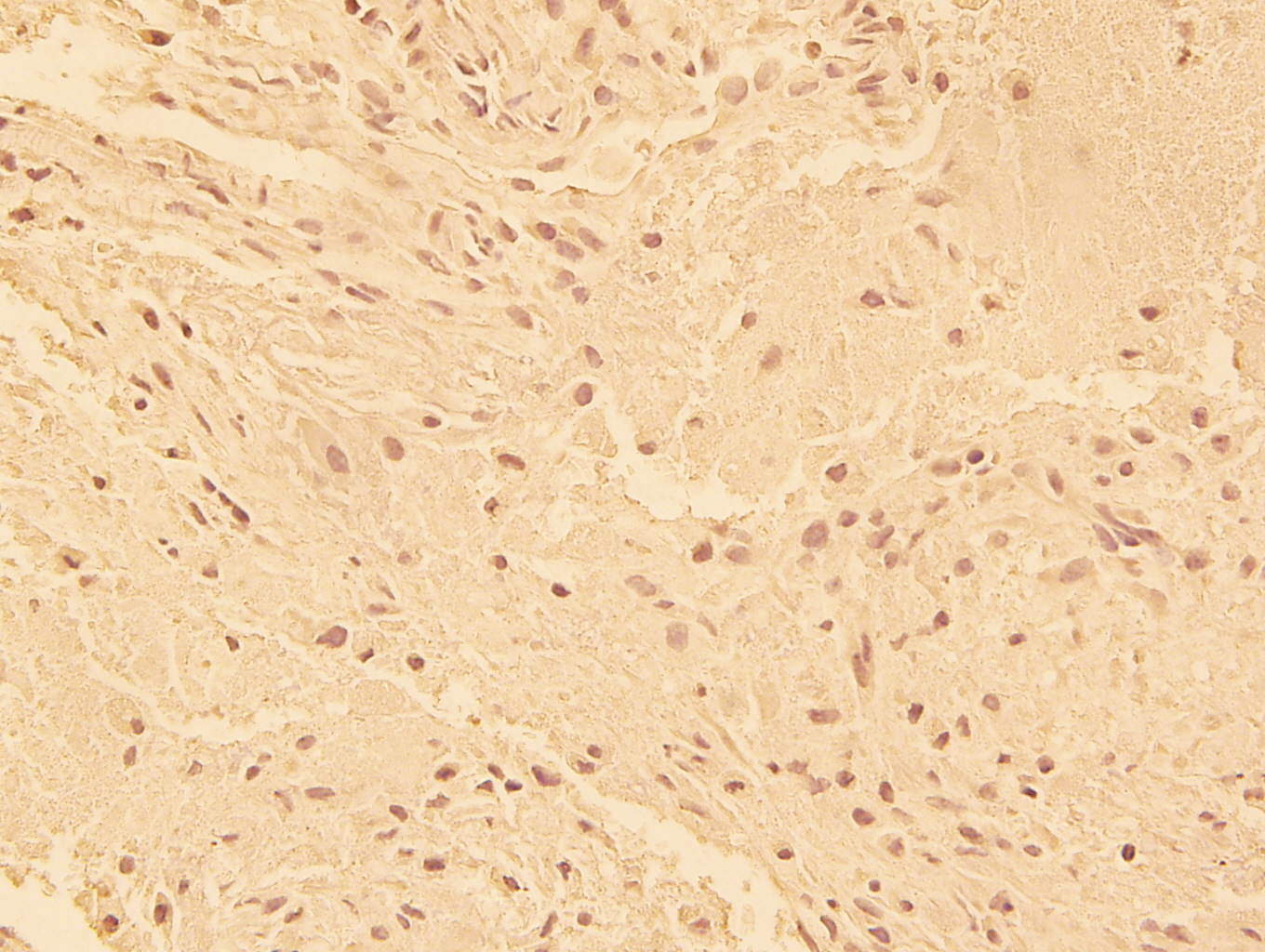

Supplement: S1 File — (ZIP) [file pone.0272499.s001.zip › supporting information/immunohistochemical/miR-31agomirMMP2/5days/1.jpg]

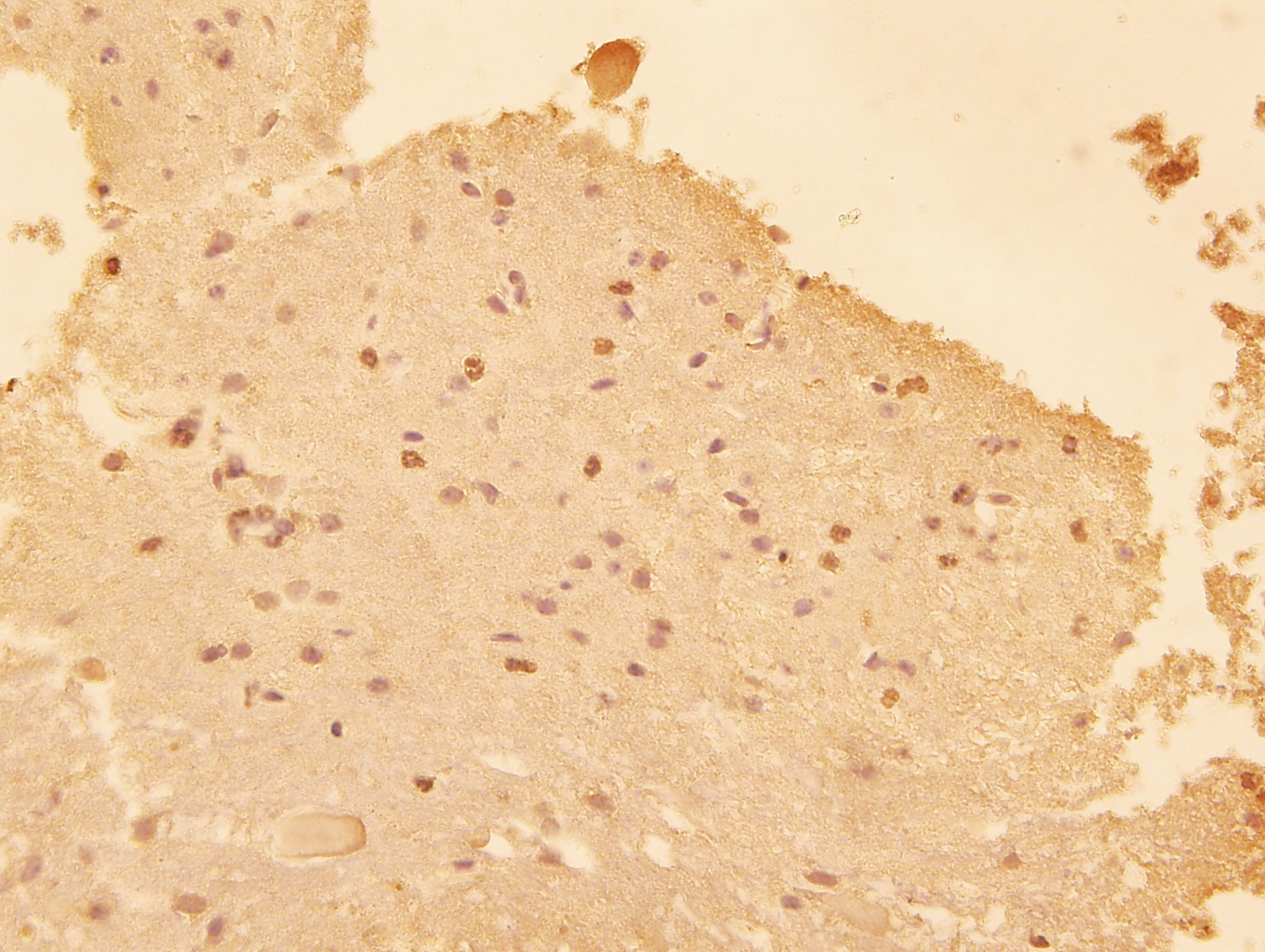

Supplement: S1 File — (ZIP) [file pone.0272499.s001.zip › supporting information/immunohistochemical/miR-31agomirMMP2/5days/2.jpg]

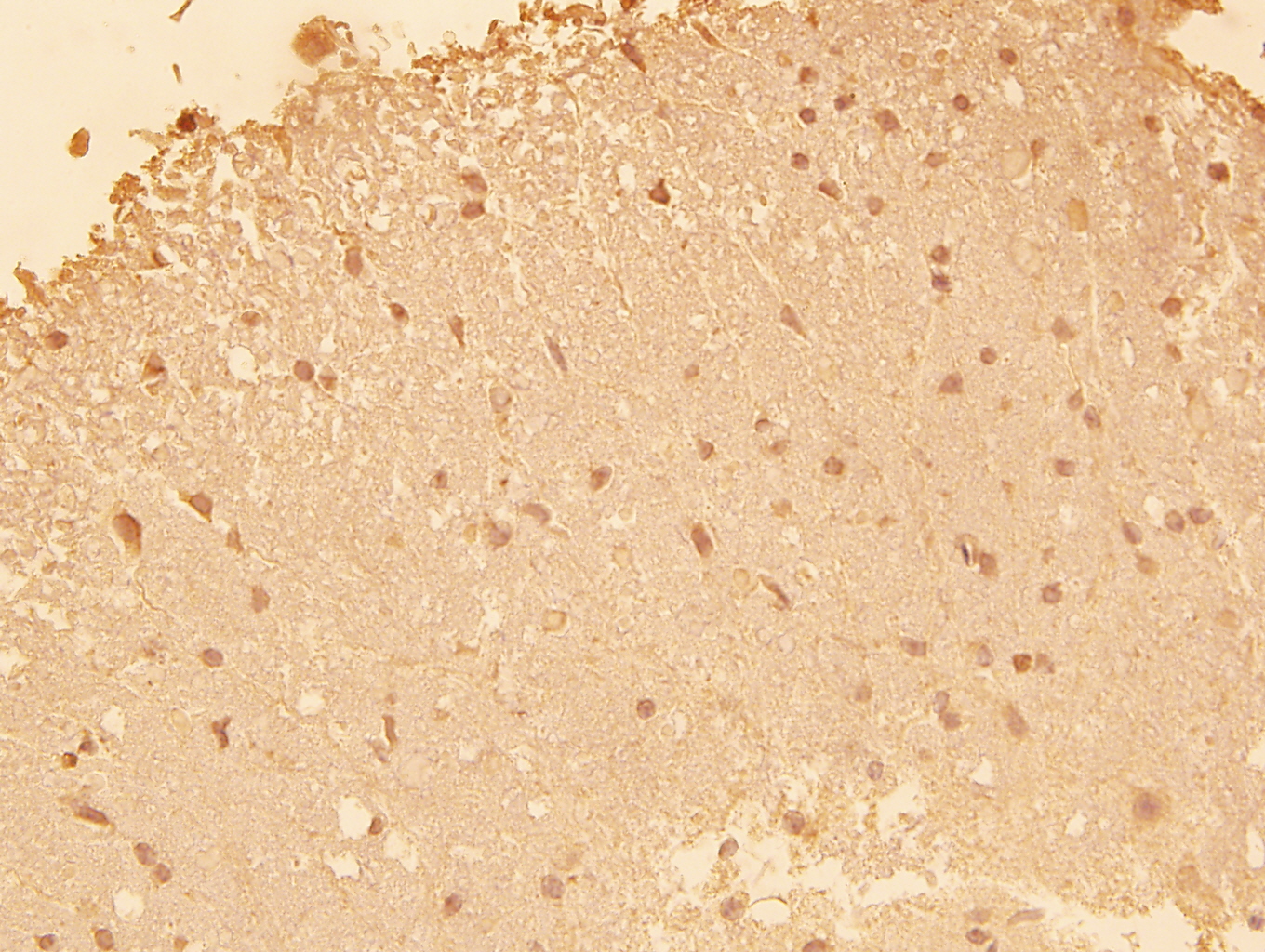

Supplement: S1 File — (ZIP) [file pone.0272499.s001.zip › supporting information/immunohistochemical/miR-31agomirMMP2/5days/3.jpg]

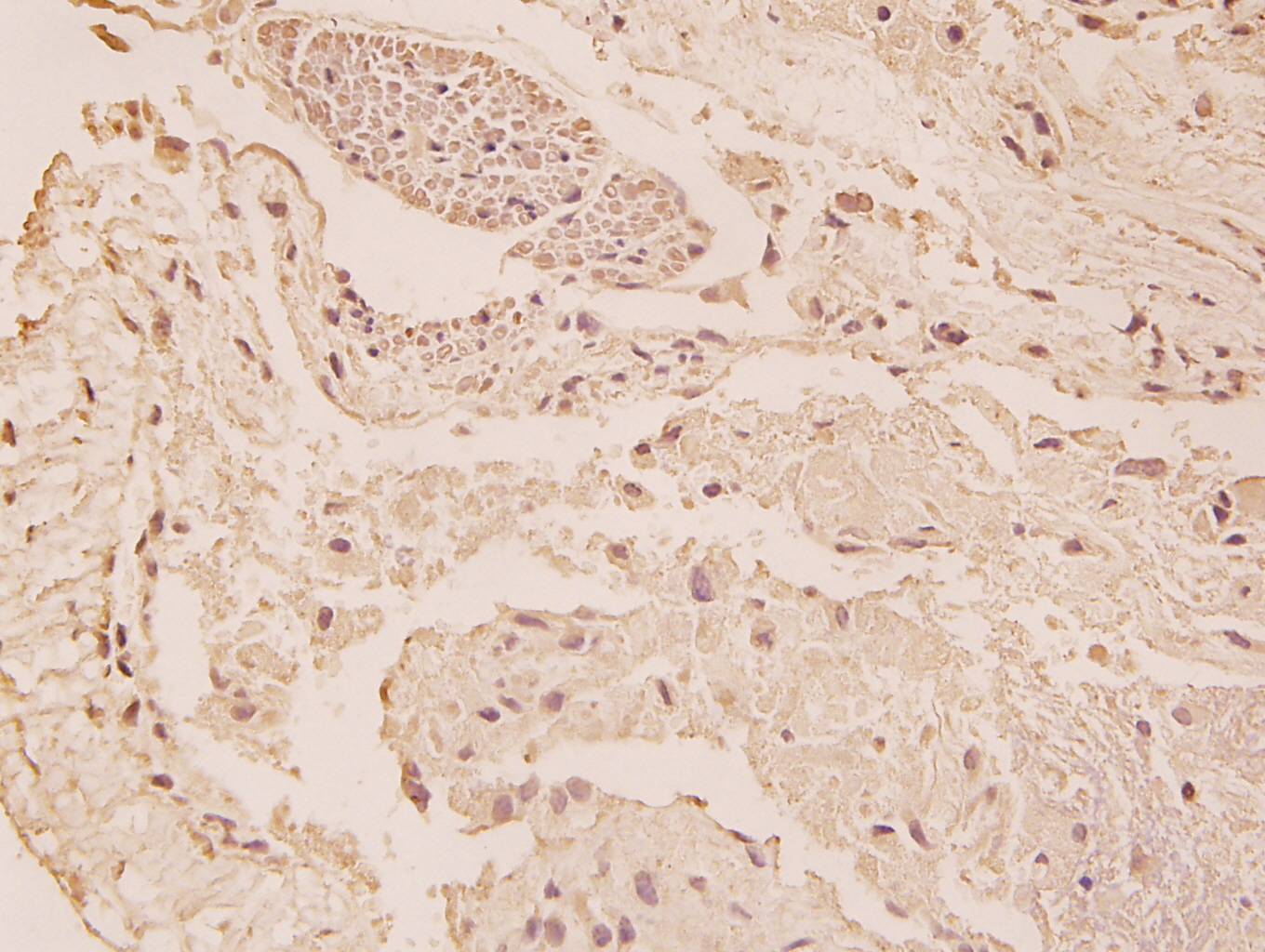

Supplement: S1 File — (ZIP) [file pone.0272499.s001.zip › supporting information/immunohistochemical/miR-31agomirMMP2/5days/4.jpg]

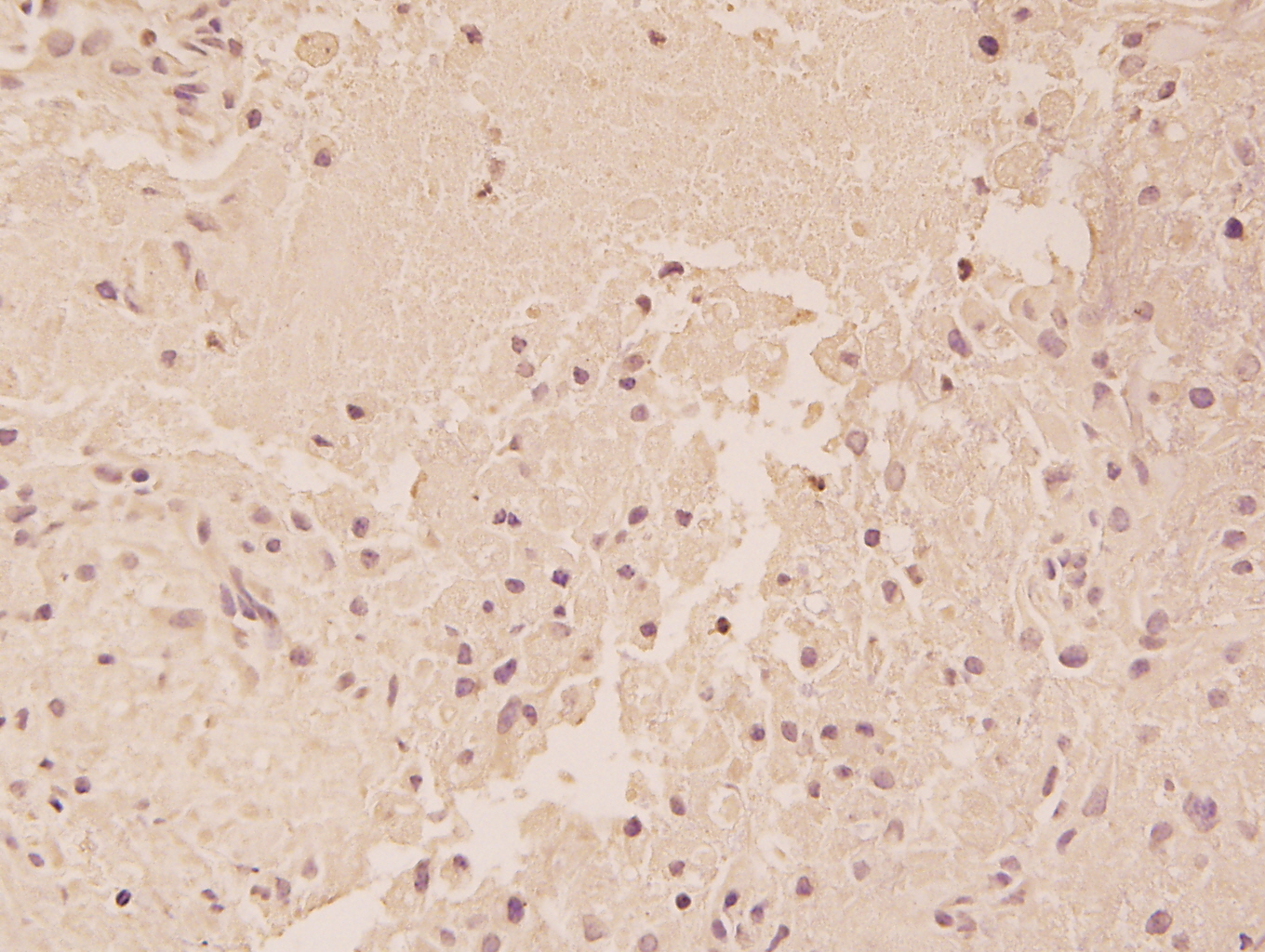

Supplement: S1 File — (ZIP) [file pone.0272499.s001.zip › supporting information/immunohistochemical/miR-31agomirMMP2/5days/5.jpg]

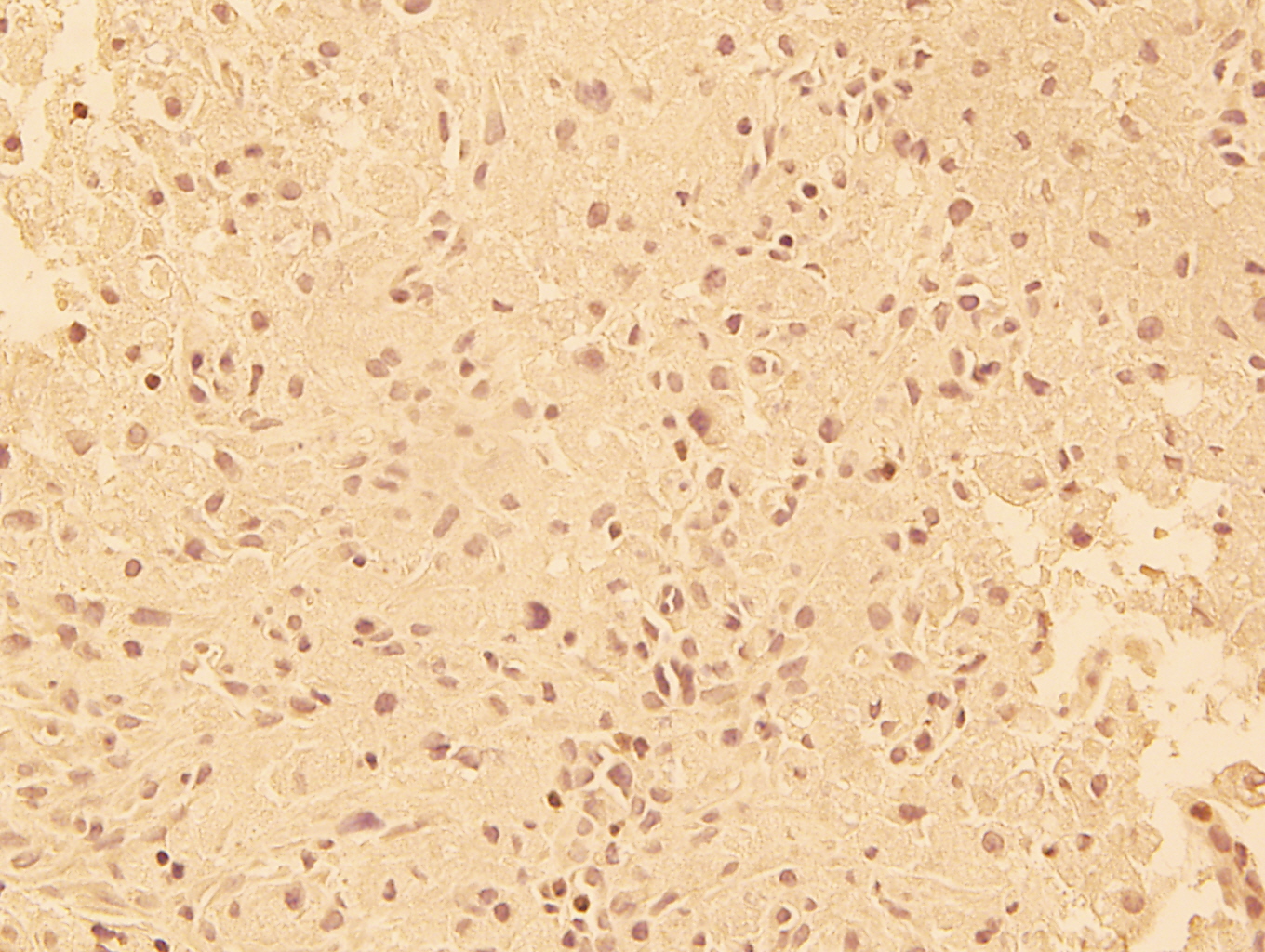

Supplement: S1 File — (ZIP) [file pone.0272499.s001.zip › supporting information/immunohistochemical/miR-31agomirMMP2/7days/1.jpg]

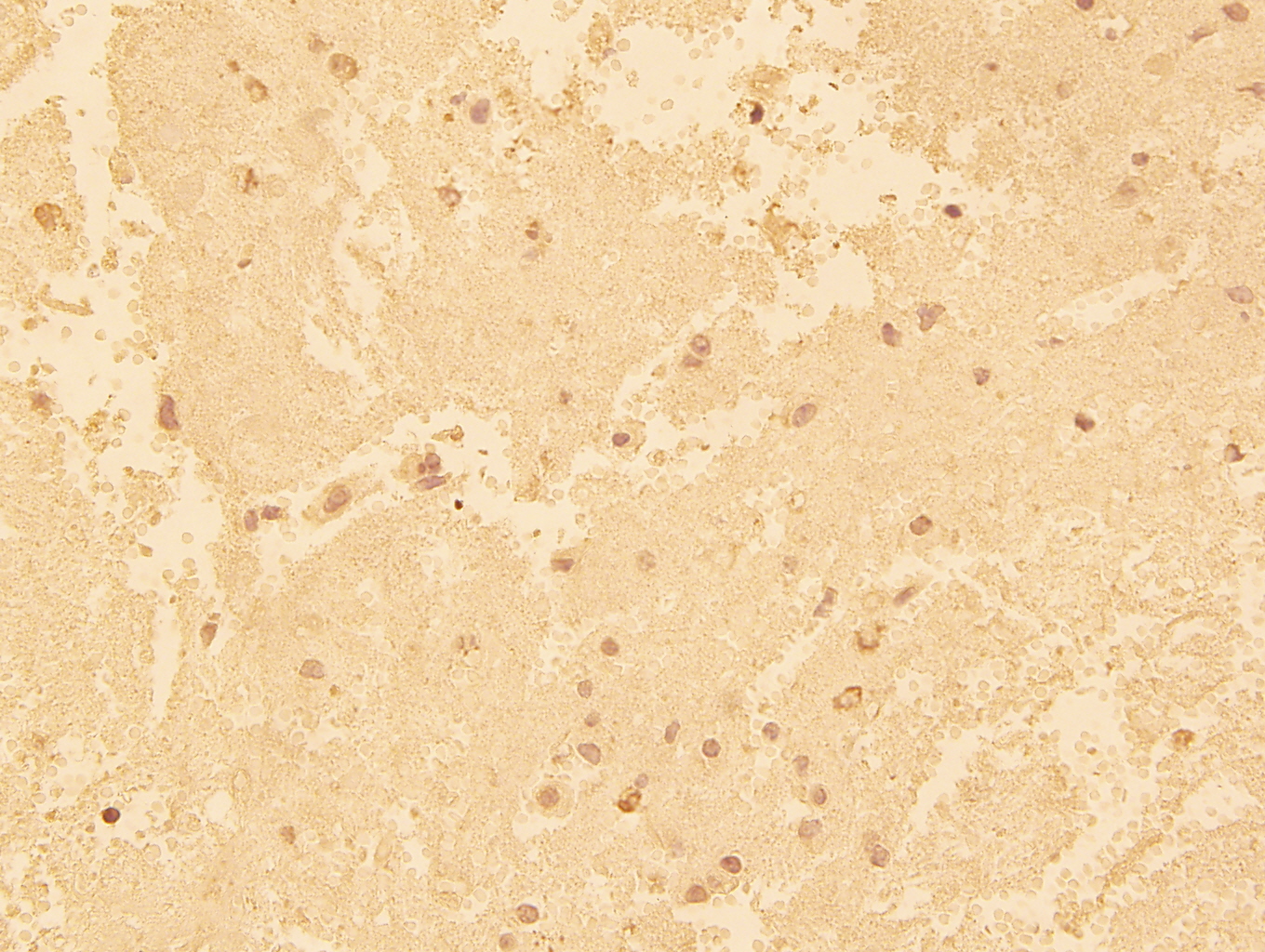

Supplement: S1 File — (ZIP) [file pone.0272499.s001.zip › supporting information/immunohistochemical/miR-31agomirMMP2/7days/2.jpg]

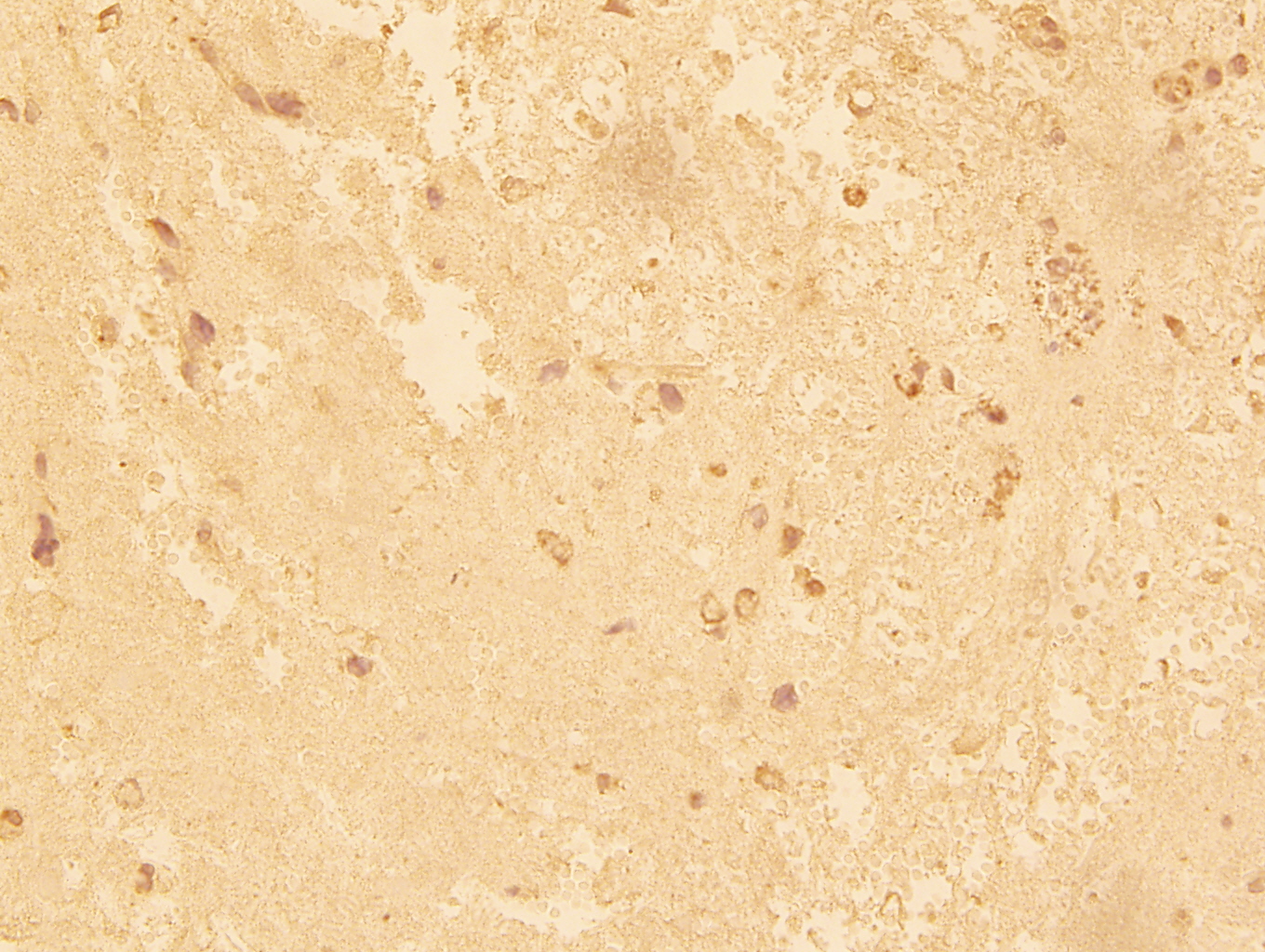

Supplement: S1 File — (ZIP) [file pone.0272499.s001.zip › supporting information/immunohistochemical/miR-31agomirMMP2/7days/3.jpg]

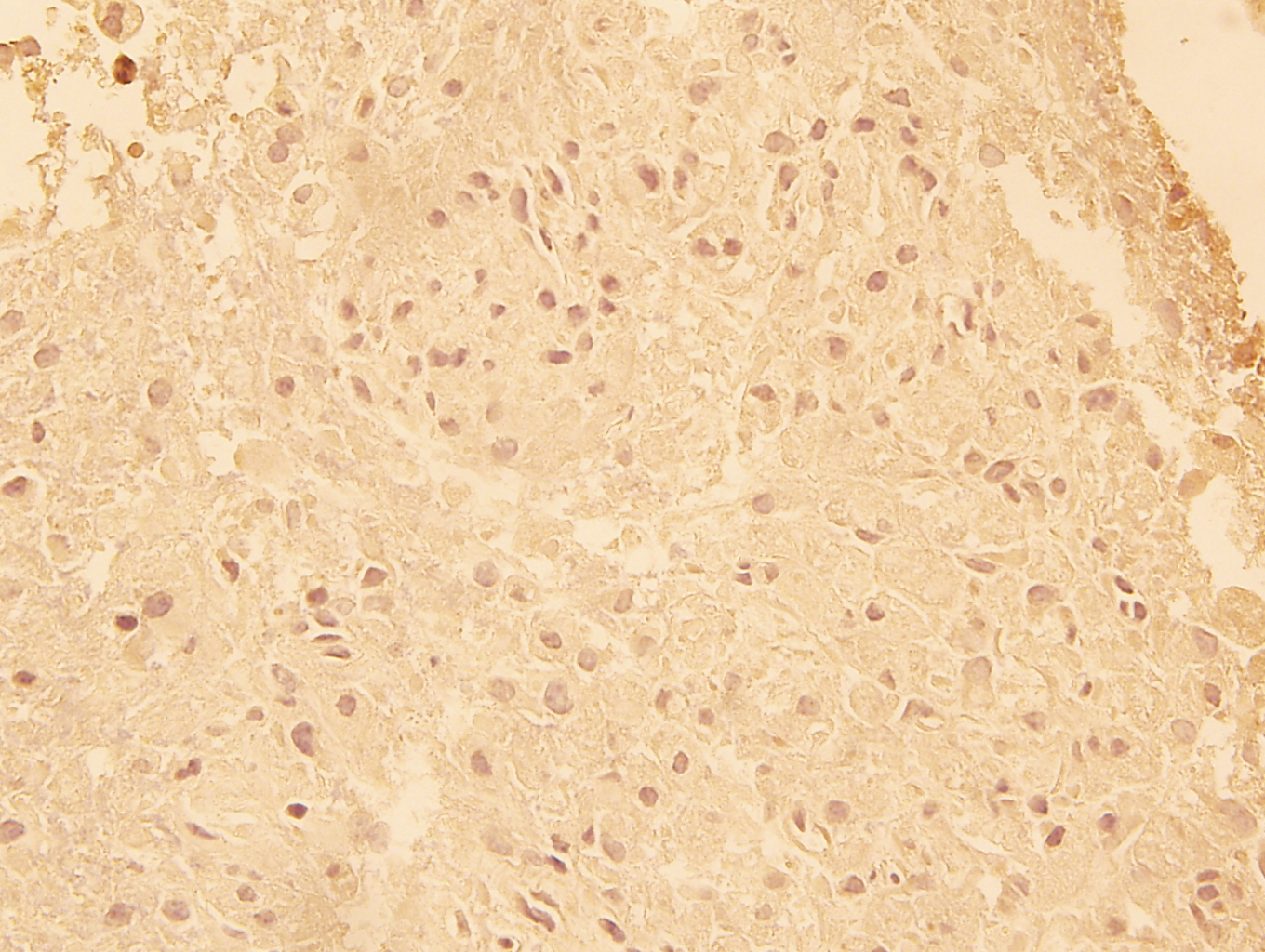

Supplement: S1 File — (ZIP) [file pone.0272499.s001.zip › supporting information/immunohistochemical/miR-31agomirMMP2/7days/4.jpg]

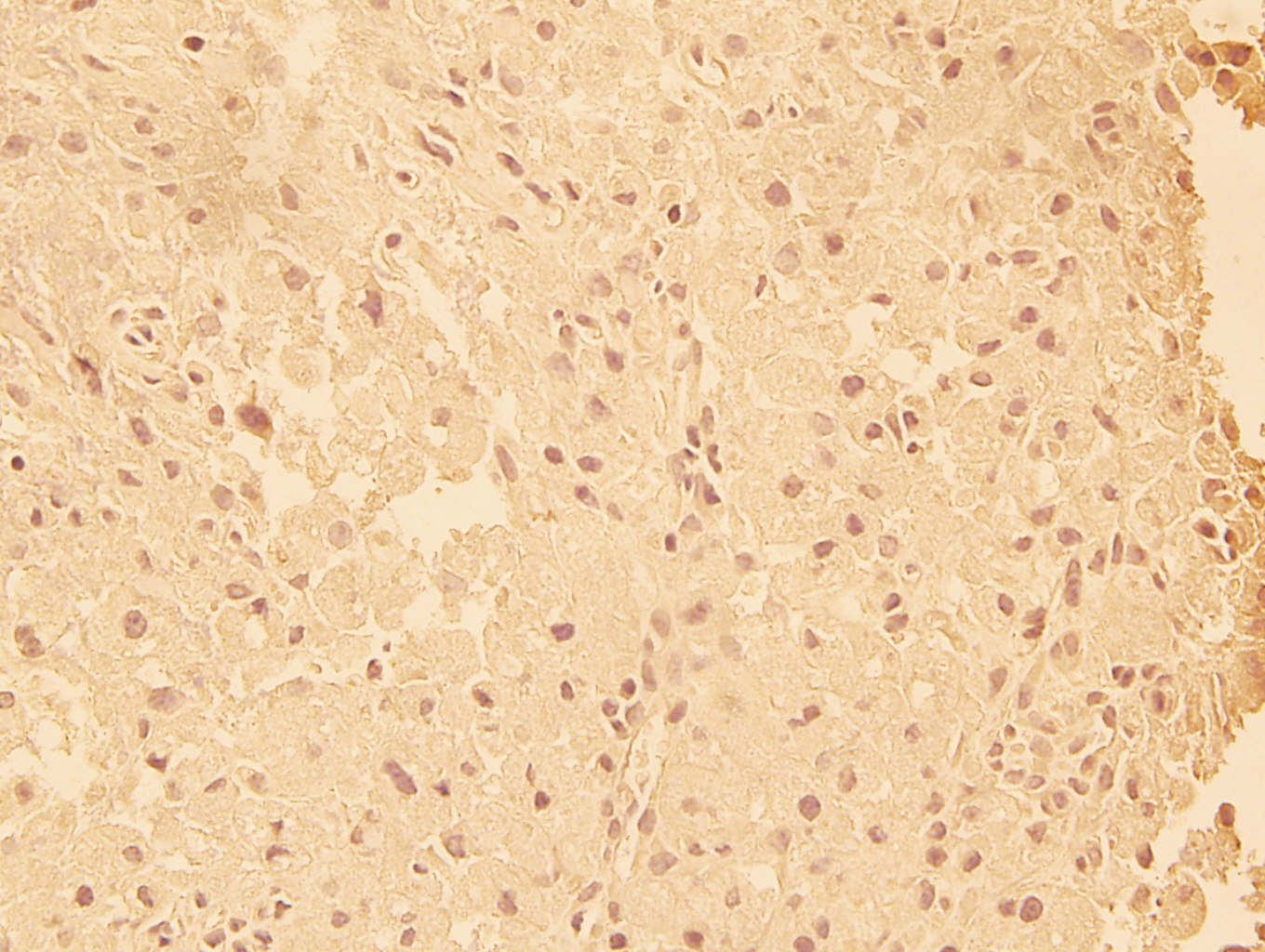

Supplement: S1 File — (ZIP) [file pone.0272499.s001.zip › supporting information/immunohistochemical/miR-31agomirMMP2/7days/5.jpg]

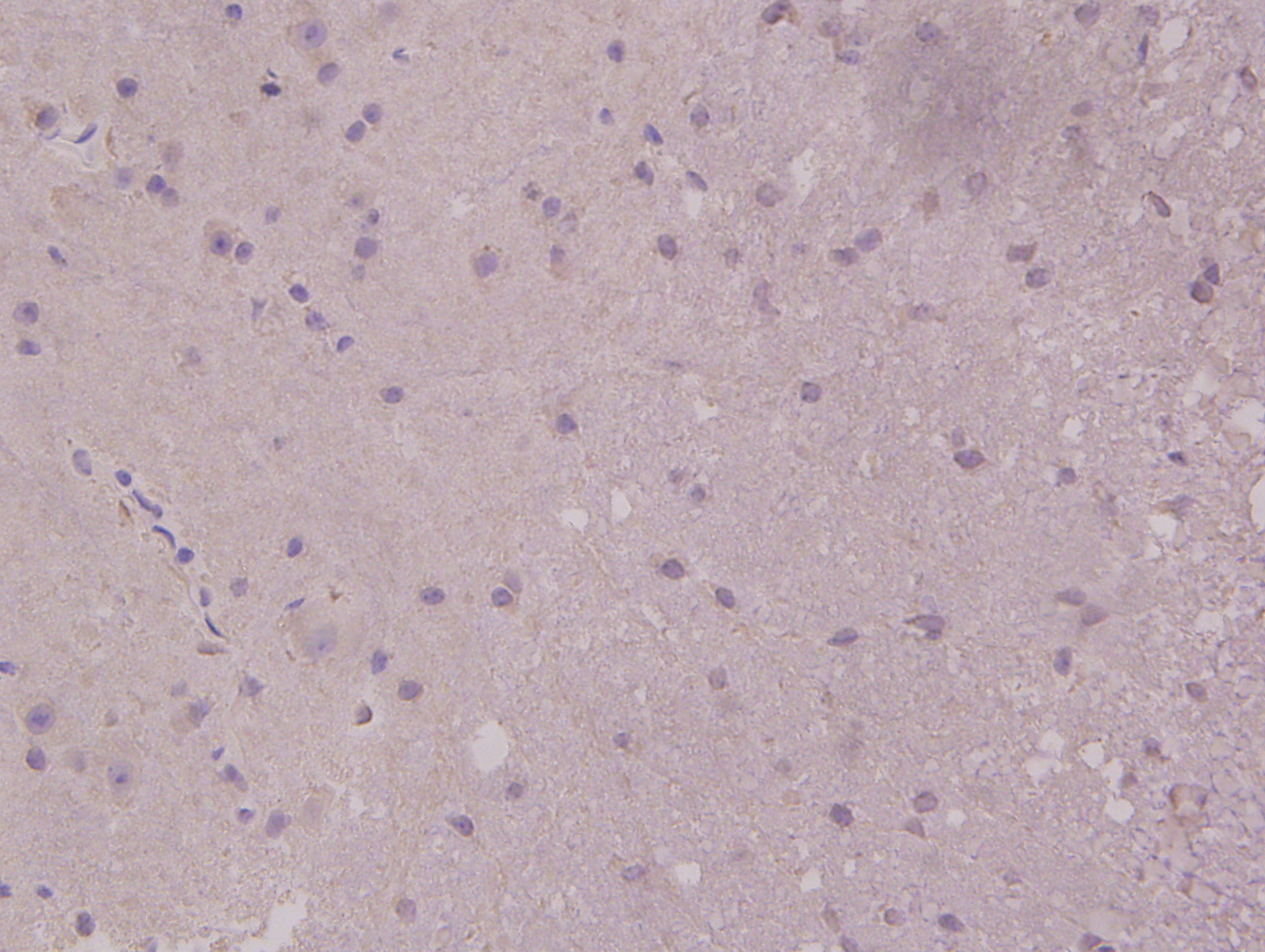

Supplement: S1 File — (ZIP) [file pone.0272499.s001.zip › supporting information/immunohistochemical/miR-31antagomirCXCR4/5days/1.jpg]

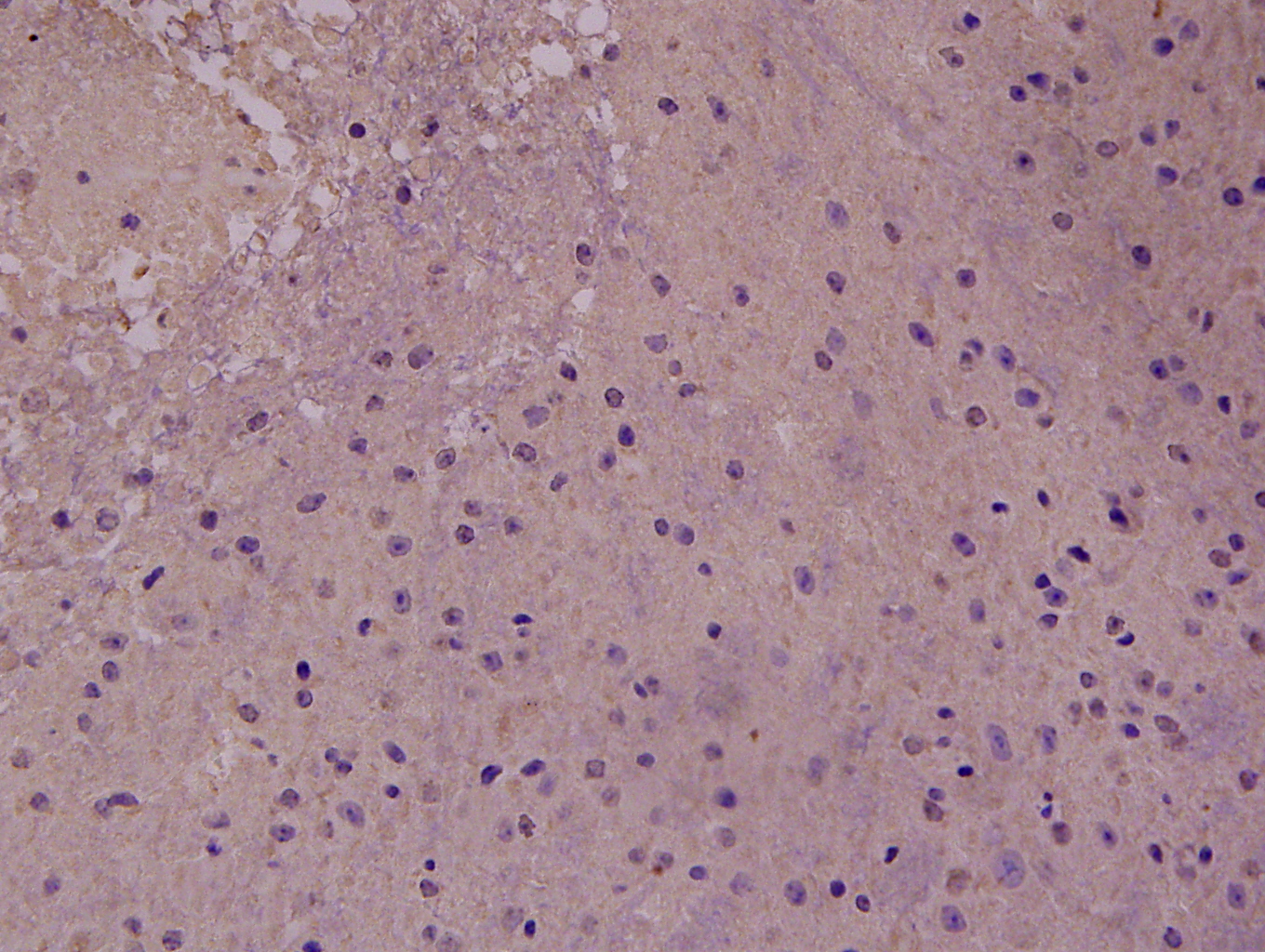

Supplement: S1 File — (ZIP) [file pone.0272499.s001.zip › supporting information/immunohistochemical/miR-31antagomirCXCR4/5days/2.jpg]

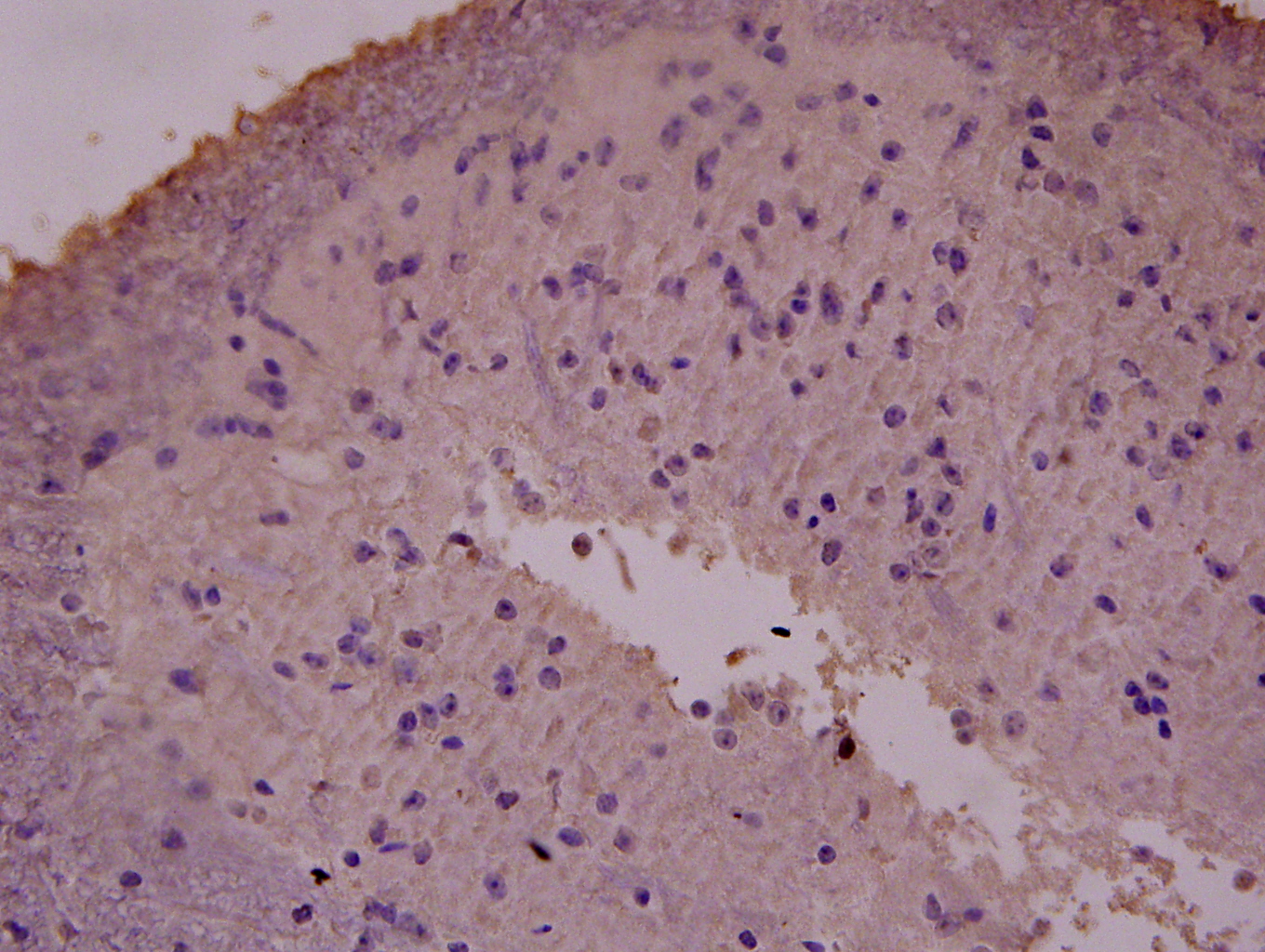

Supplement: S1 File — (ZIP) [file pone.0272499.s001.zip › supporting information/immunohistochemical/miR-31antagomirCXCR4/5days/3.jpg]

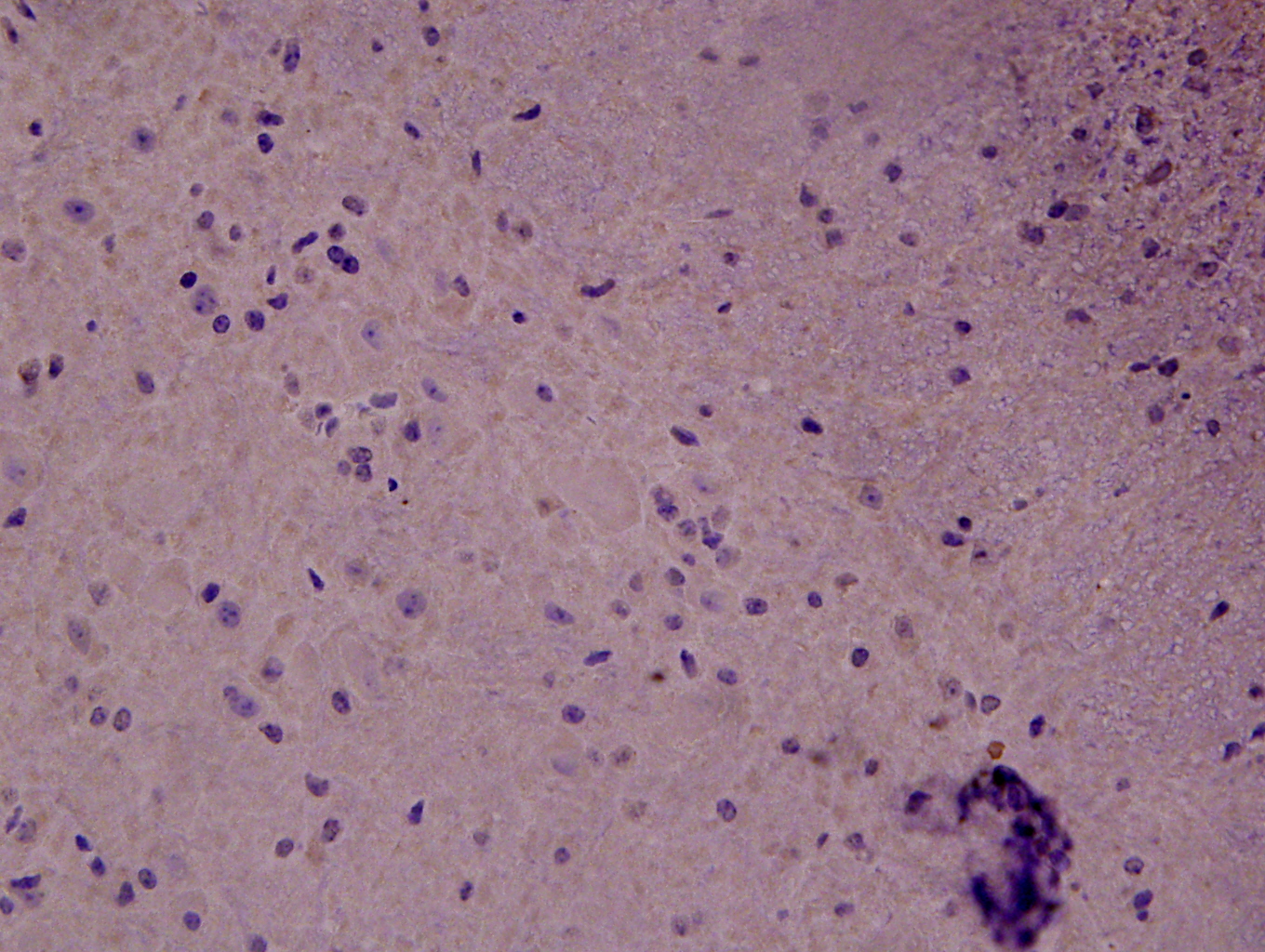

Supplement: S1 File — (ZIP) [file pone.0272499.s001.zip › supporting information/immunohistochemical/miR-31antagomirCXCR4/5days/4.jpg]

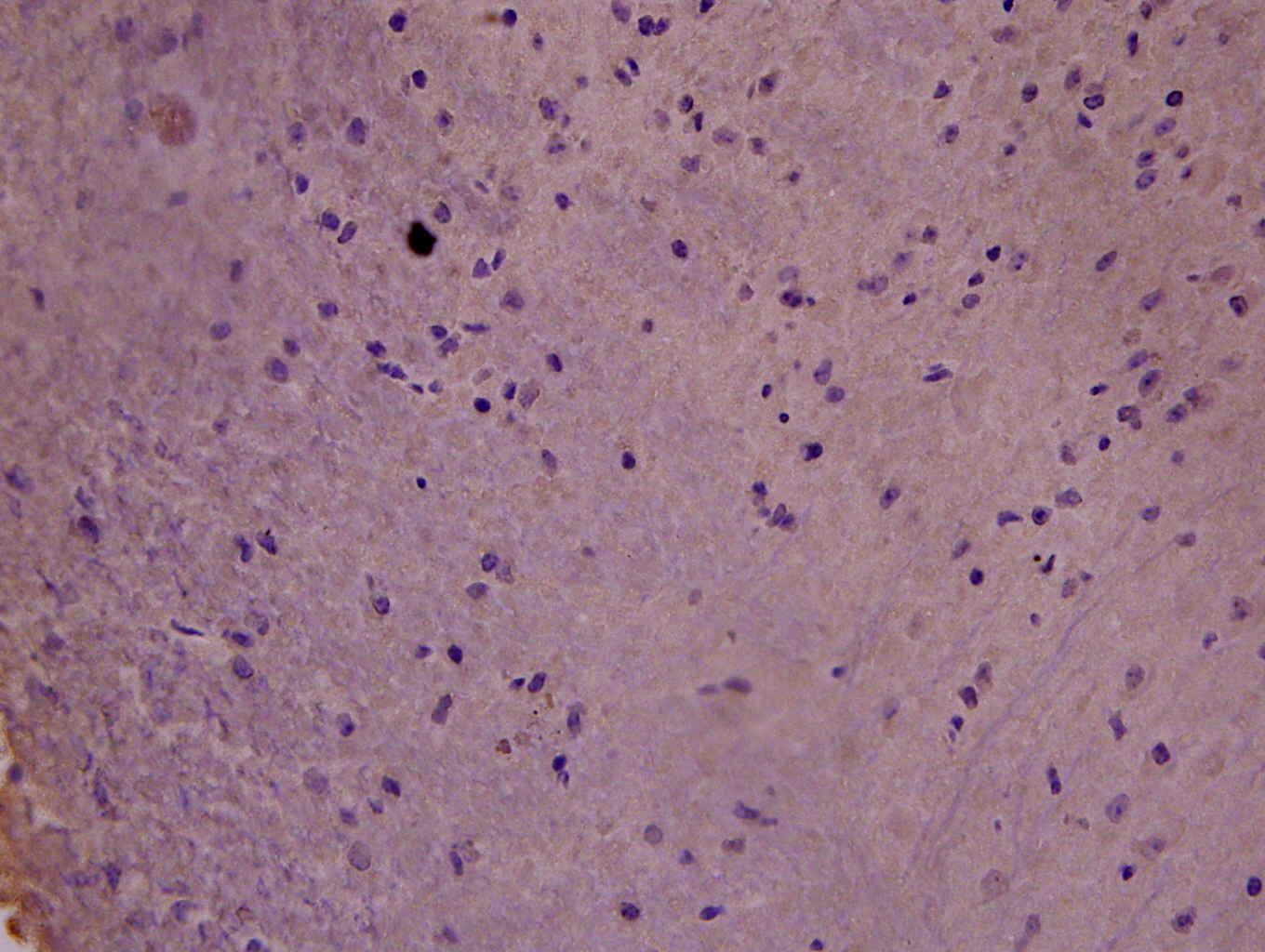

Supplement: S1 File — (ZIP) [file pone.0272499.s001.zip › supporting information/immunohistochemical/miR-31antagomirCXCR4/5days/5.jpg]

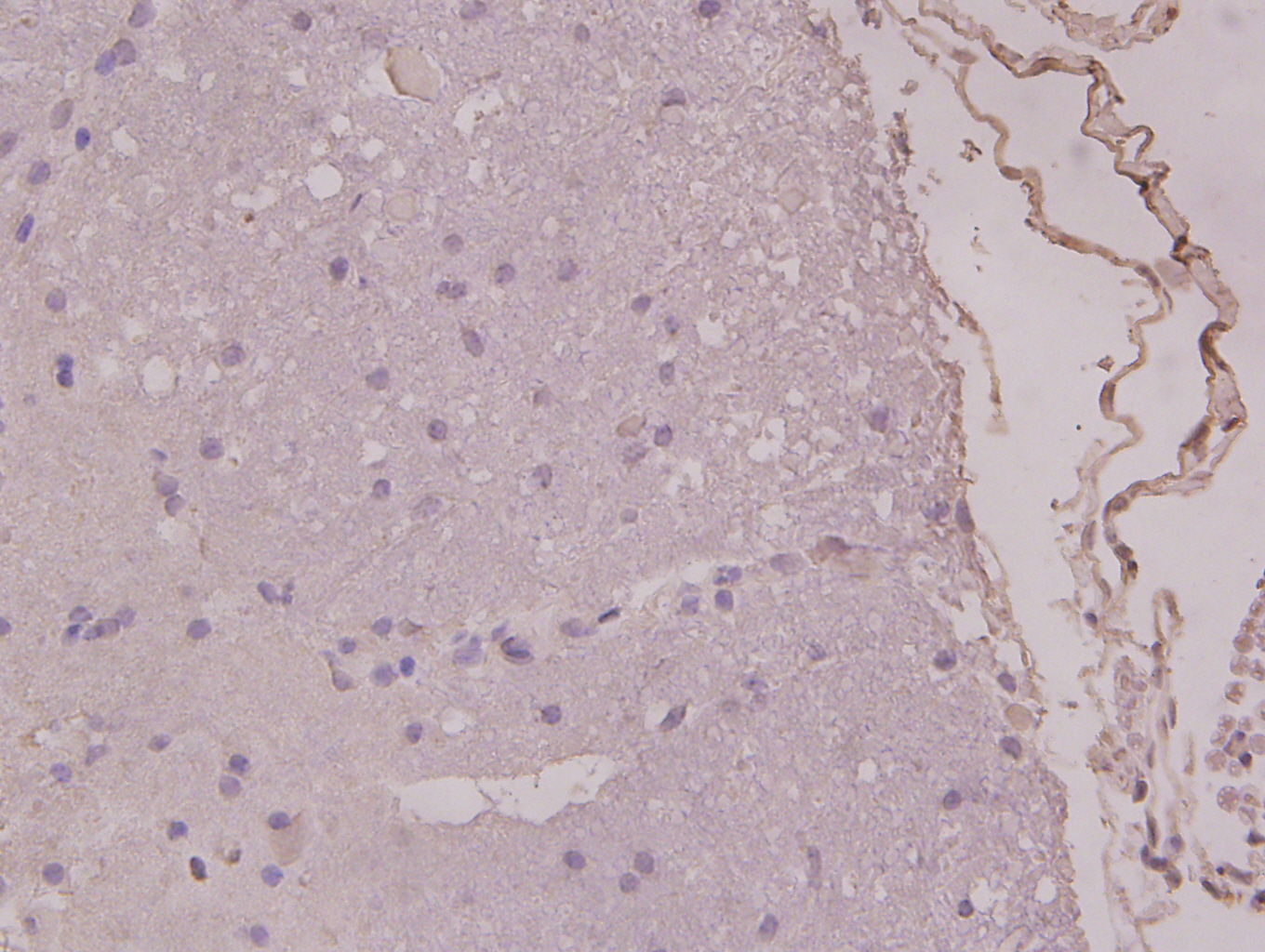

Supplement: S1 File — (ZIP) [file pone.0272499.s001.zip › supporting information/immunohistochemical/miR-31antagomirCXCR4/7days/1.jpg]

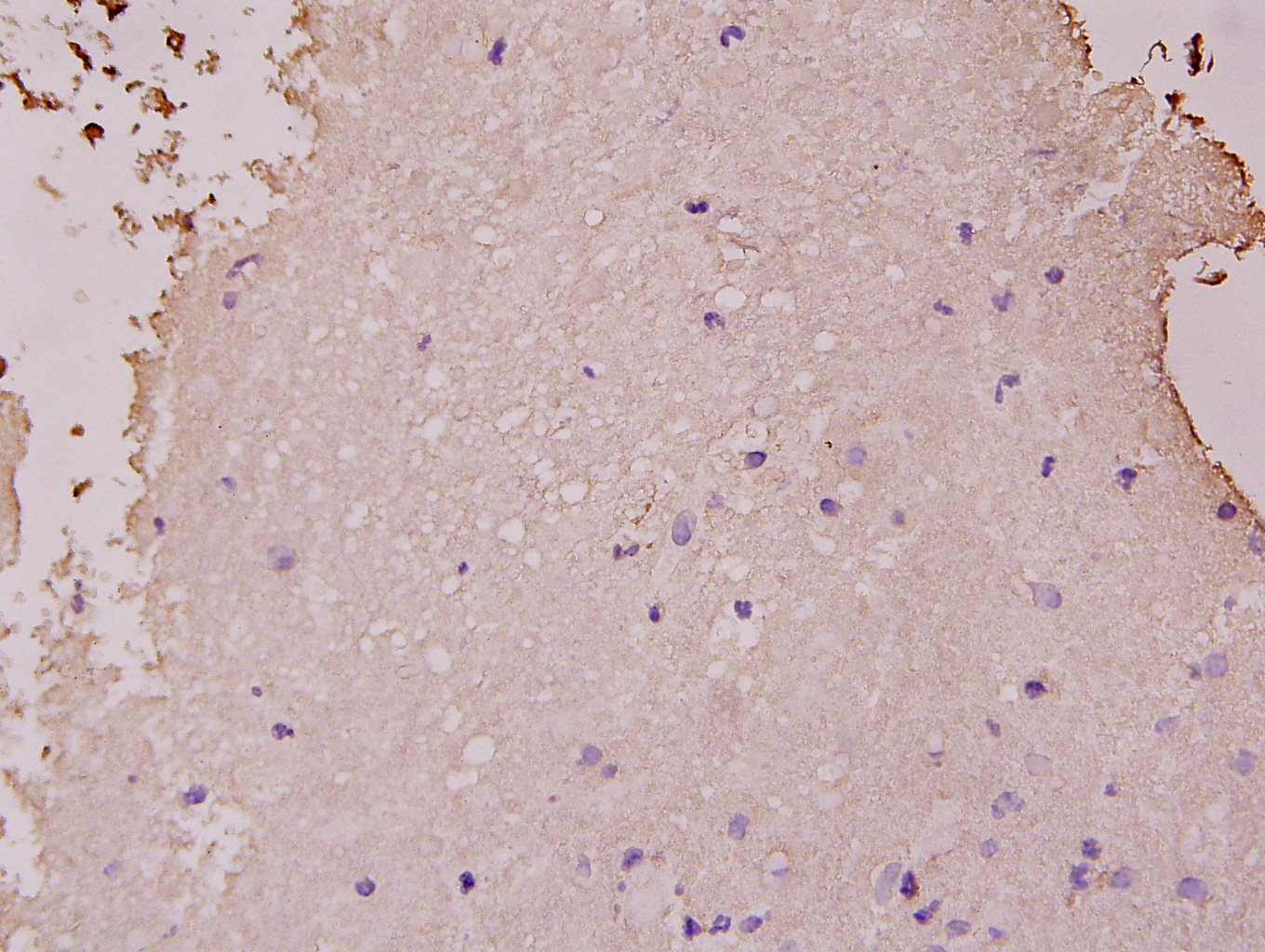

Supplement: S1 File — (ZIP) [file pone.0272499.s001.zip › supporting information/immunohistochemical/miR-31antagomirCXCR4/7days/2.jpg]

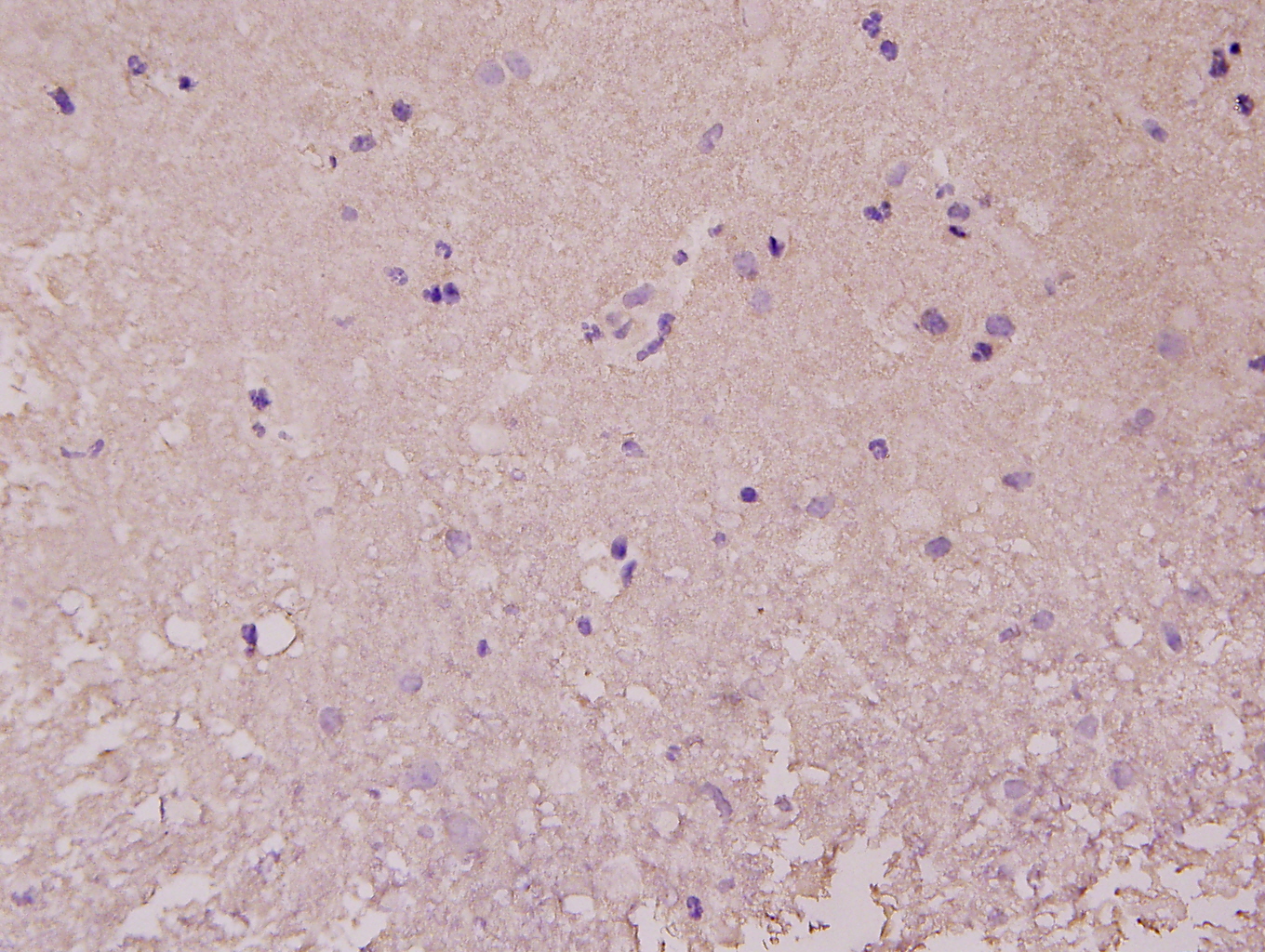

Supplement: S1 File — (ZIP) [file pone.0272499.s001.zip › supporting information/immunohistochemical/miR-31antagomirCXCR4/7days/3.jpg]

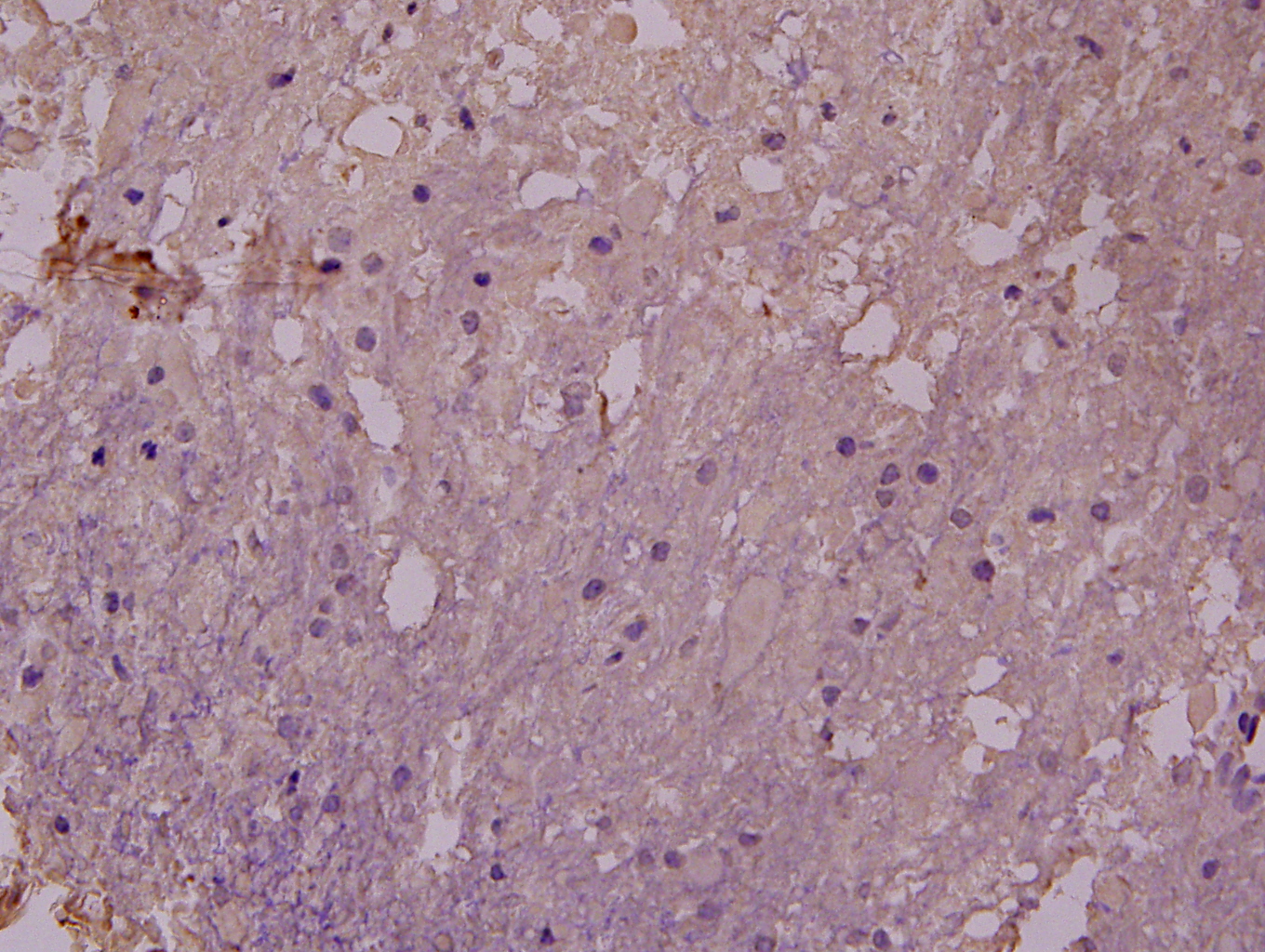

Supplement: S1 File — (ZIP) [file pone.0272499.s001.zip › supporting information/immunohistochemical/miR-31antagomirCXCR4/7days/4.jpg]

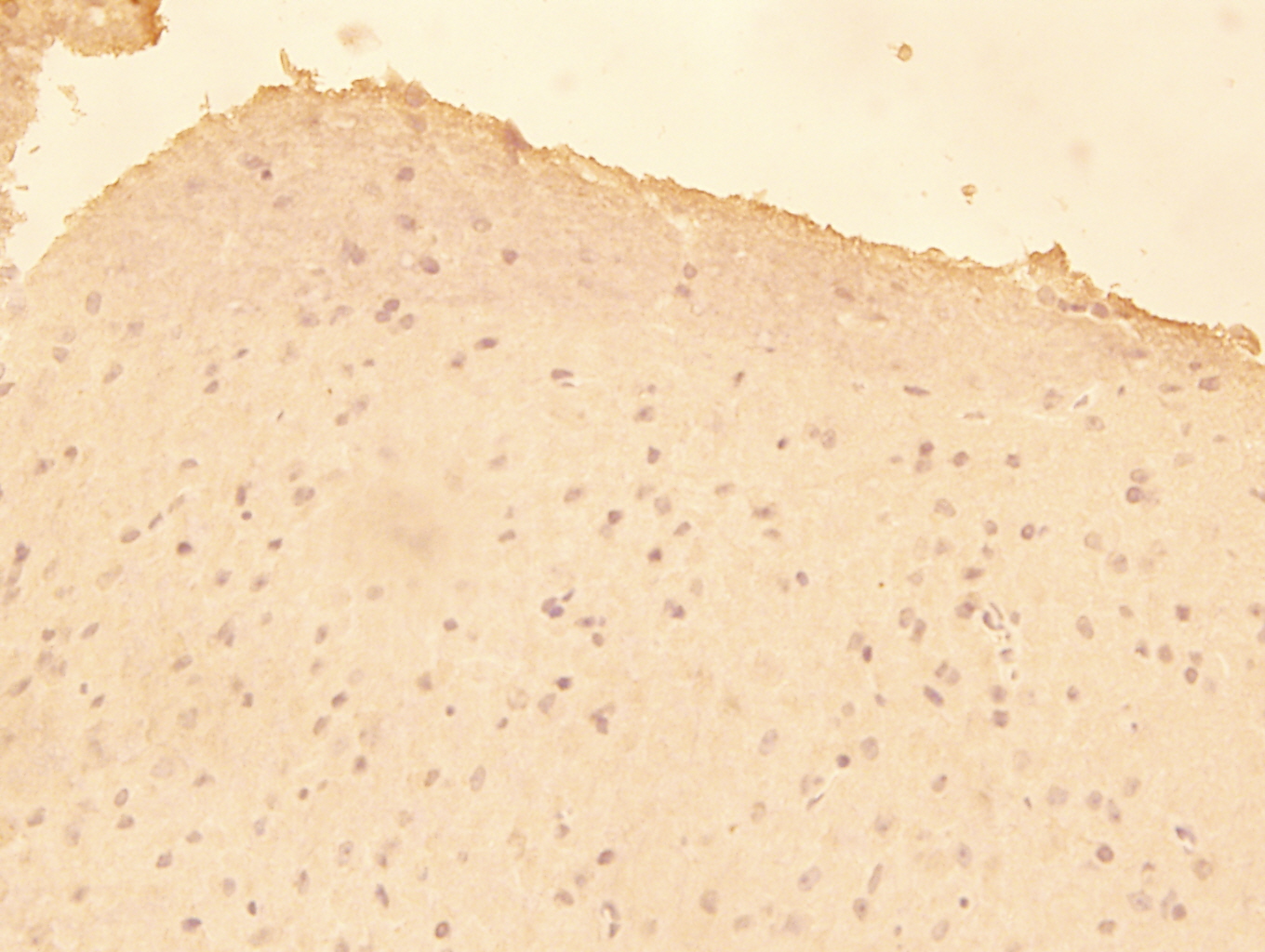

Supplement: S1 File — (ZIP) [file pone.0272499.s001.zip › supporting information/immunohistochemical/miR-31antagomirCXCR4/7days/5.jpg]

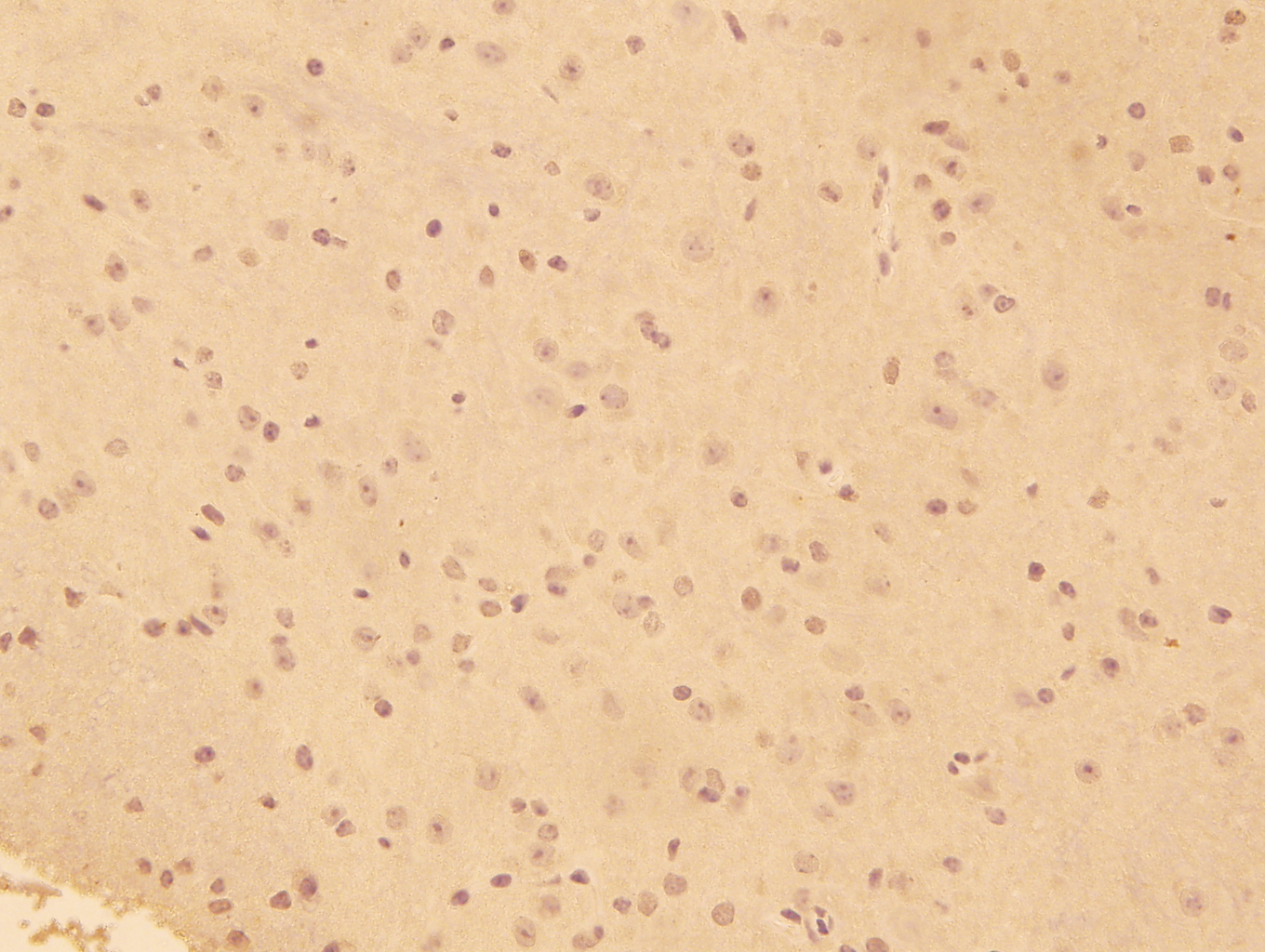

Supplement: S1 File — (ZIP) [file pone.0272499.s001.zip › supporting information/immunohistochemical/miR-31antagomirMMP2/5days/1.jpg]

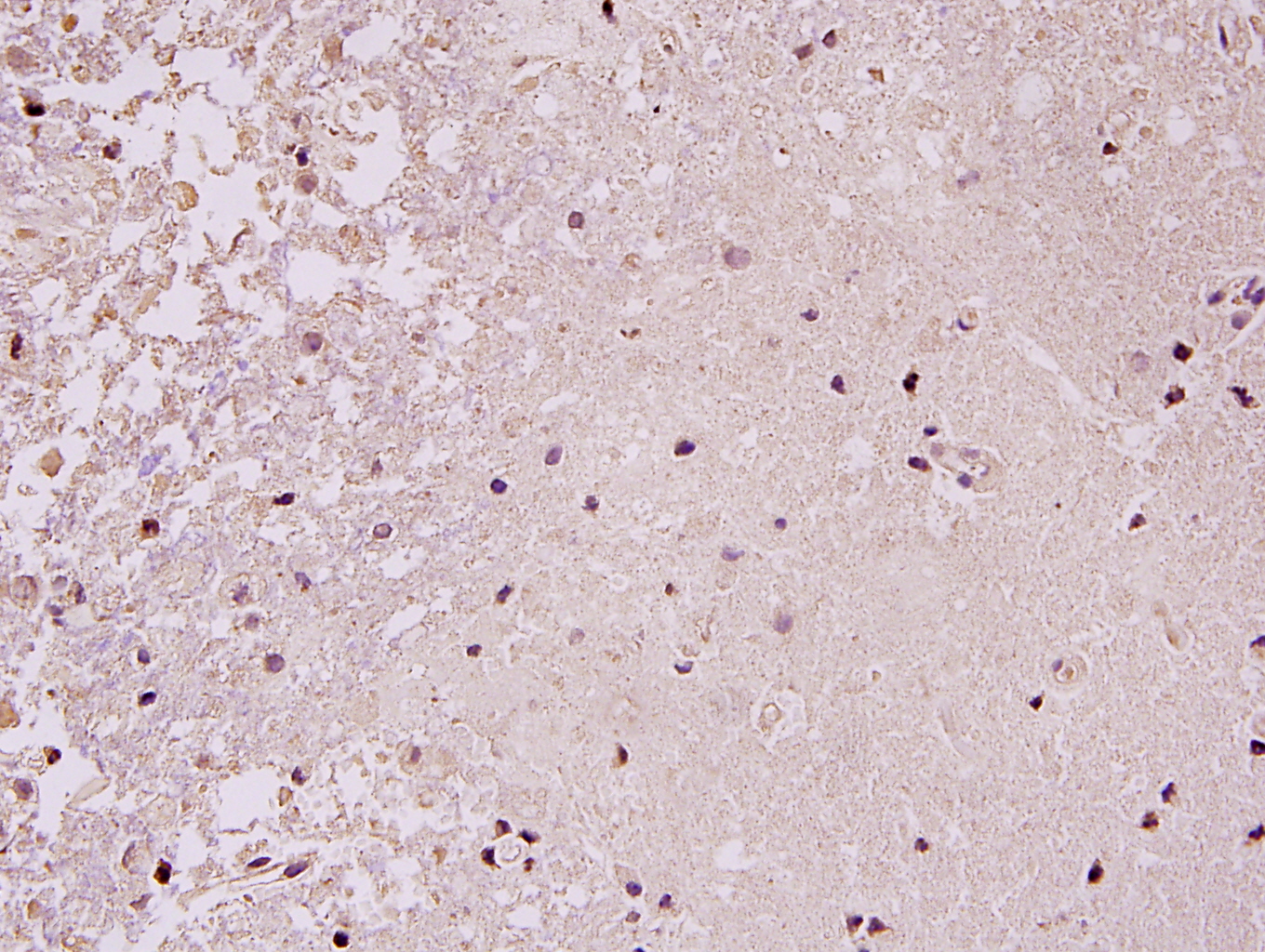

Supplement: S1 File — (ZIP) [file pone.0272499.s001.zip › supporting information/immunohistochemical/miR-31antagomirMMP2/5days/2.jpg]

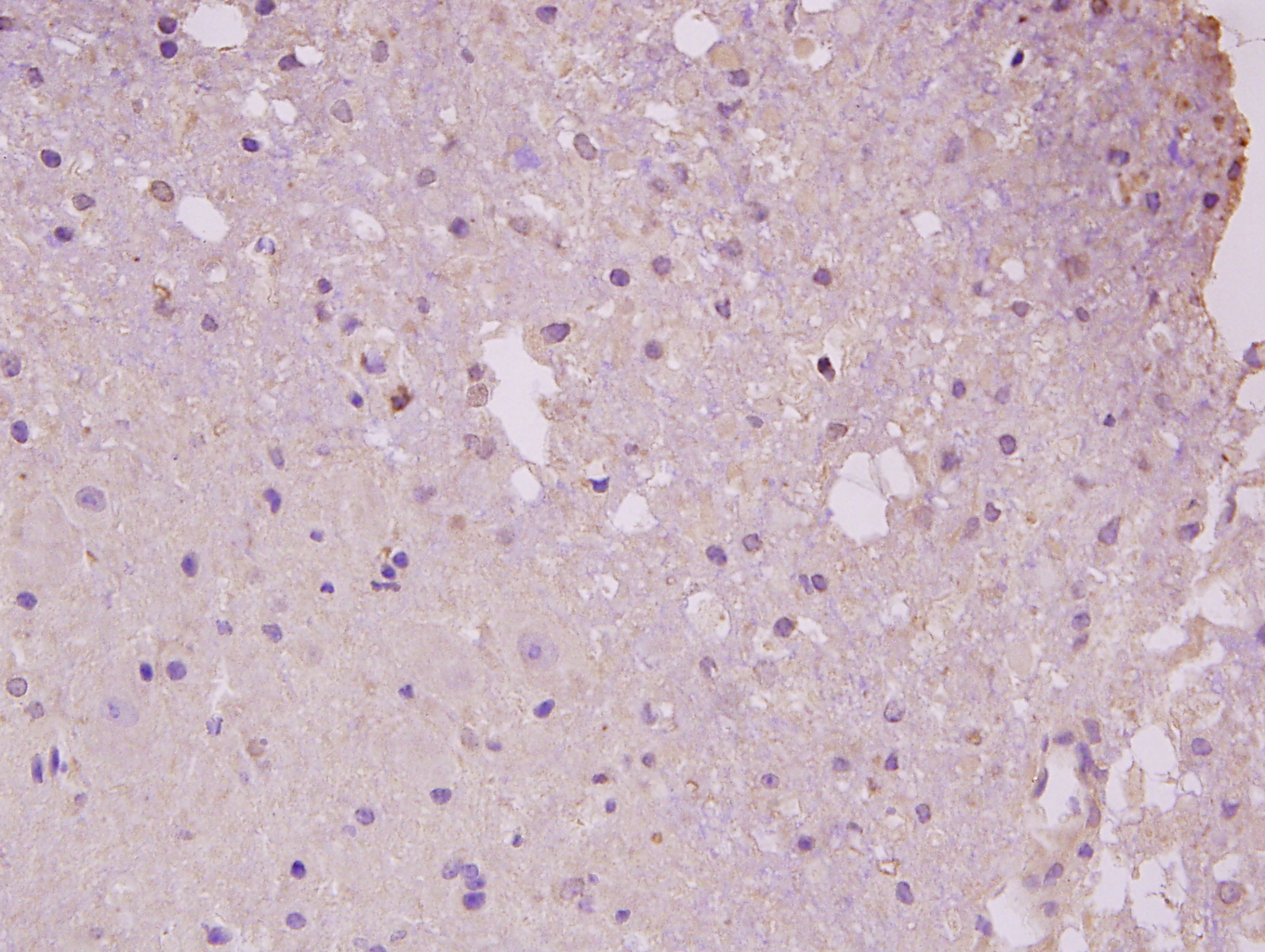

Supplement: S1 File — (ZIP) [file pone.0272499.s001.zip › supporting information/immunohistochemical/miR-31antagomirMMP2/5days/3.jpg]

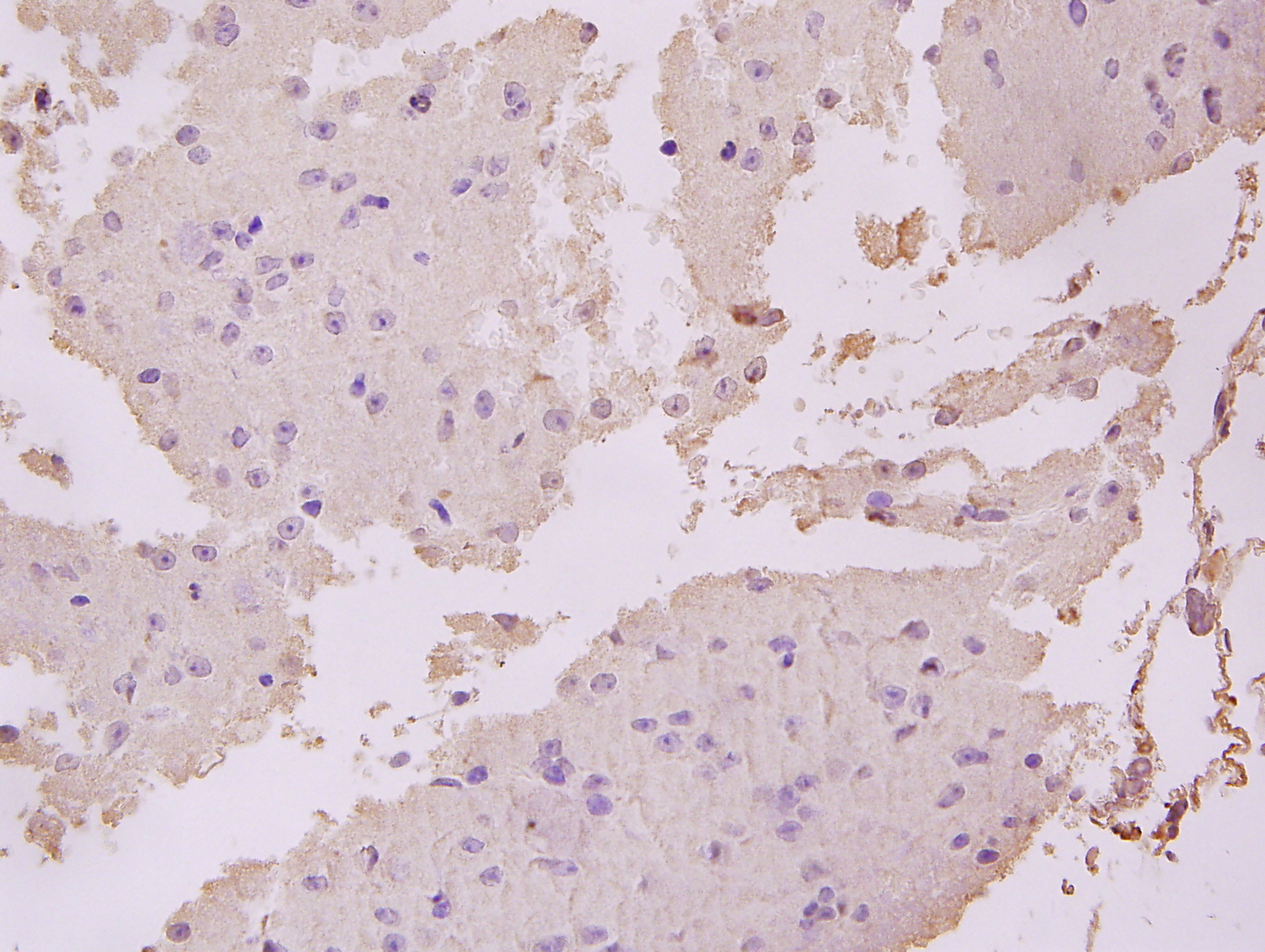

Supplement: S1 File — (ZIP) [file pone.0272499.s001.zip › supporting information/immunohistochemical/miR-31antagomirMMP2/5days/4.jpg]

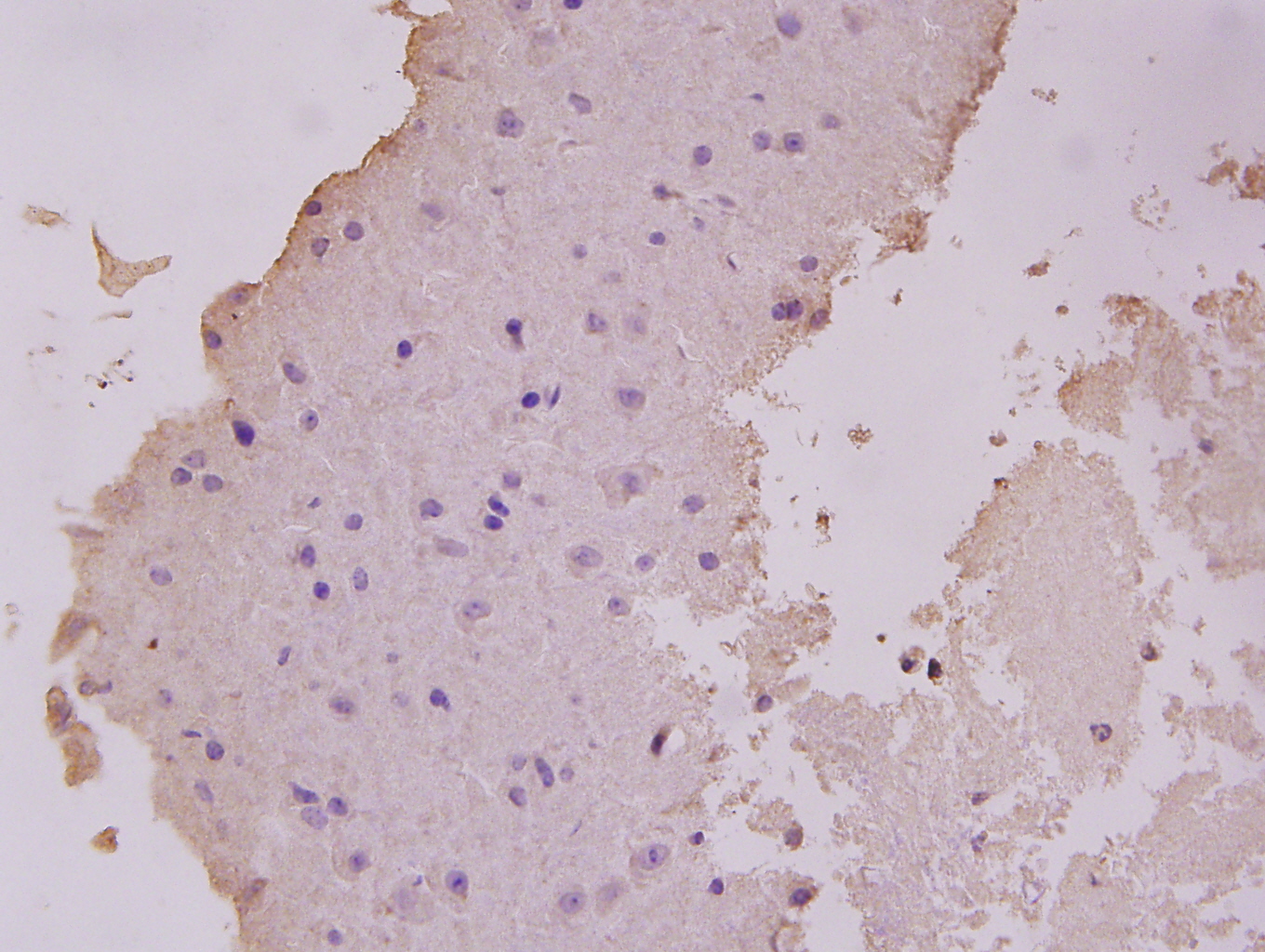

Supplement: S1 File — (ZIP) [file pone.0272499.s001.zip › supporting information/immunohistochemical/miR-31antagomirMMP2/5days/5.jpg]

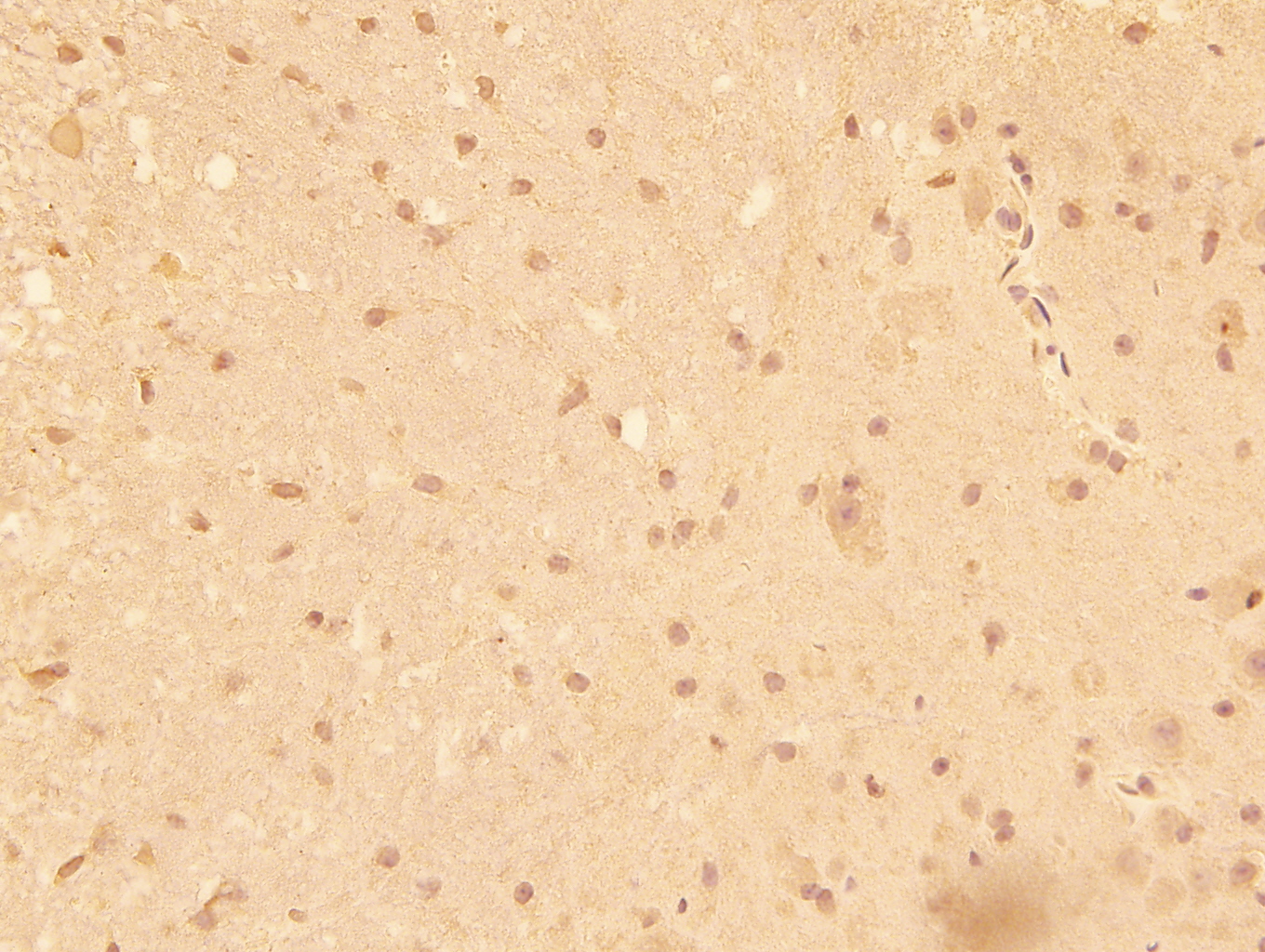

Supplement: S1 File — (ZIP) [file pone.0272499.s001.zip › supporting information/immunohistochemical/miR-31antagomirMMP2/7days/1.jpg]

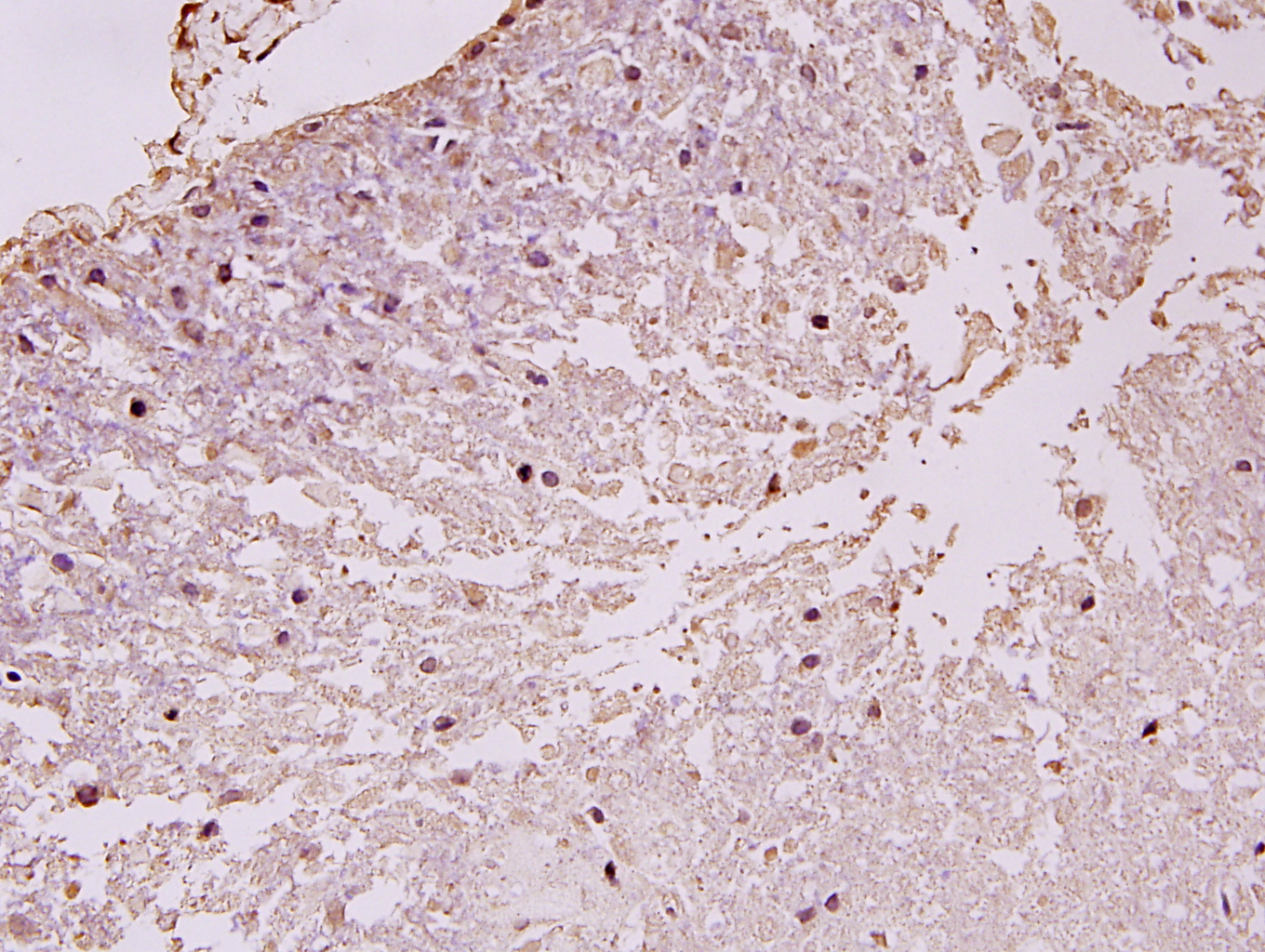

Supplement: S1 File — (ZIP) [file pone.0272499.s001.zip › supporting information/immunohistochemical/miR-31antagomirMMP2/7days/2.jpg]

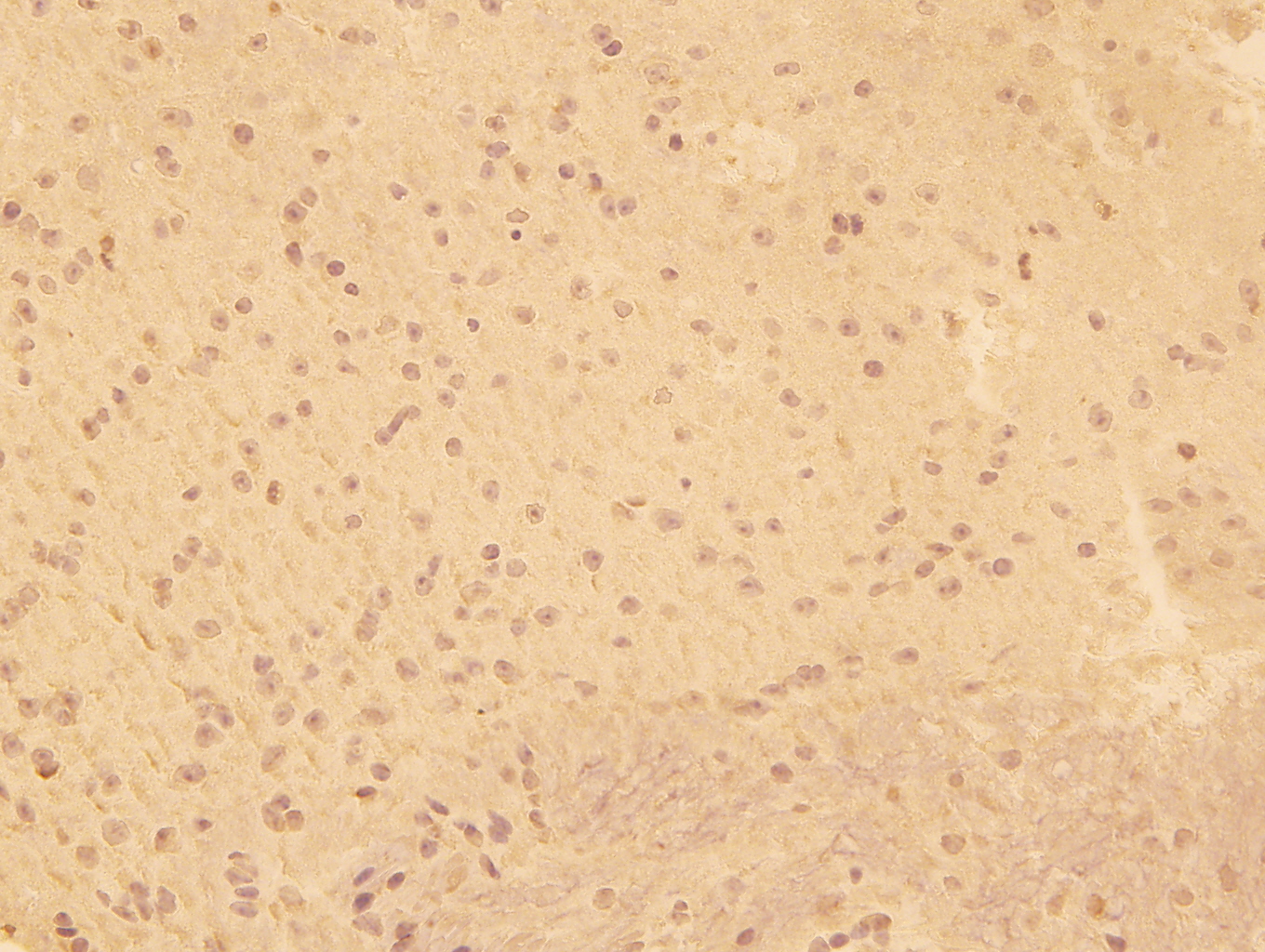

Supplement: S1 File — (ZIP) [file pone.0272499.s001.zip › supporting information/immunohistochemical/miR-31antagomirMMP2/7days/3.jpg]

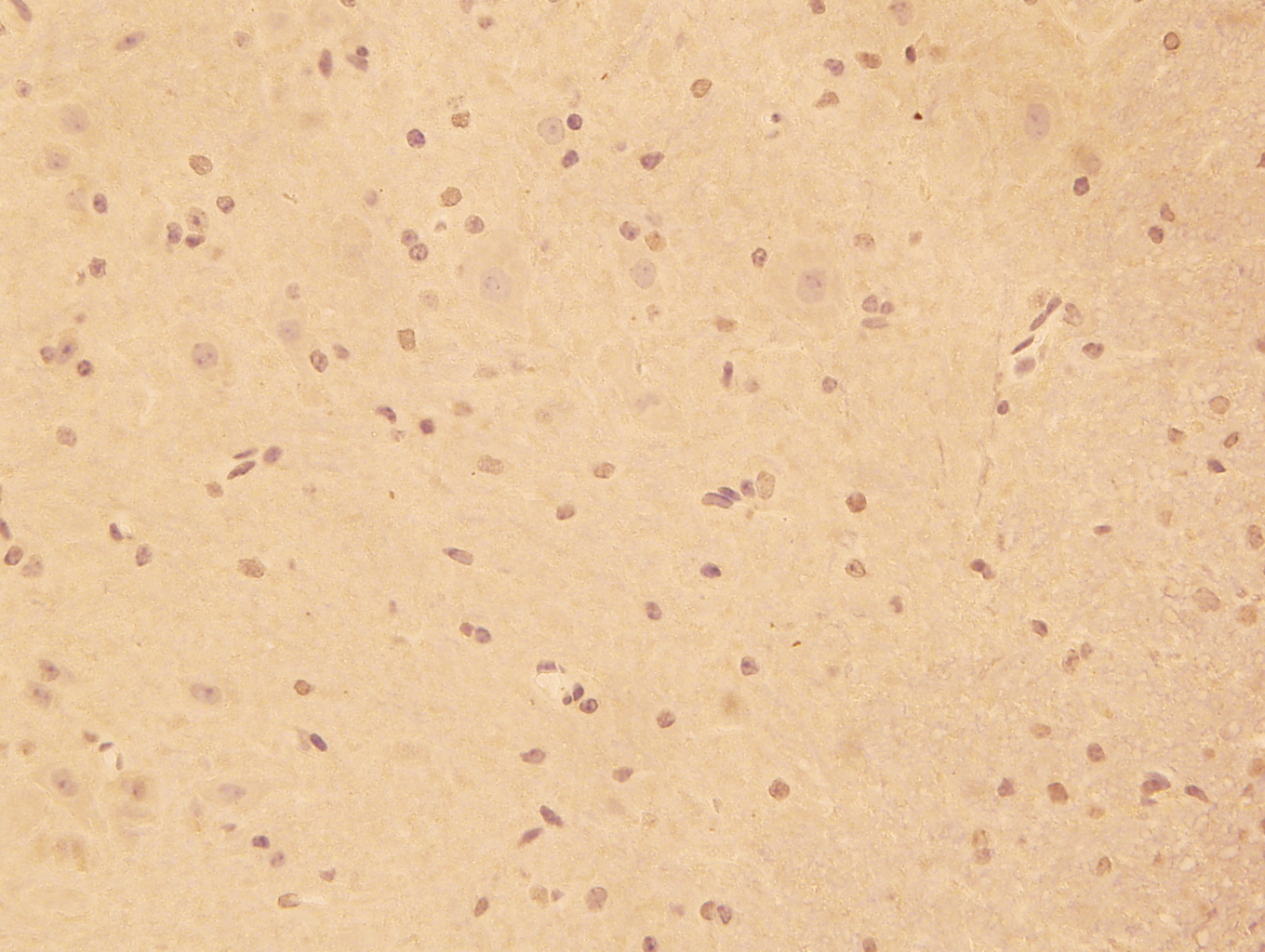

Supplement: S1 File — (ZIP) [file pone.0272499.s001.zip › supporting information/immunohistochemical/miR-31antagomirMMP2/7days/4.jpg]

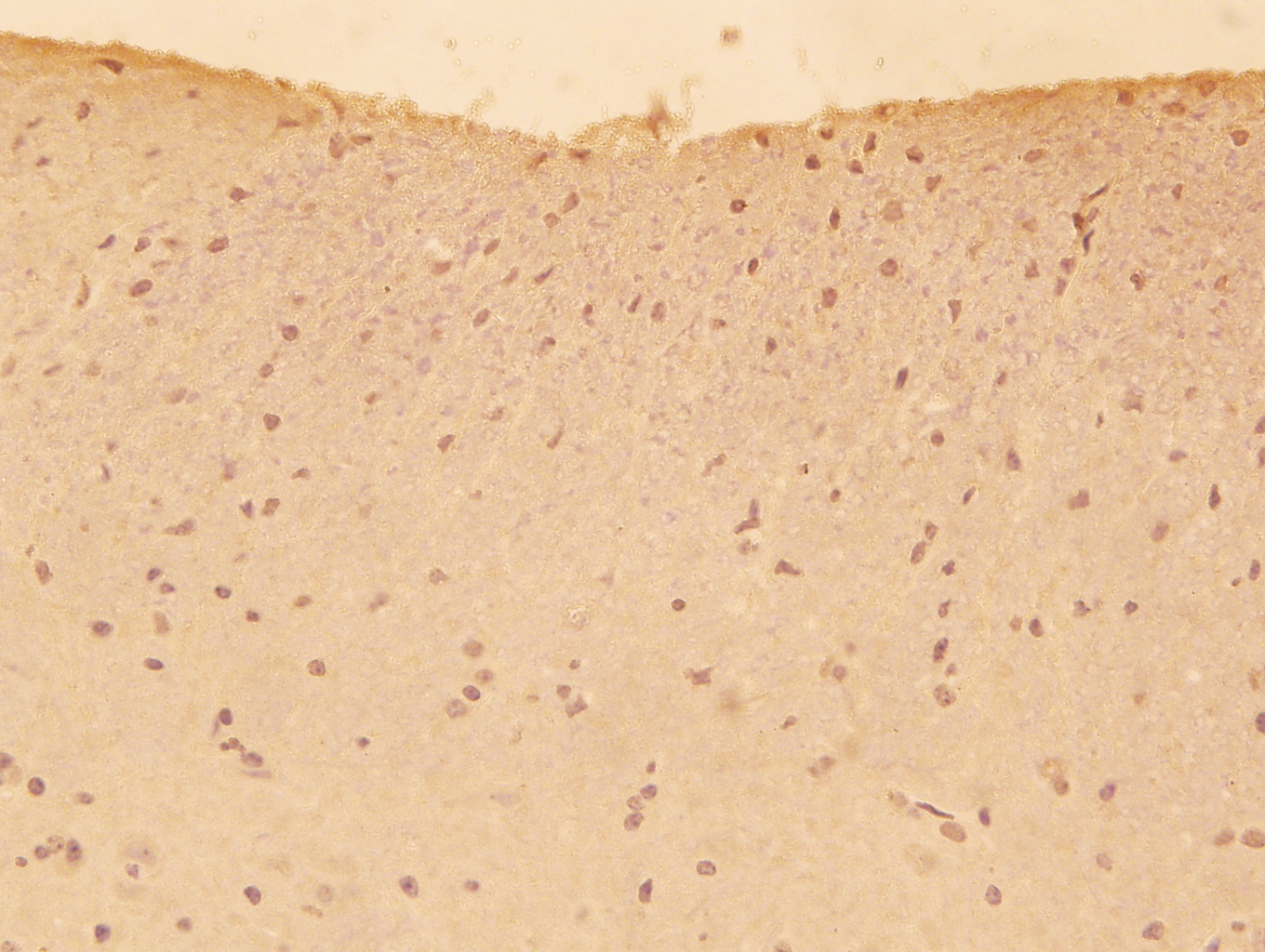

Supplement: S1 File — (ZIP) [file pone.0272499.s001.zip › supporting information/immunohistochemical/miR-31antagomirMMP2/7days/5.jpg]

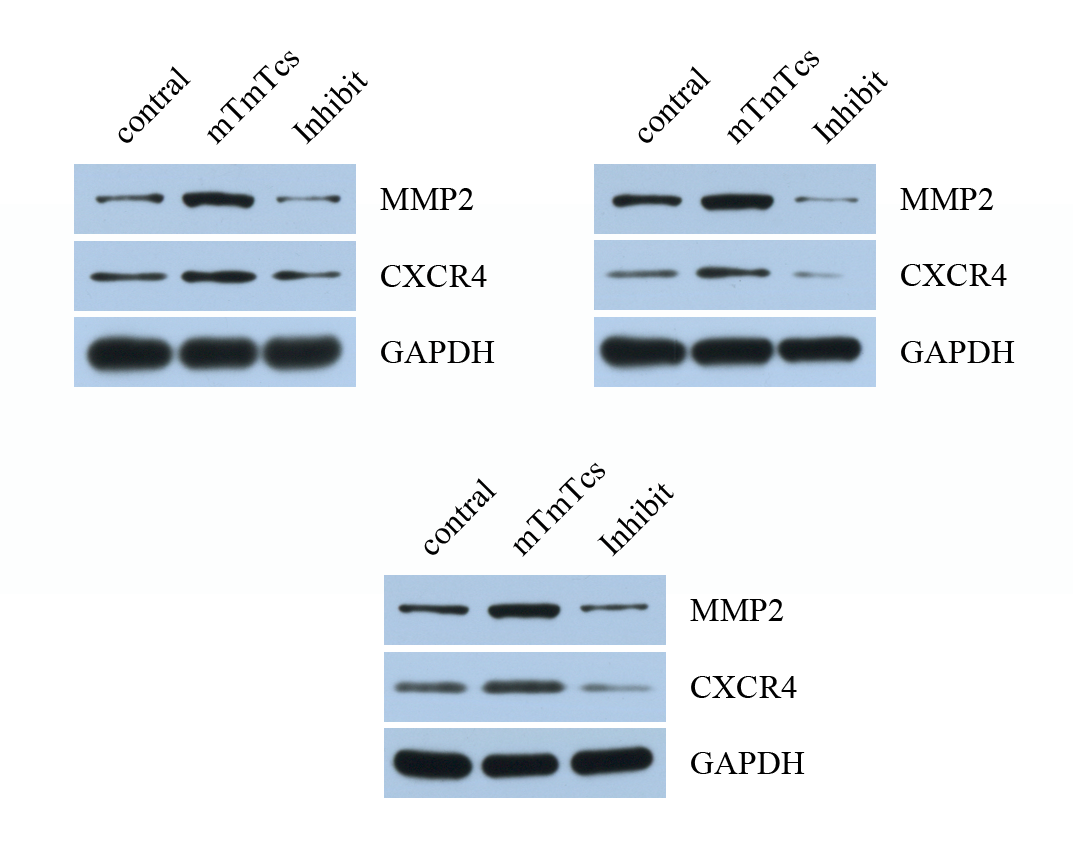

Supplement: S2 File — (ZIP) [file pone.0272499.s002.zip › Supporting Information/Fig5/╒┼╙±╛Ω ╒√└φ.tif]

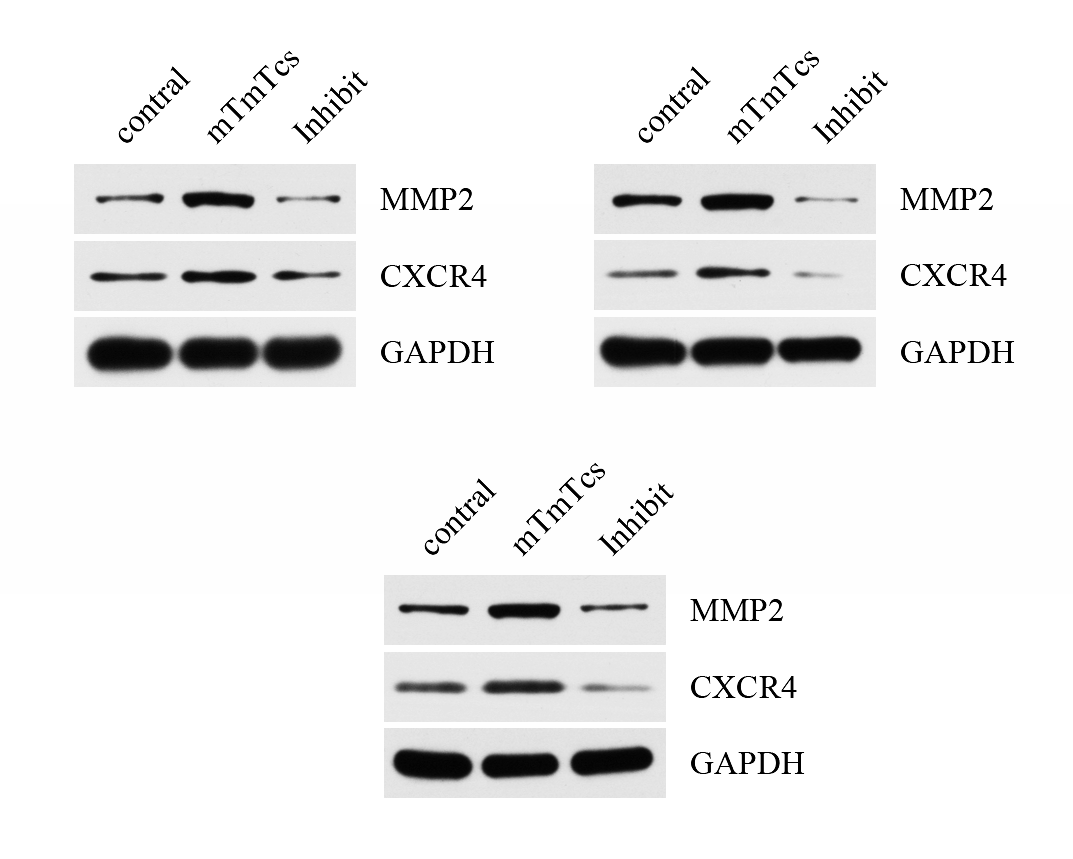

Supplement: S2 File — (ZIP) [file pone.0272499.s002.zip › Supporting Information/Fig5/╒┼╙±╛Ω ╒√└φ╗╥.tif]

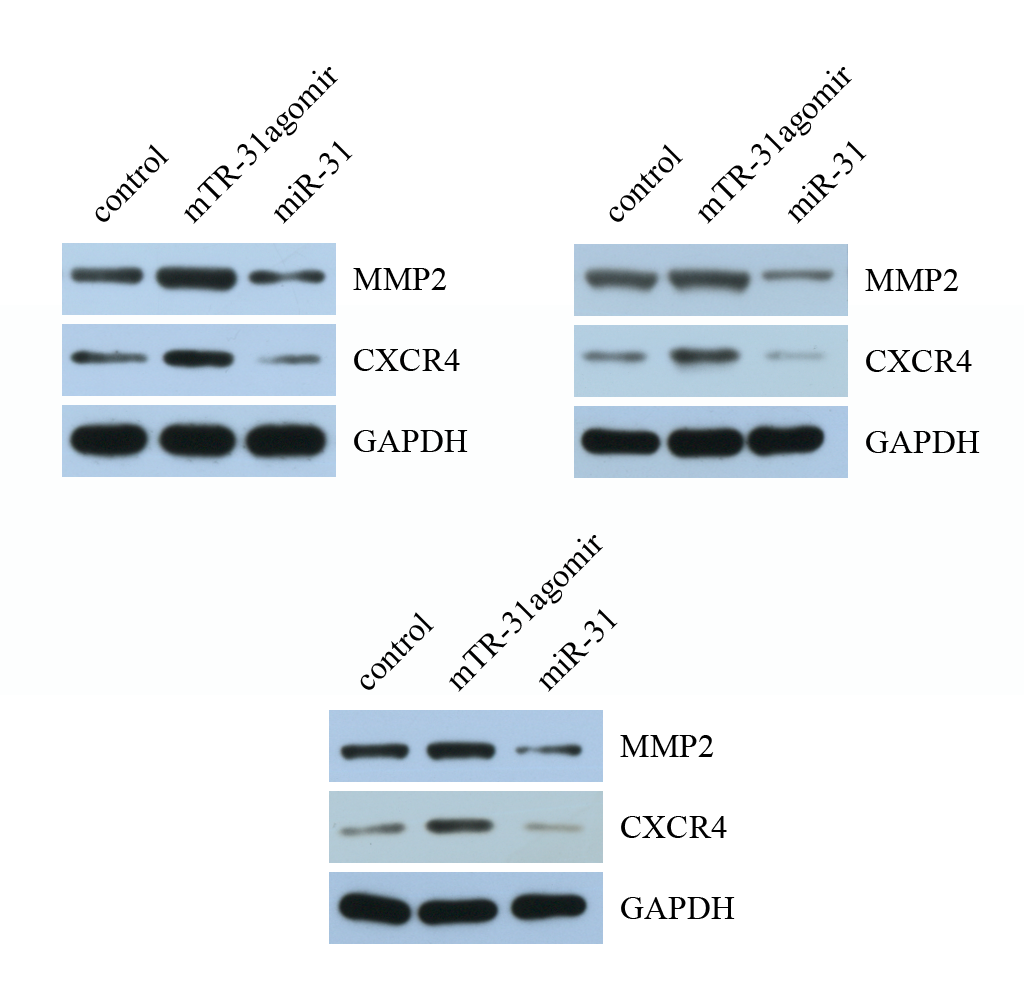

Supplement: S2 File — (ZIP) [file pone.0272499.s002.zip › Supporting Information/Fig7/╒┼╙±╛Ω ╒√└φ.tif]

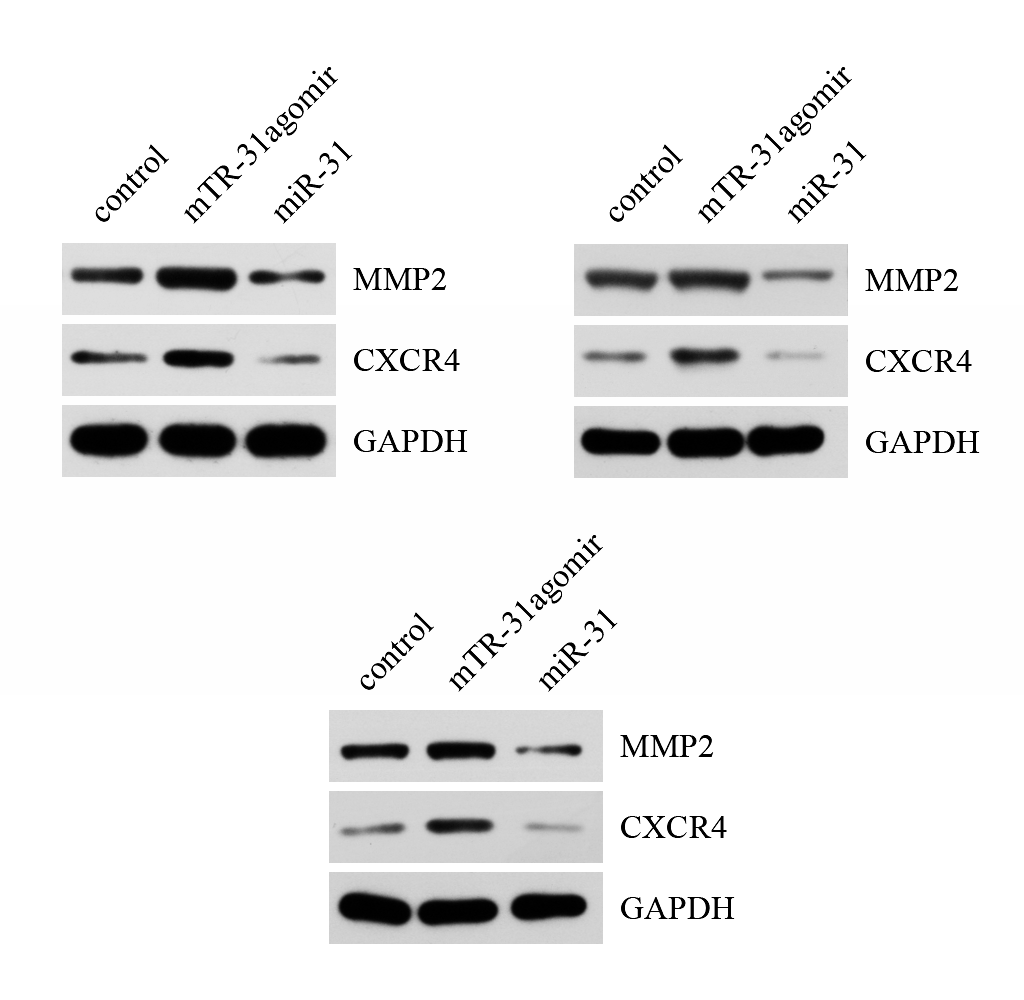

Supplement: S2 File — (ZIP) [file pone.0272499.s002.zip › Supporting Information/Fig7/╒┼╙±╛Ω ╒√└φ╗╥.tif]
