# Supplementary material for: MiR-31 improves spinal cord injury in mice by promoting the migration of bone marrow mesenchymal stem cells
Source: PLoS One. 2022 Sep 6;17(9):e0272499. doi: 10.1371/journal.pone.0272499 (PMC9447891; doi:10.1371/journal.pone.0272499)
Supplement: S2 File — (ZIP) [file pone.0272499.s002.zip › Supporting Information/S1_raw_images.pdf]

Raw blot and gel images of Figure 5

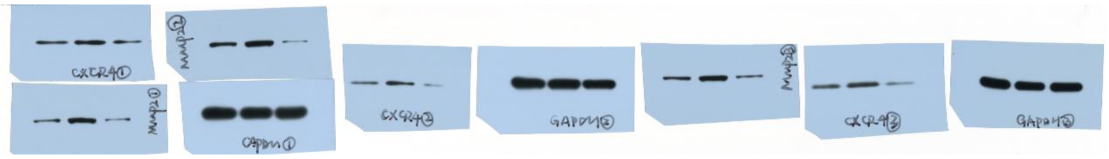

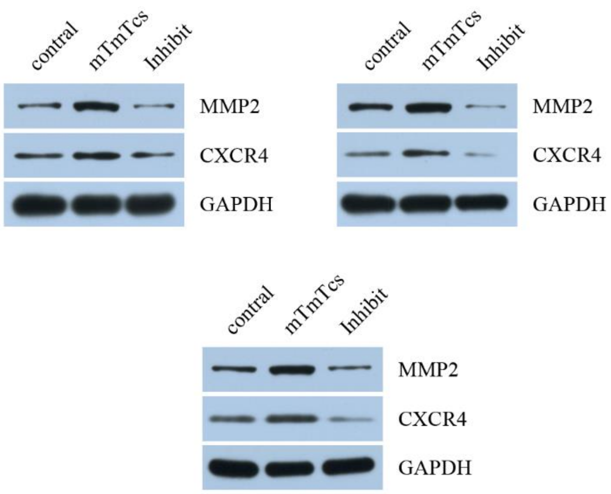

Three diagrams, the first row indicates the 1st, 2nd and these two are not used represented by X. The second row indicates the 3rd one.

The molecular sizes are 55kD,39kD,37kD from top to bottom.

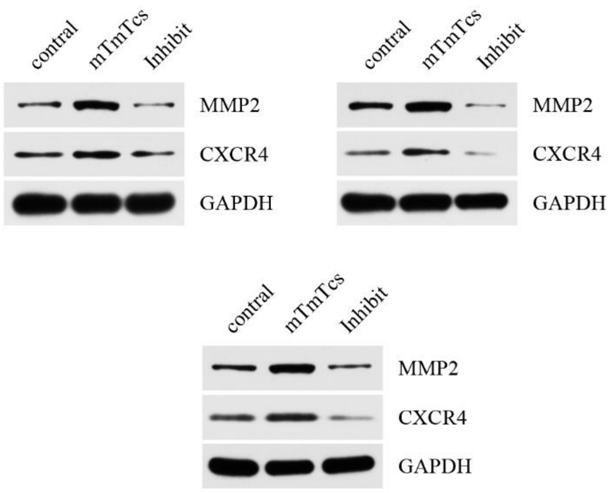

Three diagrams, the first row indicates the 1st, 2nd and these two are not used represented by X. The second row indicates the 3rd one.

The molecular sizes are 55kD,39kD,37kD from top to bottom.

The order of sample addition is from left to right: control group, miR-

31agomir group and miR-31antagomir group.

The experimental samples for the cell experiments were bone marrow mesenchymal stem cells, proteins from bone marrow mesenchymal stem cells transfected with miR-31agomir and miR-31antagomir. The experiment was repeated three times.

The original image was obtained by developing and photographing by the high-sensitivity chemiluminescence imaging system ECL, and the image was acquired by analyzing the gray value of the image by ImageJ software. The figure panel is generated from the third original image.

Raw blot and gel images of Figure 7

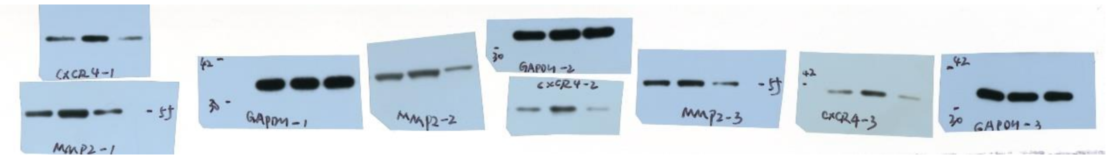

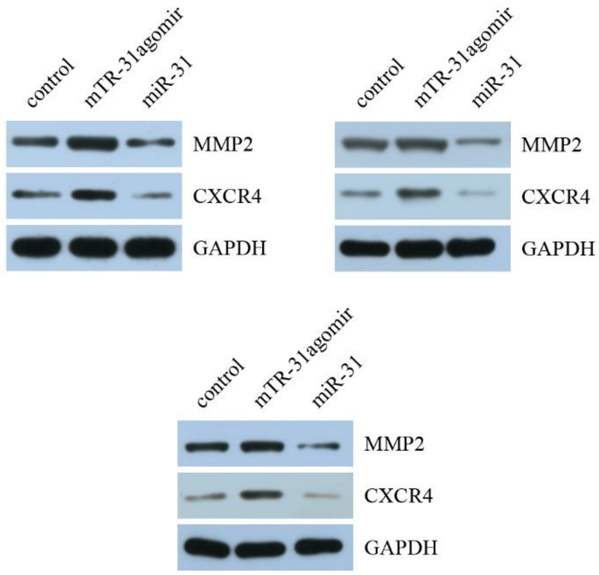

Three diagrams, the first row indicates the 1st, 2nd. The second row indicates the 3rd one. where the second and third are not used are denoted by X. The molecular sizes are 55kD,39kD,37kD from top to bottom

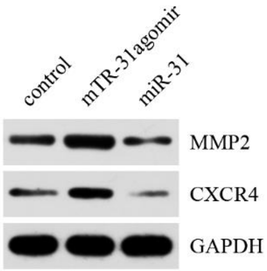

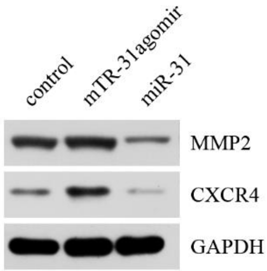

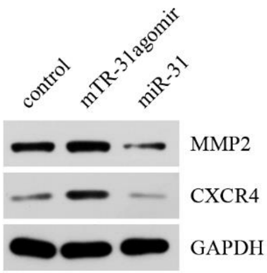

Three diagrams, the first row indicates the 1st, 2nd. The second row indicates the 3rd one. where the second and third are not used are denoted by X. The molecular sizes are 55kD,39kD,37kD from top to bottom

The order of sample addition is from left to right: control group, miR-31agomir group and miR-31antagomir group.

The experimental samples for animal experiments were bone marrow mesenchymal stem cells, pretreated miR-31agomir and miR-31antagomir of bone marrow mesenchymal stem cells transplanted with proteins from the site of spinal cord injury after treatment. The experiment was repeated three times.

The original image was obtained by developing and photographing by the high-sensitivity chemiluminescence imaging system ECL, and the image was acquired by analyzing the gray value of the image by ImageJ software. The figure panel is generated from the first original image.
