# Supplementary material for: MiR-31 improves spinal cord injury in mice by promoting the migration of bone marrow mesenchymal stem cells
Source: PLoS One. 2022 Sep 6;17(9):e0272499. doi: 10.1371/journal.pone.0272499 (PMC9447891; doi:10.1371/journal.pone.0272499)

# PLOS ONE

## MiR-31 improves spinal cord injury in mice by promoting the migration of bone marrow mesenchymal stem cells

--Manuscript Draft--

| | |
|---|---|
| **Manuscript Number:** | PONE-D-21-40503 |
| **Article Type:** | Research Article |
| **Full Title:** | MiR-31 improves spinal cord injury in mice by promoting the migration of bone marrow mesenchymal stem cells |
| **Short Title:** | miR-31 promotes migration of bone marrow mesenchymal stem cells |
| **Corresponding Author:** | Chunfang Wang<br>Shanxi Medical University<br>Taiyuan, CHINA |
| **Keywords:** | Bone marrow mesenchymal stem cells (BMSCs); MicroRNAs; MiR-31; Cell migration; Spinal cord injury (SCI) |
| **Abstract:** | Background: Stem cell transplantation therapy is a potential approach for the repair of spinal cord injuries and other neurodegenerative diseases, but the effectiveness of cell transplantation is hampered by the low rate of targeted migration of cells to the area of injury. The aim of this study was to investigate the effects of miR-31 on the migration of bone marrow mesenchymal stem cells and the regulation of MMP-2 and CXCR4 expression in vitro and in vivo. Methods: Culture and identification of eGFP BMSCs. The experiments were divided into control group, miR-31agomir group and miR-31antagomir group. Proliferation was analyzed using CCK-8 and flow cytometry; cell migration in vitro was analyzed using Wound-healing and Transwell assays. The mouse spinal cord injury model was prepared by the impact method, and cells were transplanted (3 groups, n=12/group). Detection of relevant inflammatory factors by ELISA. The BMS score was used to evaluate the functional recovery of the mouse spinal cord; the frozen section was used to analyze the cell migration ability in vivo. The expression of MMP-2 and CXCR4 was evaluated in vitro and in vivo by Western blot and immunohistochemical staining, respectively. Results : In vitro experiments showed that the miR-31agomir group had enhanced cell proliferation (P<0.05, P<0.001), promoted cell migration(P <0.001), and upregulated CXCR4 and MMP-2 protein expression (P<0.01, P <0.001) compared with the control group. The results of in vivo experiments showed that the expression of pro-inflammatory factors was reduced after cell transplantation treatment. The miR-31agomir group had enhanced cell-targeted migration ability (P<0.001), improved the function of damaged tissues (P<0.001), and upregulated CXCR4 and MMP-2 expression compared to the control group (P<0.001). Conclusion : The experiment demonstrated that miR-31 can promote the migration of bone marrow mesenchymal stem cells and that miR-31 can repair and improve the function of damaged tissues in spinal cord injury. |
| **Order of Authors:** | Yujuan Zhang |
| | Lili Cao |
| | Ruochen Du |
| | Feng Tian |
| | Xiao Li |
| | Yitong Yuan |
| | Chunfang Wang |
| **Additional Information:** | |
| **Question** | **Response** |
| **Financial Disclosure**<br><br>Enter a financial disclosure statement that | The author(s) received no specific funding for this work. |

Powered by Editorial Manager® and ProduXion Manager® from Aries Systems Corporation

| | |
|---|---|
| describes the sources of funding for the work included in this submission. Review the submission guidelines for detailed requirements. View published research articles from *PLOS ONE* for specific examples.<br><br>This statement is required for submission and **will appear in the published article** if the submission is accepted. Please make sure it is accurate.<br><br>**Unfunded studies**<br>Enter: *The author(s) received no specific funding for this work.*<br><br>**Funded studies**<br>Enter a statement with the following details:<br>• Initials of the authors who received each award<br>• Grant numbers awarded to each author<br>• The full name of each funder<br>• URL of each funder website<br>• Did the sponsors or funders play any role in the study design, data collection and analysis, decision to publish, or preparation of the manuscript?<br>• **NO** - Include this sentence at the end of your statement: *The funders had no role in study design, data collection and analysis, decision to publish, or preparation of the manuscript.*<br>• **YES** - Specify the role(s) played.<br><br>* typeset | |
| **Competing Interests**<br><br>Use the instructions below to enter a competing interest statement for this submission. On behalf of all authors, disclose any competing interests that could be perceived to bias this work—acknowledging all financial support and any other relevant financial or non-financial competing interests.<br><br>This statement is required for submission and **will appear in the published article** if the submission is accepted. Please make sure it is accurate and that any funding | The authors declare that they have no competing interests. |

| | |
|---|---|
| sources listed in your Funding Information later in the submission form are also declared in your Financial Disclosure statement.<br><br>View published research articles from *PLOS ONE* for specific examples.<br><br>**NO authors have competing interests**<br><br>Enter: *The authors have declared that no competing interests exist.*<br><br>**Authors with competing interests**<br><br>Enter competing interest details beginning with this statement:<br><br>*I have read the journal's policy and the authors of this manuscript have the following competing interests: [insert competing interests here]*<br><br>* typeset | |
| **Ethics Statement**<br><br>Enter an ethics statement for this submission. This statement is required if the study involved:<br><br>• Human participants<br>• Human specimens or tissue<br>• Vertebrate animals or cephalopods<br>• Vertebrate embryos or tissues<br>• Field research<br><br>Write "N/A" if the submission does not require an ethics statement.<br><br>General guidance is provided below. Consult the submission guidelines for detailed instructions. **Make sure that all information entered here is included in the Methods section of the manuscript.** | All experimental procedures involving animals were approved by the Ethical Research Committee of the Laboratory Animal Center of Shanxi Medical University (Approval No. SYDL2021014) and the Regulations of the People's Republic of China on the Administration of Laboratory Animals. |

Powered by Editorial Manager® and ProduXion Manager® from Aries Systems Corporation

| | |
|---|---|
| **Format for specific study types**<br><br>**Human Subject Research (involving human participants and/or tissue)**<br>• Give the name of the institutional review board or ethics committee that approved the study<br>• Include the approval number and/or a statement indicating approval of this research<br>• Indicate the form of consent obtained (written/oral) or the reason that consent was not obtained (e.g. the data were analyzed anonymously)<br><br>**Animal Research (involving vertebrate animals, embryos or tissues)**<br>• Provide the name of the Institutional Animal Care and Use Committee (IACUC) or other relevant ethics board that reviewed the study protocol, and indicate whether they approved this research or granted a formal waiver of ethical approval<br>• Include an approval number if one was obtained<br>• If the study involved *non-human primates*, add *additional details* about animal welfare and steps taken to ameliorate suffering<br>• If anesthesia, euthanasia, or any kind of animal sacrifice is part of the study, include briefly which substances and/or methods were applied<br><br>**Field Research**<br><br>Include the following details if this study involves the collection of plant, animal, or other materials from a natural setting:<br>• Field permit number<br>• Name of the institution or relevant body that granted permission | |
| **Data Availability**<br><br>Authors are required to make all data underlying the findings described fully available, without restriction, and from the time of publication. PLOS allows rare exceptions to address legal and ethical concerns. See the PLOS Data Policy and FAQ for detailed information. | Yes - all data are fully available without restriction |

Powered by Editorial Manager® and ProduXion Manager® from Aries Systems Corporation

| | |
|---|---|
| A Data Availability Statement describing where the data can be found is required at submission. Your answers to this question constitute the Data Availability Statement and **will be published in the article**, if accepted.<br><br>**Important:** Stating 'data available on request from the author' is not sufficient. If your data are only available upon request, select 'No' for the first question and explain your exceptional situation in the text box.<br><br>Do the authors confirm that all data underlying the findings described in their manuscript are fully available without restriction? | |
| **Describe where the data may be found in full sentences. If you are copying our sample text, replace any instances of XXX with the appropriate details.**<br><br>• If the data are **held or will be held in a public repository**, include URLs, accession numbers or DOIs. If this information will only be available after acceptance, indicate this by ticking the box below. For example: *All XXX files are available from the XXX database (accession number(s) XXX, XXX.*).<br>• If the data are all contained **within the manuscript and/or Supporting Information files**, enter the following: *All relevant data are within the manuscript and its Supporting Information files.*<br>• If neither of these applies but you are able to provide **details of access elsewhere**, with or without limitations, please do so. For example:<br><br>*Data cannot be shared publicly because of [XXX]. Data are available from the XXX Institutional Data Access / Ethics Committee (contact via XXX) for researchers who meet the criteria for access to confidential data.*<br><br>*The data underlying the results presented in the study are available from (include the name of the third party* | All relevant data are within the manuscript and its Supporting Information files. |

| | |
|---|---|
| *and contact information or URL).*<br>• This text is appropriate if the data are owned by a third party and authors do not have permission to share the data.<br><br>* typeset | |
| Additional data availability information: | |

Manuscript Click here to access/download;Manuscript;manuscript.docx

# MiR-31 improves spinal cord injury in mice by promoting the migration of bone marrow mesenchymal stem cells

Yujuan Zhang[1], Lili Cao[1,2,3], Ruochen Du[1], Feng Tian[2], Xiao Li[1], Yitong Yuan[1*], Chunfang Wang[1*]

[1]Laboratory Animal Research Center of Shanxi Medical University, Taiyuan, Shanxi,China

[2]Shanxi Province Key Laboratory of Oral Diseases Prevention and New Materials, Taiyuan, Shanxi,China

[3]School and Hospital of Stomatology, Shanxi Medical University, Taiyuan, Shanxi,China

[*]Corresponding authors:

Email: wangchunfang@sxmu.edu.cn (WCF)

Email: ytyuan@sxmu.edu.cn (YYT)

## Abstract

**Background:** Stem cell transplantation therapy is a potential approach for the repair of spinal cord injuries and other neurodegenerative diseases, but the effectiveness of cell transplantation is hampered by the low rate of targeted migration of cells to the area of injury. The aim of this study was to investigate the effects of miR-31 on the migration of bone marrow mesenchymal stem cells and the regulation of MMP-2 and CXCR4 expression in vitro and in vivo. **Methods:** Culture and identification of eGFP BMSCs. The experiments were divided into control group, miR-31agomir group and miR-

31antagomir group. Proliferation was analyzed using CCK-8 and flow cytometry; cell migration in vitro was analyzed using Wound-healing and Transwell assays. The mouse spinal cord injury model was prepared by the impact method, and cells were transplanted (3 groups, n=12/group). Detection of relevant inflammatory factors by ELISA. The BMS score was used to evaluate the functional recovery of the mouse spinal cord; the frozen section was used to analyze the cell migration ability in vivo. The expression of MMP-2 and CXCR4 was evaluated in vitro and in vivo by Western blot and immunohistochemical staining, respectively.**Results**：In vitro experiments showed that the miR-31agomir group had enhanced cell proliferation (P<0.05, P<0.001), promoted cell migration(P<0.001), and upregulated CXCR4 and MMP-2 protein expression (P<0.01, P<0.001) compared with the control group. The results of in vivo experiments showed that the expression of pro-inflammatory factors was reduced after cell transplantation treatment. The miR-31agomir group had enhanced cell-targeted migration ability (P<0.001), improved the function of damaged tissues (P<0.001), and upregulated CXCR4 and MMP-2 expression compared to the control group (P<0.001).**Conclusion**：The experiment demonstrated that miR-31 can promote the migration of bone marrow mesenchymal stem cells and that miR-31 can repair and improve the function of damaged tissues in spinal cord injury.

**Key words:** Bone marrow mesenchymal stem cells (BMSCs); MicroRNAs; MiR-31; Cell migration; Spinal cord injury (SCI)

# Introduction

Spinal cord injury (SCI) is a serious and devastating neurodegenerative disease characterized by chronic pain, sudden loss of motor sensation and associated oxidative stress and axonal degeneration, ultimately leading to lifelong failure(1, 2). Pro-inflammatory cytokines can make spinal cord injury neurological impairment more severe, so suppression of inflammation is another important principle in the treatment of spinal cord injury(3). Globally, spinal cord injuries have a high morbidity and mortality rate and result in serious economic and social problems(4). Therefore, the treatment of spinal cord injury has become a focus of scientific and medical research, which has led to methods such as drug therapy, surgery and cell therapy.(5). However, with the development of tissue engineering in recent years, stem cell transplantation is considered the most promising method, especially bone marrow mesenchymal stem cells (BMSCs)(6, 7). BMSCs are stem cells with self-renewal and multidirectional differentiation as well as immunomodulatory properties(8). At the same time, mesenchymal stem cells are very sensitive to changes in inflammatory conditions, and the inflammatory environment will regulate the anti-inflammatory potential of BMSCs(9). In spinal cord injury, BMSCs are able to migrate to the region of injury to improve behavioral responses and differentiate into neurons for functional repair due to their low immunogenicity, targeted homing, and secretion of trophic factors that enhance neuronal survival. However, the limited ability to migrate to the area of injury limits the effectiveness and application of cell transplantation therapy(8, 10, 11).

MicroRNAs (miRNAs) are a new form of gene regulation, a conserved endogenous class of small, single-stranded, non-coding RNAs of approximately 22 nucleotides that inhibit the translation or stability of mRNAs by interacting with the 3'-untranslated (3'-UTR) regions of target genes and are involved in several biological processes(12, 13). In recent years, studies have found that miRNAs play an important role in regulating the apoptosis, proliferation, migration, and differentiation of bone marrow mesenchymal stem cells(13). MicroRNA-31 (miR-31) is a type of miRNAs, which plays an important regulatory role in biological processes such as cell proliferation and migration(14). In cancer, miR-31 is an important regulator, often acting as an oncogene involved in the proliferation, migration and invasion of cancer cells(15). However, there are multiple overlapping mechanisms in the characteristics of stem cells and cancer cells(16). This research group mainly studies the role of miR-31 in stem cells. It has been previously studied that miR-31 promotes the differentiation of spinal cord-derived neural stem cells into neurons and that miR-31 has embryonic specificity in regulating the proliferation of neural stem cells (17)。However, the effect of miR-31 on the migration of bone marrow mesenchymal stem cells is not clear. Therefore, it is important to investigate whether miR-31 has a facilitative effect on the migration of bone marrow MSCs to repair damaged tissues.

# Materials and methods

## Animals

The Green fluorescent protein (e GFP) transgenic mice (6-8 weeks,15-20g) are the previous successful tool mice constructed by our group. Female FVB mice (8-10 weeks, 18-25 g) for spinal cord injury modeling were purchased from Spelford Biotechnology Ltd (Beijing China). The entire experiment was conducted in a barrier environment. All mice were raised under standard conditions (temperature 23-26℃, humidity 50%-60%, normal 12h light/12h dark biorhythm) and were free to take water and food. All methods and operations of this experiment were in accordance with the regulations for the husbandry and use of laboratory animals at Shanxi Medical University, and were conducted under the approval of the Ethics Committee for Laboratory Animals of Shanxi Medical University (Approval No.: SYDL2021014).

## Isolation, culture and identification of BMSCs

BMSCs from e GFP mice were extracted using the whole bone marrow apposition method. The e GFP mice were anesthetized intraperitoneally with 3% pentobarbital(30mg/kg). Dissect bilateral femurs and tibias, remove surrounding tissues and muscles, and rinse with phosphate- buffered saline (PBS,Solarbio,China) 3 times. Cut both ends of the bone with tissue scissors, rinse the bone marrow cavity three times with a 1 mL syringe, aspirate the medium, collect the buffer, centrifuge and discard the supernatant, and mix the precipitate with the prepared DME/F-12(Gibco,US). A single

cell suspension is formed and transferred to a T25 culture flask (Corning,US). Incubate at 37°C for 4h in a 5% $CO_2$ cell incubator and then change the solution. The fluid was changed once every three days. Cells can be passaged when the degree of fusion reaches 80%, and all experiments use P3 to P5. BMSCs were inoculated on six-well plates (Corning,US) and the expression of their important markers CD90, CD34 and CD44(BOSTER,China) were identified by immunofluorescence techniques.

## Cell transfection

P3-P5 BMSCs were inoculated into a six-well plate ($1\times10^5$ cells/well) containing fetal bovine serum (FBS,Gibco,US) medium. When the cells reach 30%-50% confluency, inoculate the mixture of miR-31agomir (RIBBIO Guangzhou, China) and transfection reagent (Polyplus, France) and the mixture of miR-31 antagomir (RIBBIO Guangzhou, China) and transfection reagent on a six-well plate and culture for 12 hours. After 12h, switch to normal medium for incubation. This resulted in miR-31agomir final concentrations of 50μM and miR-31 antagomir final concentrations of 100μM.

## Transfection efficiency analysis

Since our customized miR-31 siRNA drug is modified by Cy3 fluorescent labeling, the transfection can be directly observed under a fluorescence microscope. After 24h of transfection, the medium was first discarded and washed with PBS for 3 washes, then the cells were fixed with 4% paraformaldehyde (Solarbio,China) and the nuclei were stained with HOCHEST(Germany) and the experiment was repeated 3 times. Finally ,

the images are collected for analysis.

## Real-time fluorescence quantitative PCR（RT-qPCR）

Total RNA was first extracted using TRIZOL reagent (TAKARA, Japan). It was then reverse transcribed into cDNA by the miRNA First-Strand Synthesis Kit (TAKARA, Japan). Finally quantitative analysis was performed on a Real-Time PCR System using the TB Green kit (TAKARA, Japan) to analyze the expression of miR-31 on cells after transfection. The primers required for the experiments are shown in Table 1.

**Table 1 Primer sequence of q RT-PCR**

| Primer | Sequence (5′-3′) |
|---|---|
| miR-31 | AGGCAAGATGCTGGCATAGCTG |
| U6 Forward | GGAACGATACAGAGAAGATTAGC |
| U6 Reverse | TGGAACGCTTCACGAATTTGCG |

## Cell proliferation analysis

Analysis of miR-31 on the proliferation of BMSCs using cell counting kit-8 (CCK-8) (Solarbio,China). BMSCs were inoculated in 96-well plates (1.0×104cells/well) and transfected when cell fusion reached 30%-50%. 10μL of CCK-8 solution was added to each well, incubated for 4h and then the absorbance value at 450 nm was measured using an enzyme marker. The OD values of 1 day, 2 days, and 3 days were measured respectively, and 5 replicate wells were set in each group.

## Cell cycle analysis

Cell cycle detection by flow cytometry. Collect $5\times10^5$ transfected cells, and then perform experimental operations according to the cell cycle kit (Elabscience, China)instructions. After staining with propidium iodide (PI) staining solution, incubate at 2-8°C for 30 minutes in the dark, and then immediately test on the machine.

## Cell migration analysis

Transwell experiment：The transfected cells were resuspended in a serum-free Transwell (pore diameter: 8μm; Corning, USA) with 200uL (1 x 105cells) in the upper chamber and 600uL of complete medium in the lower chamber and incubated for 24h at 37°C in 5% CO2. They were then fixed in 4% paraformaldehyde and stained with 1% crystalline violet (Solarbio, China). Finally, images of five visual eyes were collected for analysis under a microscope and the experiment was repeated three times.

Wound-healing assay：The cells were seeded in a six-well plate, and the cells were transfected, and the cells were 100% confluent overnight. The next day, the cells were scratched vertically on the bottom of the dish with a yellow gun tip, washed slowly with PBS to remove the scratched cells, added to serum-free medium and incubated in a 37°C, 5% CO2 incubator. Photographs were taken under the microscope at 0h,12h,24,48h and images were collected for analysis using Image J. The experiment was repeated three times.

## Establishment of SCI model

The FVB mice were randomly divided into 3 groups with 12 mice in each group. The mice were fasted for 12 hours before the operation, were anesthetized with 3% sodium pentobarbital in the abdominal cavity, fixed on the operating table, exposed the spinal cord, and hit the spinal cord at T8-T10 with Impactor M-Ⅲ(New York University). The spastic tail swinging, the retraction of the lower limbs and the body of the mouse were used as a sign of successful modeling, and the wound was sutured after the operation. To prevent post-operative infection, gentamicin (8mg/Kg) was administered intramuscularly for 3 consecutive days and the mice were manually urinated twice daily until they resumed urinating on their own. Tissue from the injury site was extracted for histological examination on days 1, 3, 5 and 7 after modelling.

## ELISA of IL-6 and IL-17

Blood from mice was collected, centrifuged at room temperature and the serum was collected for the detection of IL-6 and IL-17 levels in the spinal cord tissue of each group of mice. The specific ELISA kits (BOSTER, China) were used and tested according to the instructions.

## Cell transplantation and grouping

After successfully constructing the spinal cord injury model, cells were transplanted at the edge of the mouse injury at a rate of 1uL/min. Groups: control group injected with 3uL of serum-free medium containing BMSCS; miR-31agomir group

injected with 3uL of serum-free medium containing miR-31agomir-transfected BMSCS; miR-31 antagomir group injected with 3uL of serum-free medium containing miR-31 antagomir-transfected BMSCS. Consistent number of cells contained in each group（1.0 $\times 10^5$/μL cells）

## Basso Mouse Scale（BMS）score

BMS scores were performed after spinal cord injury model establishment and cell transplantation to evaluate the recovery of motor function in the hind limbs of mice. The mice were scored using a double-blind method to observe ankle joint movements, hind limb support of the hindquarters and forward stepping, hind paw position and anterior and posterior limb coordination during stepping and trunk stability. The higher the score according to the BMS scale, the better the recovery.

## GFP Immunofluorescence Assay

Spinal cord tissue was fixed in 4% paraformaldehyde for more than 24 hours and dehydrated with gradient concentration of sucrose. Sections of 8μm thickness were then prepared on a frozen section machine and finally observed under a fluorescent microscope and images were collected for analysis.

## Histological analysis

After the tissue is fixed, dehydrated and embedded in sections, stained with hematoxylin and eosin, and finally dehydrated and mounted.

The tissue was fixed, dehydrated and embedded in sections and then placed in

citric acid antigen repair buffer (pH 6.0) for antigen repair. Next, endogenous peroxidase is blocked; the serum is blocked; the primary antibody (MMP2, CXCR4, BOSTER,China) is incubated overnight at 4°C; the secondary antibody (absin,China) is incubated at room temperature for 50 min with the appropriate species of primary antibody; the nuclei are double-stained after DAB (BOSTER,China)development. Final dehydration and sealing of the slides. Microscopic examination under a microscope and image acquisition for analysis.

## Western Blot Analysis.

Appropriate amount of RIPA (BOSTER, China) was added to the transfected cells and spinal cord tissues for 3-5 min to completely lyse the cells and tissues, and the supernatant was collected after centrifugation, which was the total protein. After measuring the protein concentration with the BCA protein concentration kit (BOSTER,China), the same amount of protein was separated by SDS-PAGE, and the protein was transferred to the PVDF membrane. Block with 5% bovine serum albumin (BOSTER,China) for 1.5 hours, and incubate the primary antibody overnight at 4°C (MMP2 antibody dilution ratio 1:10000, CXCR4 antibody dilution ratio 1:1000, BOSTER,China). The second antibody (goat anti-rabbit IgG labelled with HRP, dilution 1:5000, absin,China) was incubated at room temperature. Western blot analyses were repeated for 3 experiments.

## Statistical analysis

Software analysis was performed using SPSS 26.0 software (IBM, NY, USA). The results were analyzed using a one-way ANOVA (multiple comparisons) and data are expressed as mean ± standard deviation. Statistical graphs were produced by GraphPad Prism8 software (GraphPad , CA , USA). $P < 0.05$ indicates a significant difference.

# Result

## BMSCs cultured in vitro positively express CD90 and CD44, and negatively express CD34

BMSCs are easy to obtain and amplify in vitro. Under an inverted microscope the cells were consistent in morphology, resembling fibroblast-like cells that were spindle-shaped and formed colonies (Fig 1B). Under the fluorescence inverted microscope, BMSCs showed the characteristic green fluorescence of e GFP (Fig 1C). Cellular immunofluorescence identification of BMSCs cells showed characteristic positive expression of CD90 and CD44 (98.4%,98.66%) and negative expression of CD34 (0.14%) (Fig 1D-E).

**Fig 1. Extraction and identification of green fluorescent protein-labeled bone marrow mesenchymal stem cells. A** Schematic diagram of cell extraction. **B** Cell morphology under an optical microscope (x100 magnification). Scar bar: 100μm. **C** Cell morphology under a fluorescent

microscope (x100 magnification). Scar bar: 100μm. **D** The surface markers of BMSCs were identified by cellular immunofluorescence. CD90 and CD44 showed red fluorescence as antigen-positive expression; CD34 did not show red fluorescence as antigen-negative expression. Scar bar: 50μm. **E** Statistical results on the expression of surface markers CD90, CD44 and CD34 in BMSCs. Dates are presented as mean ± SD.

**Fig 2. miR-31 transfection of BMSCs. A** Schematic diagram of cell transfection. **B** Expression of miR-31 in bone marrow mesenchymal stem cells in each group after transfection was detected by qRT-PCR. **C** The status of miR-31agomir and miR-31antagomir transfection is detected by immunofluorescence, and successful transfection emits red fluorescence. Scar bar: 100μm. **D** Statistics of the results of miR-31agomir and miR-31antagomir transfection efficiency. **E** Morphology of the cells under optical microscopy after transfection. Scar bar: 100μm. Dates are presented as mean ± SD. Statistical analysis: compared to control, $^{**}P<0.01$, $^{***}P<0.001$.

**Fig 3. Effect of miR-31 on BMSCs proliferation. A** To analyze the effect of miR-31 on the proliferation of BMSCs. The OD values of miR-31agomir, miR-31antagomir and control at 450m for 24, 48 and 72 hours were detected by CCK-8. **B** Detection of the cell cycle distribution of each group of cells by flow cytometry. Dates are presented as mean ± SD. Statistical analysis: within group compared to control, $^{*}P<0.05$, $^{**}P<0.01$, $^{***}P<0.001$;

between groups compared to 24 hours miR-31agomir, $^{\#}P<0.05$, $^{\#\#}P<0.01$.

**Fig 4. Analysis of the effect of miR-31 on the migration of BMSCs. A** The promoted effect of miR-31 on the horizontal migration of BMSCs tested by bidirectional wound-healing assay at 0, 12, 24 and 48 hours. Scar bar: 100μm. **B** Percentage cell migration area result statistics for control, mir-31agomir and miR-31antagomir at 0, 12, 24 and 48 hours. **C** Schematic diagram of the vertical cell migration tested by Transwell chamber assay. **D** Cells that passed through the polycarbonate membrane were stained with crystal violet, observed under an inverted phase contrast microscope and counted in 200× magnification field. Scar bar: 50μm. **E** Statistical results of the number of cells in the lower chamber of the migration value of BMSCs. Dates are presented as mean ± SD. Statistical analysis: within group compared to control, $^{*}P<0.05$, $^{**}P<0.01$, $^{***}P<0.001$; between groups compared to 12 hours miR-31agomir, $^{\#\#\#}P<0.001$.

**Fig 5. In vitro, miR-31 upregulated CXCR4 and MMP-2 expression. A** Strip chart of protein expression. **B** The relative expression of CXCR4 protein in each group. **C** The relative expression of MMP-2protein in each group. Dates are presented as mean ± SD. Statistical analysis: compared to control, $^{*}P<0.05$, $^{**}P<0.01$, $^{***}P<0.001$.

**Fig 6. Establishment of SCI model and the effect of miR-31 on BMSCs migration in vivo. A** Schematic diagram of cell transplantation. **B** Relative levels of IL-6 and IL-17 in normal and spinal cord injured mice. **C**

Assessment of functional repair after spinal cord injury in mice by BMS score. **D** The morphology of the normal and post-injured spinal cord was observed by HE staining. The arrow in the normal spinal cord points to the central cord and neurons; the arrow in the injured tissue points to the red blood cells and vacuoles. Scar bar: 50μm. **E** The migration of bone marrow mesenchymal stem cells within the spinal cord was observed by frozen sections on days 5 and 7. Scar bar: 50μm. **F** Statistical results of the relative fluorescence intensity of migrating BMSCs within the spinal cord. Dates are presented as mean ± SD. Statistical analysis: Within group compared to control, $^{*}P<0.05$, $^{**}P<0.01$, $^{***}P<0.001$; between groups compared to 5 days miR-31agomir, $^{\#\#\#}P<0.01$; $^{a}$ $P<0.05$ indicates miR-31antagomir compared to miR-31agomir.

**Fig 7. miR-31 upregulated the expression of CXCR4 and MMP-2. A** After cell transplantation, CXCR4 expression in tissues was detected by immunohistochemistry assays on days 5 and 7. Scar bar: 25μm. **B** Statistical results of CXCR4 expression in tissues. **C** After cell transplantation, MMP-2 expression in tissues was detected by immunohistochemistry assays on days 5 and 7. Scar bar: 25μm. **D** Statistical results of MMP-2 expression in tissues. **E** IL-17 expression in vivo after cell transplantation. **F** IL-6 expression in vivo after cell transplantation. **G** After cell transplantation, CXCR4/MMP-2 protein expression was assessed in vivo by Western blot. Compared to control,

$^{*}P<0.05$. Dates are presented as mean ± SD. Statistical analysis: within group compared to control, $^{*}P<0.05$, $^{**}P<0.01$, $^{***}P<0.001$; between groups compared to 5 days miR-31agomir, $^{\#\#}P<0.01$, $^{\#\#\#}P<0.001$.

## miR-31 transfected BMSCs

We customized miR-31agomir and miR-31antagomir which were modified by high affinity cholesterol were stable and fluorescently labeled with Cy3. The red fluorescence was directly observed under fluorescence microscope after making transfection of BMSCs (Fig 2C). The transfection efficiency was 93.27% in the miR-31agomir group and 94.44% in the miR-31antagomir group, with no statistically significant difference between the two groups (Fig 2D). The results of qRT-PCR experiments showed that after cell transfection, the expression of miR-31 was significantly increased in the miR-31agomir group compared with the control group (P<0.001) and was more than 7-fold higher, however, the expression of miR-31 was restricted in the miR-31antagomir group (P<0.01) (Fig 2B). The cell morphology after transfection did not change (Fig 2E).

## miR-31 promotes the proliferation of BMSCs

In order to study the effect of miR-31 on the proliferation of BMSCs, we did CCK-8 and Cell-cycle assay. As shown in Fig 3A, the proliferation of BMSCs was significantly increased in the miR-31agomir group compared with the control group (P<0.05) and in a time-dependent manner (P<0.001). However, the proliferation of

BMSCs in the miR-31antagomir group was inhibited at 72 hours compared with the control group (P<0.001). Flow cytometry results showed that in the S phase, the percentage of cells in the miR-31agomir group was 22.45%, miR-31antagomir was 16.85% and the control group was 19.68% (Fig 3B). In summary, miR-31 can promote the proliferation of BMSCs.

## miR-31 promotes the migration of BMSCs in vitro

To observe the effect of miR-31 on the horizontal migration of cells, we analyzed the migration ability by wound-healing assay after transfection of the cells, which has been widely used to detect cell migration in vitro. At 24 hours, compared with the control group, the miR-31agomir group migrated faster (P<0.01). At 48 hours, the cell migration rate was significantly faster in the miR-31agomir group than in the control group, with a large difference (P<0.001). And the migration speed of miR-31agomir group cells accelerated with the increase of time (Fig 4A). Statistical analysis of the wound healing area showed that the migration ability of BMSCs was significantly enhanced after transfection with miR-31agomir (Fig 4B).

In order to further confirm the effect of miR-31 on the vertical migration of cells, a Transwell chamber assay was used. The transfected cells were seeded in the upper chamber of Transwell and cultured for 24 hours (Fig 4C). As shown in Fig 4D, compared to the control group, more cells of the miR-31agomir experimental group migrated to the lower chamber of the Transwell. Additionally, the quantitative analysis shown in Fig 4E indicated that miR-31agomir could significantly enhance the migration

of BMSCs ($p<0.001$). These experimental results suggest that miR-31 can promote the migration of bone marrow mesenchymal stem cells, laying the foundation for repairing damaged tissue function.

## In vitro, miR-31 upregulated the expression of CXCR4/MMP-2 protein

To further investigate the mechanism by which miR-31 promotes the migration of BMSCs, CXCR4 and MMP-2 were analyzed. Western blot results showed that the relative expression of CXCR4 protein was higher in the miR-31agomir group ($P<0.01$) and lower in the miR-31antagomir group ($P<0.05$) compared to the control group (Fig 5B). Similarly, the relative expression of MMP-2 protein and CXCR4 expression trends were broadly consistent (Fig 5C).

## Evaluation of miR-31 repair of spinal cord injury function by BMS score

To evaluate animal models of spinal cord injury, we performed BMS scores, inflammatory factor assays, and hematoxylin-eosin staining experiments. After the model was established, serum inflammatory factors were measured and the results showed that the relative levels of both IL-6 and IL-17 were higher in SCI mice ($P<0.05$, $P<0.001$) than in normal mice (Fig 6B). The BMS score result was 0 (Fig 6C). As shown in Fig 6D, the normal spinal cord was morphologically intact with clear boundaries of gray and white matter, abundant neurons, and an intact central cord canal with no red

blood cells, inflammatory cells or vacuoles. However, the morphological structure of the damaged spinal cord was missing, the boundaries of gray and white matter were blurred, the number of neurons was reduced, and erythrocytes, inflammatory cells, and vacuoles were scattered. And these experiments indicated that the SCI model was successfully made.

After cell transplantation, the BMS scores were significantly higher in the miR-31agomir group compared to the control and miR-31antagomir groups, and the difference was most significant on days 5 and 7 ($P<0.001$) (Fig 6C). This result indicated that miR-31 could promote the recovery of function in mice with spinal cord injury.

## In vivo, miR-31 can promote the migration of BMSCs

To evaluate the effect of miR-31 on the migration of BMSCs in vivo, spinal cord tissues were selected for frozen sections on day 5 and day 7 after cell transplantation. As shown in Fig 6E-F, the mean fluorescence intensity in the tissue was higher in the miR-31 group compared to the control group and miR-31antagomir, and the fluorescence intensity was enhanced in a time-dependent manner($P<0.001$). The results suggest that in vivo miR-31 has a facilitative effect on the migration of BMSCs.

## In vivo, the effect of miR-31 on the protein levels of CXCR4 and MMP-2

To assess the expression of inflammatory factors after cell transplantation

treatment, we measured the relative levels of IL-6 and IL-17 again on days 5 and 7, respectively. Compared with the control group, the relative content expression of both IL-6 and IL-17 was reduced in the miR-31agomir group ($P<0.05$, $P<0.01$, $P<0.001$), with a decreasing trend with time (Fig 7E-F). The results indicated that the anti-inflammatory effect of pretreatment of BMSCs before transplantation would be relatively enhanced.

For further investigation of the mechanism of miR-31 on the migration of BMSCs in vivo, we analyzed CXCR4 and MMP-2. Immunohistochemical results showed a higher number of CXCR4-positive cells in the miR-31agomir group($P<0.001$) compared to the control group, with a time-dependent increase ($P<0.001$) (Fig 7A-B). Similarly, the expression of MMP-2 was consistent with the trend of CXCR4 expression (Fig 7C-D). The Western blot results showed that the relative expression of CXCR4 and MMP-2 proteins was higher in the miR-31agomir group($P<0.05$) compared with the control group (Fig 7G). These experiments showed that miR-31 upregulated the expression of CXCR4 and MMP-2 proteins.

# Discussion

Today, BMSCs transplants are widely used in the treatment of SCI(18). Although BMSCs promote functional repair after SCI by secreting various trophic factors and functionally useful growth factors, the biggest shortcoming of BMSCs transplantation is that they do not effectively migrate to the target tissues(19, 20). Hypoxia or trophic factors (stromal cell-derived factor-1, monocyte chemotactic protein-1) have been

shown to promote the migration of bone marrow mesenchymal stem cells in vitro(21, 22). However, there are few studies on the effect of miR-31 on the migration of BMSCs. Therefore, in our study, cells were pretreated with miR-31 SiRNA to enhance the migration potential of bone marrow mesenchymal stem cells.

MicroRNAs (miRNAs) are small, non-coding RNA molecules that target mRNAs to silence gene expression at the post-transcriptional level and play an important role in a variety of cellular biological processes(12, 23). Research has shown that miRNAs, which are used in the study of stem cells, tumor cells and developmental regulation, can regulate the expression of a wide range of genes and play a key role in the biology of stem cells(24-26). Such as Satoshi et al. found that miR-720 promotes differentiation and reduces proliferation of dental pulp stem/progenitor cells by inhibiting the Nanog homologous frame (Nanog)(27); Xiao et al. found that miR-146 plays an important role in the regulation of stem cell proliferation, differentiation and apoptosis(28); Kong et al. found that microRNA-126 promotes proliferation and migration of bone marrow mesenchymal stem cells(29). Studies have found that miR-31 is involved in the biological process of cells, especially the invasion and migration of tumor cells(30). However, there are few studies on the effect of bone marrow mesenchymal stem cell migration.

MiR-31 is highly conserved among species and it can be speculated that miR-31 may play an important role in the proliferation and migration of BMSCs. In our study, miR-31 SiRNA was successfully transfected in BMSCs and the upregulation of miR-31 expression in cells was confirmed by RT-qPCR. After comparing the migration

ability of each group of BMSCs by Transwell and cell scratch assay, miR-31 was found to promote the migration of BMSCs. This result lays the foundation for efficient migration of BMSCs to target tissues. The migration process of BMSCs requires the synergistic action of several chemokines, adhesion factors and proteases, the most important of which are matrix metalloproteinase2 (MMP-2) and CXC chemokine receptor type 4 (CXCR4)(31). Matrix metalloproteinases (MMPs) are a family of proteases that use zinc metal ions as cofactors and whose main function is to degrade and maintain the homeostasis of the extracellular matrix(32). Among them, MMP-2 is the most important protein in the matrix metalloproteinase family and is also an important protein involved in cell migration. It plays a specific role in cell migration and proliferation by degrading collagen of the basement membrane(33).The chemokine receptor family is the most tissue-specific and subpopulation-selective family of cell homing receptors, of these, CXCR4 has been shown to have a key role in injury-induced stem cell recruitment and a strong chemotactic role in regulating bone marrow MSC migration(34, 35). In this study, the results of Western blot experiments showed that the up-regulation of miR-31 in BMSCs will up-regulate the expression of MMP-2 and CXCR4 proteins, which is a prerequisite for efficient targeted migration after cell transplantation.

In recent years, it has been found that pretreatment of BMSCs prior to cell transplantation can promote the migration of BMSCs, such as Li-Ki et al. found that lithium preparations promote the migration of BMSCs to restore neurological function in cerebral ischemia by inducing the expression of CXCR4 in BMSCs(36). However,

current stem cell experiments do not give us information about the migration of transplanted cells in the target tissue(1). Therefore, in this experiment, we chose to use BMSCs with green fluorescent protein and pretreated with miR-31 SiRNA for in vivo experiments. After the spinal cord injury model was successfully established, the migration of BMSCs in the damaged tissues and the expression of the associated migrating proteins were observed by collecting tissues for frozen sections and immunohistochemistry as well as Western blot detection. And the results showed that more BMSCs migrated into damaged tissues after pretreatment with miR-31agomir compared with those without pretreatment ($P<0.01$); the experimental group of miR-31agomir MMP-2 and CXCR4 positive cells and proteins than the control group($P<0.001$). The results of in vivo experiments indicated that the upregulation of MMP-2 and CXCR4 expression after cell transplantation was an important factor in improving the migration and homing of transplanted cells. Studies have shown that mesenchymal stem cells injected into mice can be located in the area of spinal cord injury. These cells can protect neurons, promote axon regeneration, and restore function to a certain extent(11). Previous studies by our group have found that miR-31 promotes functional improvement and repair after spinal cord injury in mice(17, 37). The prognosis of spinal cord injury and inflammation are closely related, and interleukin-6 and interleukin-17 are two important pro-inflammatory factors; therefore, we examined the relative levels of the two factors after animal model establishment and cell transplantation treatment, respectively, and the results showed that the levels of IL-6 and IL-17 were increased after injury and decreased after pretreatment transplantation

of BMSCs, which suggested that pretreatment was more useful for disease recovery after cell transplantation. In this experiment, the results of BMS scores reconfirmed that cell modified by miR-31 could promote functional repair of damaged tissues. It may be that miR-31 can inhibit the proliferation of glial cells after SCI. In conclusion, miR-31 promotes the migration of BMSCs and upregulates the expression of MMP-2 and CXCR4, which in turn promotes the repair after nerve injury.

This study specifies the effect of miR-31 on the migration of BMSCs through two parts of in vivo and in vitro experiments. The strength of the current study lies in the in vivo experiments performed as well as the use of siRNA small molecule nucleic acid drugs. RNA interference is a natural phenomenon that can affect the regulation of gene transcription, and siRNAs have become an important tool for RNAi technology to achieve gene silencing by transfecting them into cells to regulate the expression of target genes(38). SiRNA has also emerged as a potential and promising therapeutic platform and has been applied in many research areas. In this study, we customized miR-31 agomir and antagomir, which were modified to increase stability and ease of transfection and were fluorescently labeled with Cy3, as miR-31 siRNA drugs, and verified the effect of miR-31 on migration of BMSCs by pretreatment of bone marrow mesenchymal stem cells. And through the study, we found that the customized miR-31 siRNA drugs exerted the same effects as common siRNA inhibitors and had a wide range of clinical applications. Thus, promoting the migration of BMSCs by miR-31 provides a new therapeutic strategy for the treatment of neurodegenerative diseases such as spinal cord injury. It also lays the preliminary foundation for us to effectively

use miR-31-related siRNA drugs for clinical treatment of spinal cord injury and motor neuron diseases.

In this study, in vivo and in vitro experiments demonstrated that miR-31 can promote the migration of bone marrow mesenchymal stem cells and that miR-31 can repair and improve the function of damaged tissues in spinal cord injury.

# Conclusions

It was shown that transfection of bone marrow mesenchymal stem cells with miR-31agomir promotes their cell migration and that miR-31 can repair and improve the function of damaged tissues in spinal cord injury.

**Declarations**

**Ethics approval and consent to participate**

All experimental procedures involving animals were approved by the Ethical Research Committee of the Laboratory Animal Center of Shanxi Medical University (Approval No. SYDL2021014) and the Regulations of the People's Republic of China on the Administration of Laboratory Animals.

**Competing interests**

The authors declare that they have no competing interests.

**Acknowledgements**

The authors would like to thank the funders listed in the funding section for their support. The authors would like to thank Professor Chunfang Wang for helpful discussions.

**Funding**

National Natural Science Foundation of China(81371384， 82001326);

Applied Basic Research Project of Shanxi Province (201901D211319);

Key Laboratory Opening Project of Shanxi Province (KF2020-02).

**Authors' contributions**

Yujuan Zhang: Methodology, Formal analysis, Resources, Visualization, Writing – original draft.

Lili Cao: Methodology, Software.

Ruochen Du: Methodology, Writing – review & editing.

Yitong Yuan: Methodology, Writing – review & editing, funding acquisition.

Xiao-Bo Gong: Methodology, Writing – review & editing.

Chunfang Wang: Conceptualization, Writing – review & editing, Funding acquisition.

Feng Tian: Supervision.

Xiao Li: Supervision.

# References:

1. Assinck P, Duncan GJ, Hilton BJ, Plemel JR, Tetzlaff W. Cell transplantation therapy for spinal cord injury. Nat Neurosci. 2017;20(5):637-47. http://doi.org/10.1038/nn.4541
2. Basso DM, Fisher LC, Anderson AJ, Jakeman LB, McTigue DM, Popovich PG. Basso Mouse Scale for locomotion detects differences in recovery after spinal cord injury in five common mouse strains. J Neurotrauma. 2006;23(5):635-59. http://doi.org/10.1089/neu.2006.23.635
3. Xie XK, Xu ZK, Xu K, Xiao YX. DUSP19 mediates spinal cord injury-induced apoptosis and inflammation in mouse primary microglia cells via the NF-kB signaling pathway. Neurol Res. 2020;42(1):31-8. http://doi.org/10.1080/01616412.2019.1685068
4. Fei M, Li Z, Cao Y, Jiang C, Lin H, Chen Z. MicroRNA-182 improves spinal cord injury in mice by modulating apoptosis and the inflammatory response via IKKbeta/NF-kappaB. Lab Invest. 2021;101(9):1238-53. http://doi.org/10.1038/s41374-021-00606-5
5. Gazdic M, Volarevic V, Harrell CR, Fellabaum C, Jovicic N, Arsenijevic N, et al. Stem Cells Therapy for Spinal Cord Injury. Int J Mol Sci. 2018;19(4). http://doi.org/10.3390/ijms19041039
6. Wu LL, Pan XM, Chen HH, Fu XY, Jiang J, Ding MX. Repairing and Analgesic Effects of Umbilical Cord Mesenchymal Stem Cell Transplantation in Mice with Spinal Cord Injury. Biomed

Res Int. 2020;2020:7650354. http://doi.org/10.1155/2020/7650354

7. Ra JC, Shin IS, Kim SH, Kang SK, Kang BC, Lee HY, et al. Safety of intravenous infusion of human adipose tissue-derived mesenchymal stem cells in animals and humans. Stem Cells Dev. 2011;20(8):1297-308. http://doi.org/10.1089/scd.2010.0466
8. Wang Y, Sun X, Lv J, Zeng L, Wei X, Wei L. Stromal Cell-Derived Factor-1 Accelerates Cartilage Defect Repairing by Recruiting Bone Marrow Mesenchymal Stem Cells and Promoting Chondrogenic Differentiation<sup/>. Tissue Eng Part A. 2017;23(19-20):1160-8. http://doi.org/10.1089/ten.TEA.2017.0046
9. Shirjang S, Mansoori B, Solali S, Hagh MF, Shamsasenjan K. Toll-like receptors as a key regulator of mesenchymal stem cell function: An up-to-date review. Cell Immunol. 2017;315:1-10. http://doi.org/10.1016/j.cellimm.2016.12.005
10. Yi XM, Chen Y, Tu GJ. Neuregulin1 impacting bone marrow mesenchymal stem cell migration is conducive to functional recovery following spinal cord injury. Mol Med Rep. 2019;20(1):41-8. http://doi.org/10.3892/mmr.2019.10217
11. Li H, Wang C, He T, Zhao T, Chen YY, Shen YL, et al. Mitochondrial Transfer from Bone Marrow Mesenchymal Stem Cells to Motor Neurons in Spinal Cord Injury Rats via Gap Junction. Theranostics. 2019;9(7):2017-35. http://doi.org/10.7150/thno.29400
12. Bartel DP. Metazoan MicroRNAs. Cell. 2018;173(1):20-51. http://doi.org/10.1016/j.cell.2018.03.006
13. Jiang WL, Zhao KC, Yuan W, Zhou F, Song HY, Liu GL, et al. MicroRNA-31-5p Exacerbates Lipopolysaccharide-Induced Acute Lung Injury via Inactivating Cab39/AMPKalpha Pathway. Oxid Med Cell Longev. 2020;2020:8822361. http://doi.org/10.1155/2020/8822361
14. Peng H, Wang L, Su Q, Yi K, Du J, Wang Z. MiR-31-5p promotes the cell growth, migration and invasion of colorectal cancer cells by targeting NUMB. Biomed Pharmacother. 2019;109:208-16. http://doi.org/10.1016/j.biopha.2018.10.048
15. Hu J, Chen C, Liu Q, Liu B, Song C, Zhu S, et al. The role of the miR-31/FIH1 pathway in TGF-beta-induced liver fibrosis. Clin Sci (Lond). 2015;129(4):305-17. http://doi.org/10.1042/CS20140012
16. Salvatore D. Deiodinases and stem cells: an intimate relationship. J Endocrinol Invest. 2018;41(1):59-66. http://doi.org/10.1007/s40618-017-0737-4
17. Li X, Gao Y, Tian F, Du R, Wang C. miR-31 promotes neural stem cell proliferation and restores motor function after spinal cord injury. Exp Biol Med. 2021;246(11):531302299.
18. Wang Y, Kong QJ, Sun JC, Xu XM, Yang Y, Liu N, et al. Protective effect of epigenetic silencing of CyclinD1 against spinal cord injury using bone marrow-derived mesenchymal stem cells in rats. J Cell Physiol. 2018;233(7):5361-9. http://doi.org/10.1002/jcp.26354
19. Lin L, Lin H, Bai S, Zheng L, Zhang X. Bone marrow mesenchymal stem cells (BMSCs) improved functional recovery of spinal cord injury partly by promoting axonal regeneration. Neurochem Int. 2018;115:80-4. http://doi.org/10.1016/j.neuint.2018.02.007
20. Ma C, Wang J, Fan L. Therapeutic effects of bone mesenchymal stem cells on oral and maxillofacial defects: a novel signaling pathway involving miR-31/CXCR4/Akt axis. J Recept Signal Transduct Res. 2019;39(4):321-30. http://doi.org/10.1080/10799893.2019.1669054
21. Haider H, Ashraf M. Strategies to promote donor cell survival: combining preconditioning approach with stem cell transplantation. J Mol Cell Cardiol. 2008;45(4):554-66. http://doi.org/10.1016/j.yjmcc.2008.05.004

22. Meng Z, Feng G, Hu X, Yang L, Yang X, Jin Q. SDF Factor-1alpha Promotes the Migration, Proliferation, and Osteogenic Differentiation of Mouse Bone Marrow Mesenchymal Stem Cells Through the Wnt/beta-Catenin Pathway. Stem Cells Dev. 2021;30(2):106-17. http://doi.org/10.1089/scd.2020.0165
23. Yang C, Wang Y, Hardy P. Emerging roles of microRNAs and their implications in uveal melanoma. Cell Mol Life Sci. 2021;78(2):545-59. http://doi.org/10.1007/s00018-020-03612-w
24. Hammond SM. An overview of microRNAs. Adv Drug Deliv Rev. 2015;87:3-14. http://doi.org/10.1016/j.addr.2015.05.001
25. Rupaimoole R, Slack FJ. MicroRNA therapeutics: towards a new era for the management of cancer and other diseases. Nat Rev Drug Discov. 2017;16(3):203-22. http://doi.org/10.1038/nrd.2016.246
26. Pichiorri F, Palmieri D, De Luca L, Consiglio J, You J, Rocci A, et al. Correction: In vivo NCL targeting affects breast cancer aggressiveness through miRNA regulation. J Exp Med. 2017;214(5):1557. http://doi.org/10.1084/jem.2012095001172017c
27. Hara ES, Ono M, Eguchi T, Kubota S, Pham HT, Sonoyama W, et al. miRNA-720 controls stem cell phenotype, proliferation and differentiation of human dental pulp cells. Plos One. 2013;8(12):e83545. http://doi.org/10.1371/journal.pone.0083545
28. Xiao WZ, Lu AQ, Liu XW, Li Z, Zi Y, Wang ZW. Role of miRNA-146 in proliferation and differentiation of mouse neural stem cells. Biosci Rep. 2015;35(5). http://doi.org/10.1042/BSR20150088
29. Kong R, Gao J, Ji L, Zhao D. MicroRNA-126 promotes proliferation, migration, invasion and endothelial differentiation while inhibits apoptosis and osteogenic differentiation of bone marrow-derived mesenchymal stem cells. Cell Cycle. 2020;19(17):2119-38. http://doi.org/10.1080/15384101.2020.1788258
30. Cai P, Li H, Huo W, Zhu H, Xu C, Zang R, et al. Aberrant expression of LncRNA-MIR31HG regulates cell migration and proliferation by affecting miR-31 and miR-31* in Hirschsprung's disease. J Cell Biochem. 2018;119(10):8195-203. http://doi.org/10.1002/jcb.26830
31. Zhang S, Liu Y, Derakhshanfar S, He W, Huang Q, Dong S, et al. Polymer Self-Assembled BMSCs with Cancer Tropism and Programmed Homing. Adv Healthc Mater. 2018;7(23):e1800118. http://doi.org/10.1002/adhm.201800118
32. Roderfeld M. Matrix metalloproteinase functions in hepatic injury and fibrosis. Matrix Biol. 2018;68-69:452-62. http://doi.org/10.1016/j.matbio.2017.11.011
33. Shen KH, Hung JH, Liao YC, Tsai ST, Wu MJ, Chen PS. Sinomenine Inhibits Migration and Invasion of Human Lung Cancer Cell through Downregulating Expression of miR-21 and MMPs. Int J Mol Sci. 2020;21(9). http://doi.org/10.3390/ijms21093080
34. Baek SJ, Kang SK, Ra JC. In vitro migration capacity of human adipose tissue-derived mesenchymal stem cells reflects their expression of receptors for chemokines and growth factors. Exp Mol Med. 2011;43(10):596-603. http://doi.org/10.3858/emm.2011.43.10.069
35. Lim K, Hyun YM, Lambert-Emo K, Capece T, Bae S, Miller R, et al. Neutrophil trails guide influenza-specific CD8(+) T cells in the airways. Science. 2015;349(6252):a4352. http://doi.org/10.1126/science.aaa4352
36. Tsai LK, Leng Y, Wang Z, Leeds P, Chuang DM. The mood stabilizers valproic acid and lithium enhance mesenchymal stem cell migration via distinct mechanisms. Neuropsychopharmacol. 2010;35(11):2225-37. http://doi.org/10.1038/npp.2010.97
37. Li P, Gao Y, Li X, Tian F, Wang F, Wang Y, et al. mRNA and miRNA expression profile reveals

the role of miR-31 overexpression in neural stem cell. Sci Rep. 2020;10(1):17537. http://doi.org/10.1038/s41598-020-74541-8
38. Han H. RNA Interference to Knock Down Gene Expression. Methods Mol Biol. 2018;1706:293-302. http://doi.org/10.1007/978-1-4939-7471-9_16

Click here to access/download;Figure;Figure1.tif

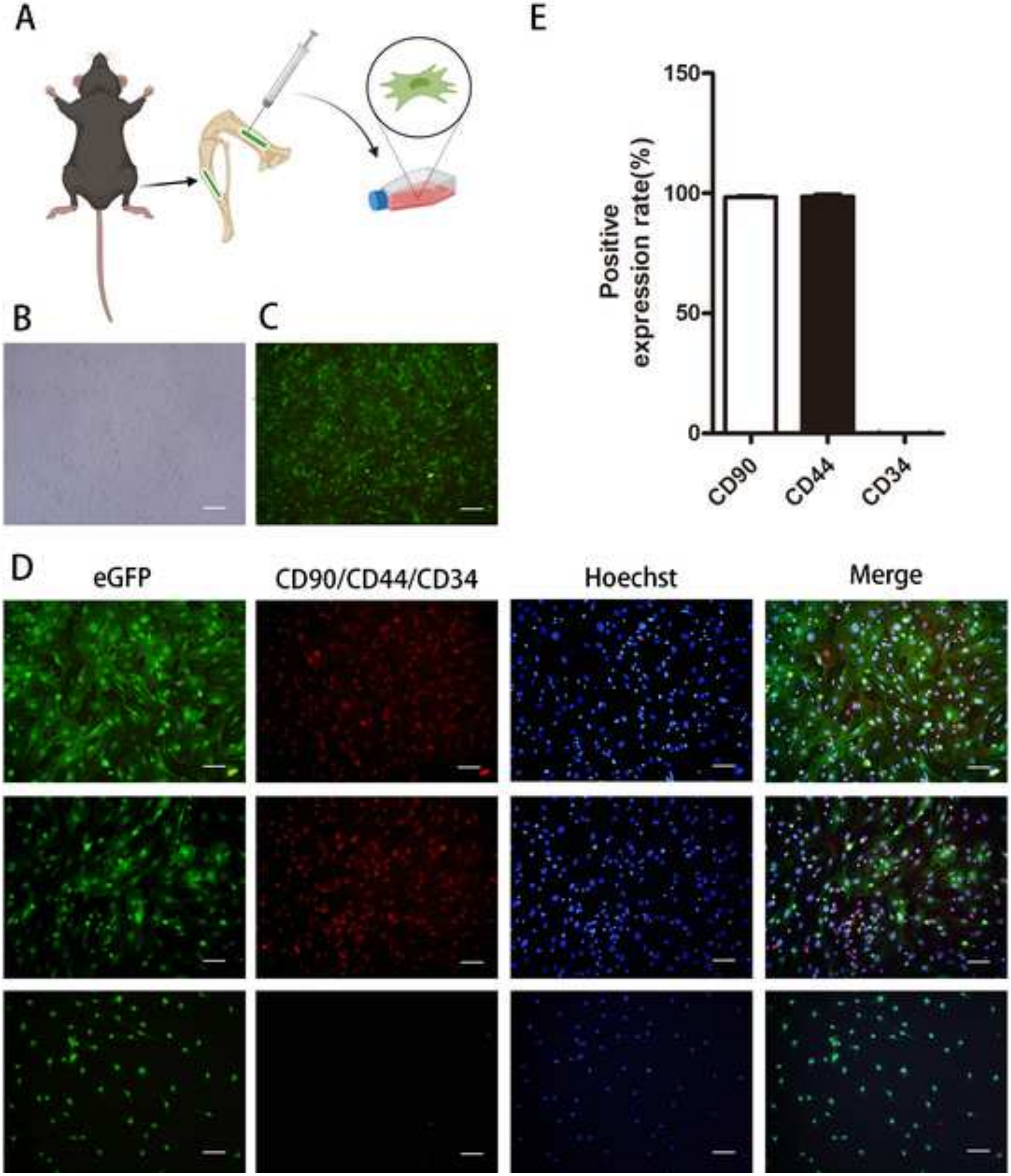

Click here to access/download;Figure;Figure2.tif

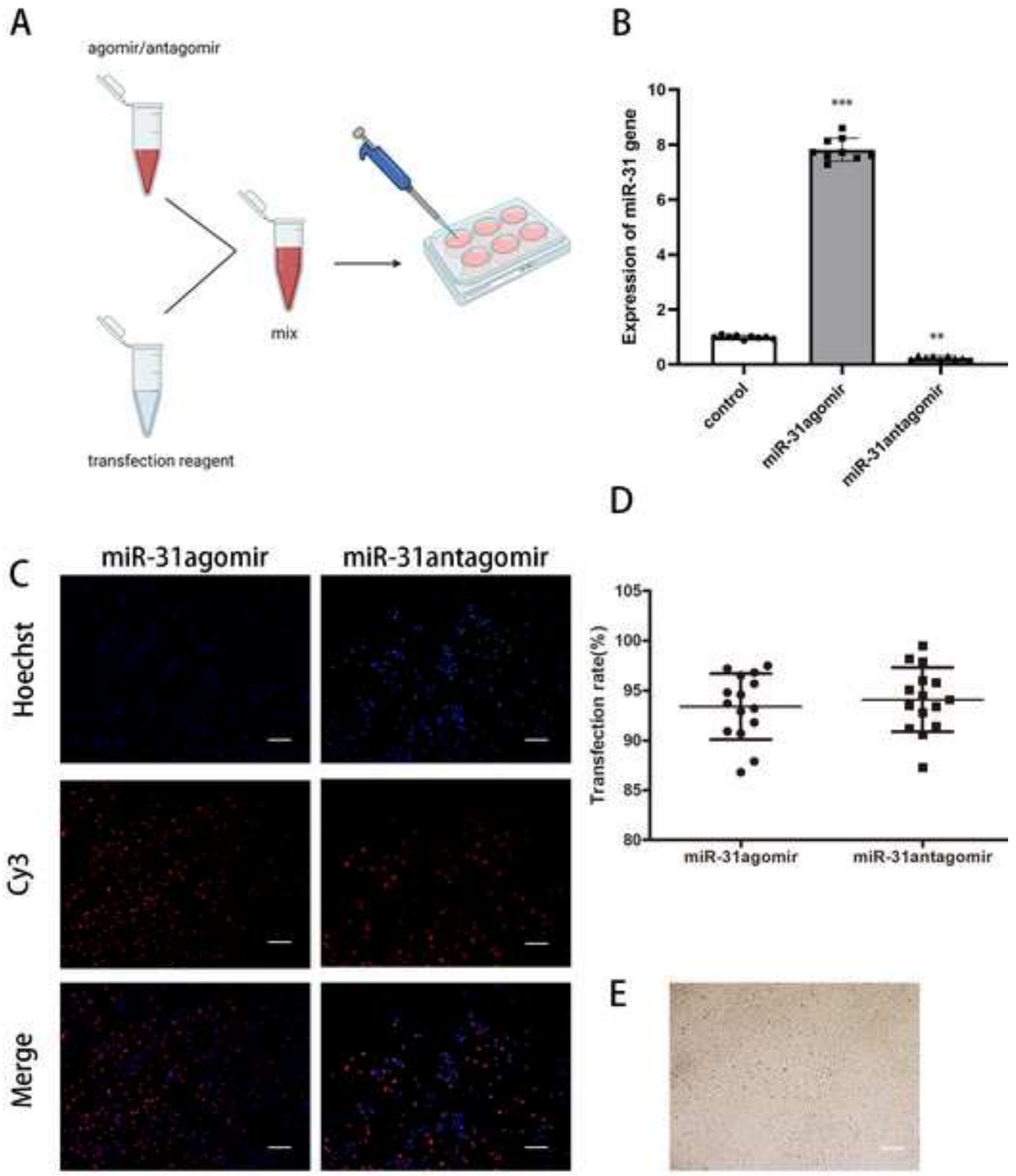

Click here to access/download;Figure;Figure3.tif

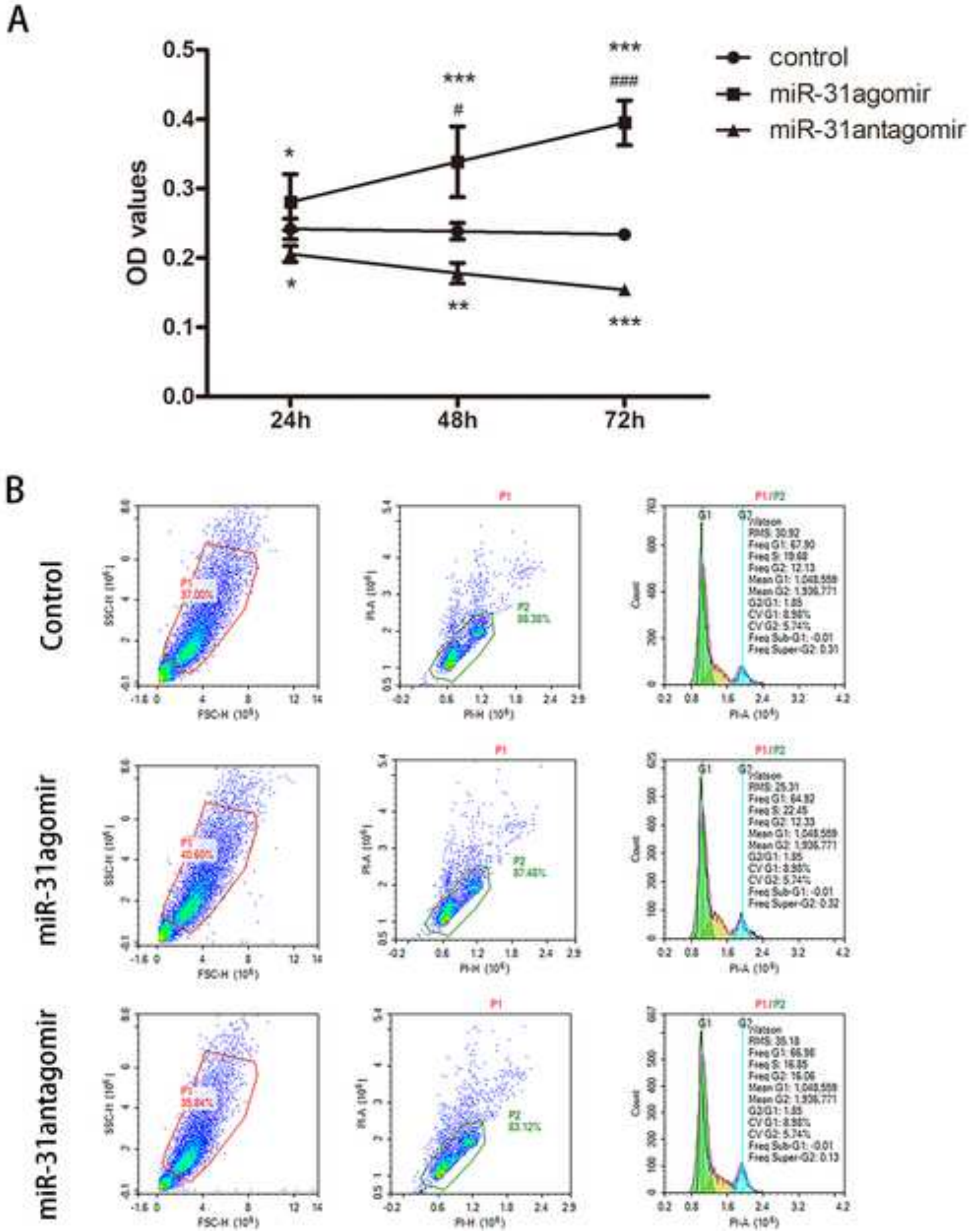

Click here to access/download;Figure;Figure4.tif

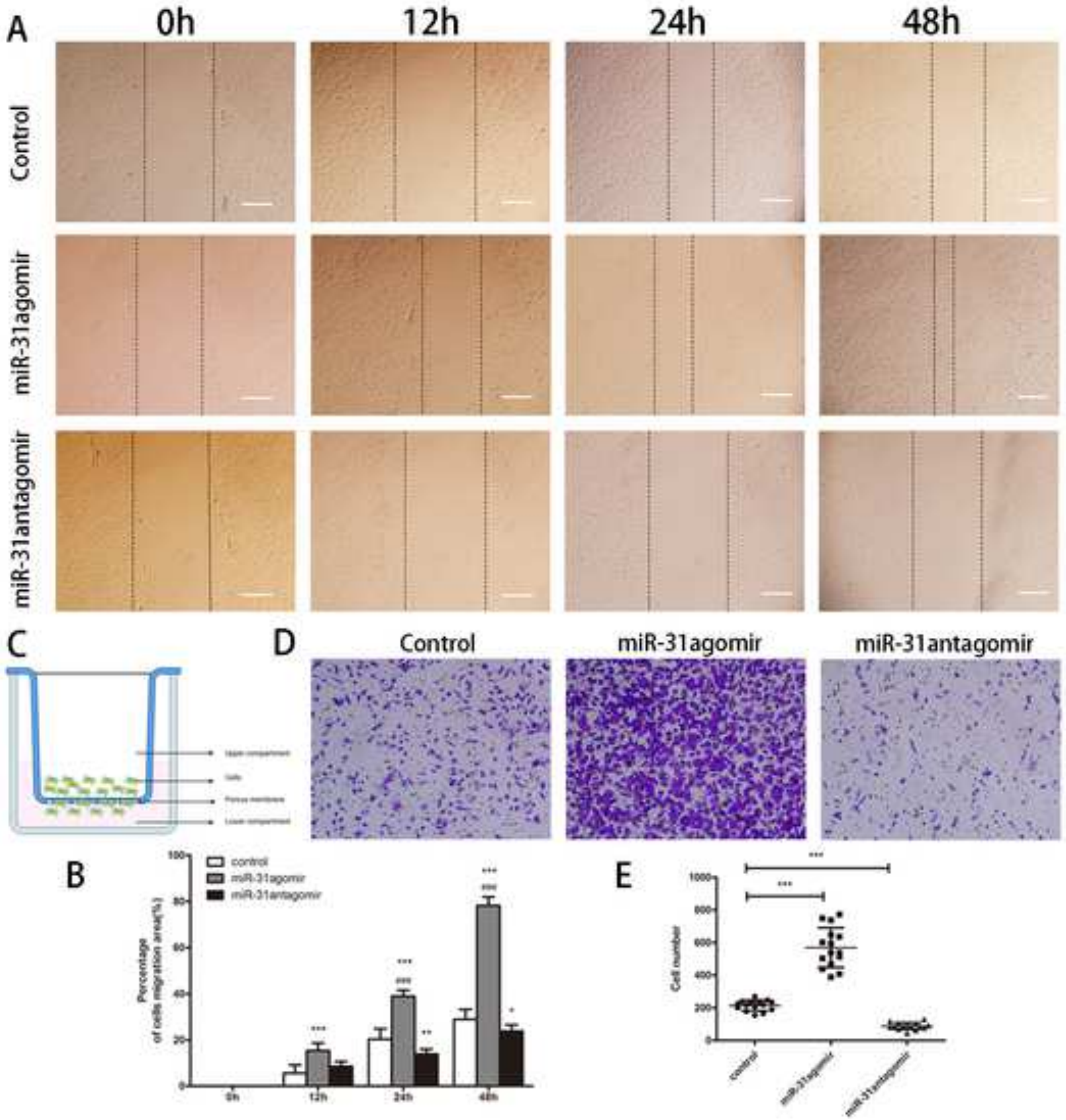

Click here to access/download;Figure;Figure5.tif

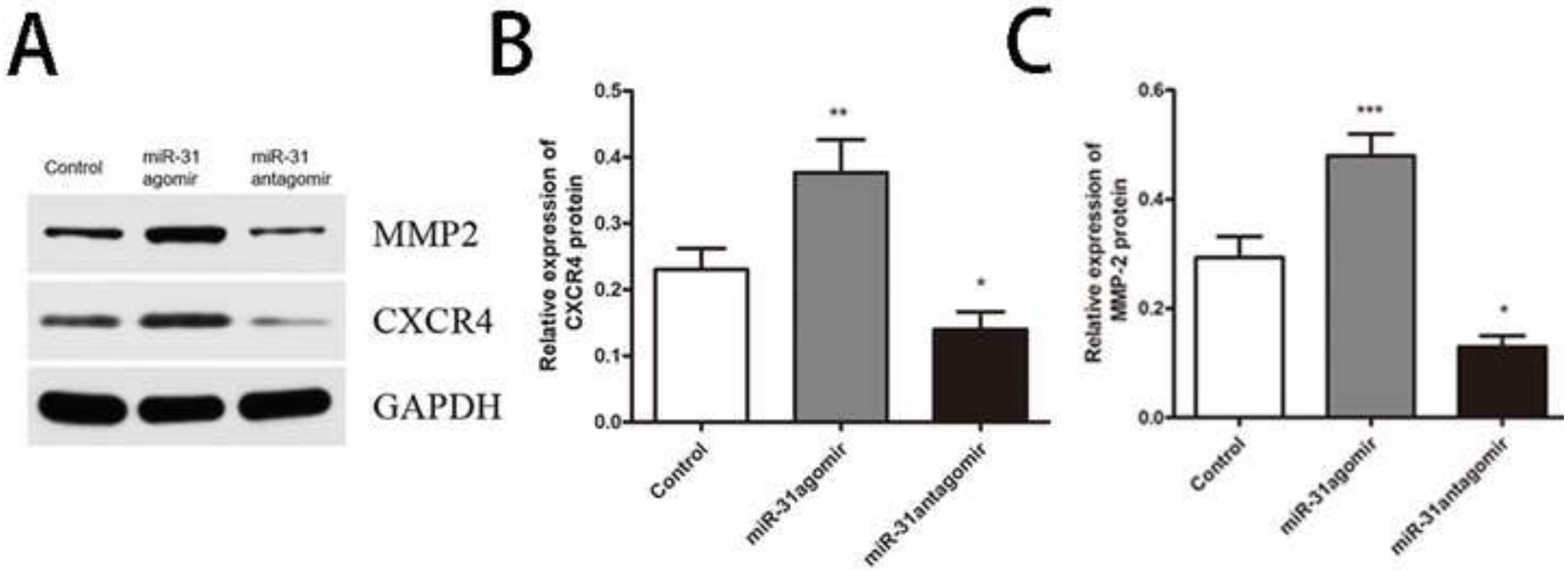

Click here to access/download;Figure;Figure6.tif

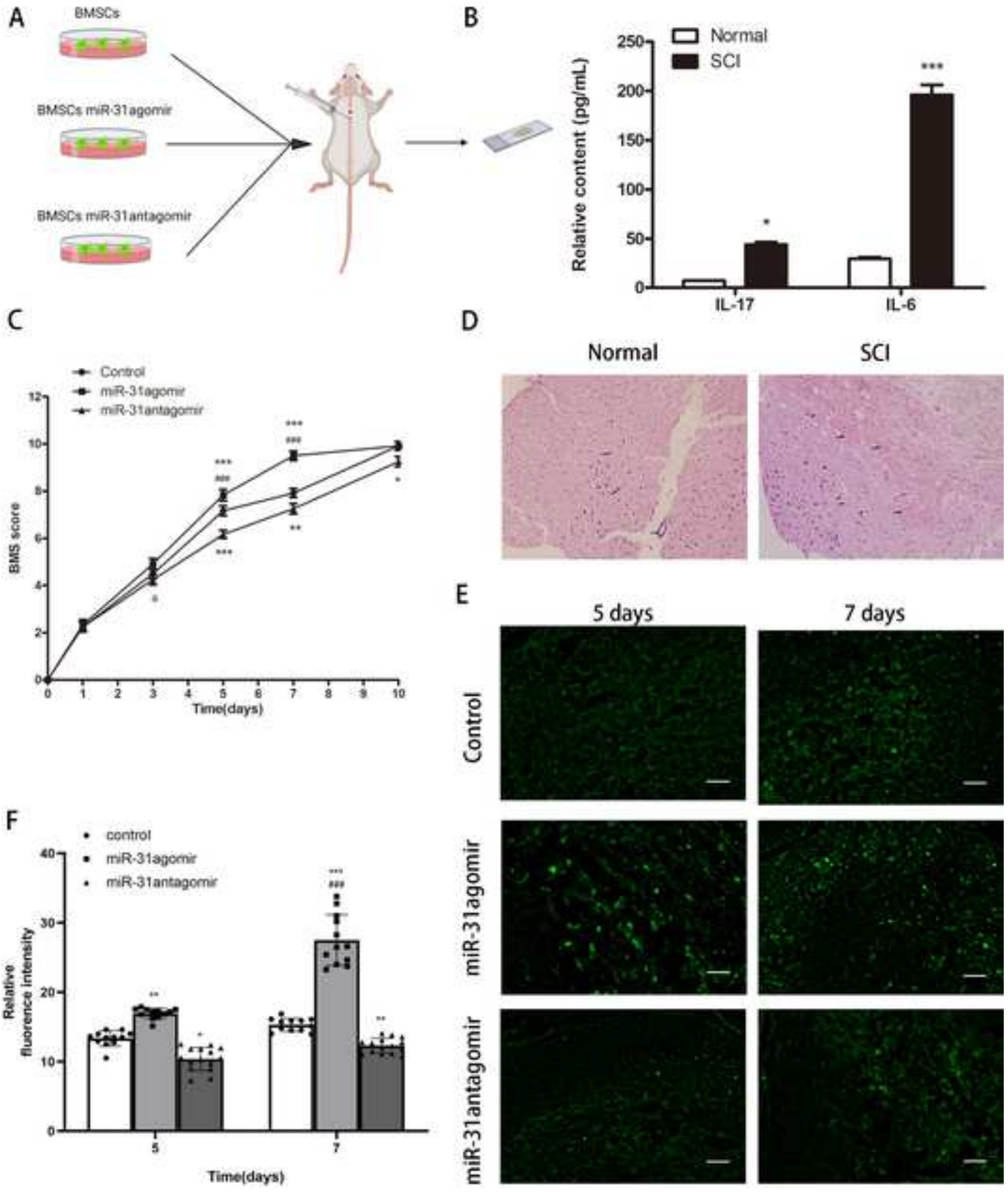

Click here to access/download;Figure;Figure7.tif

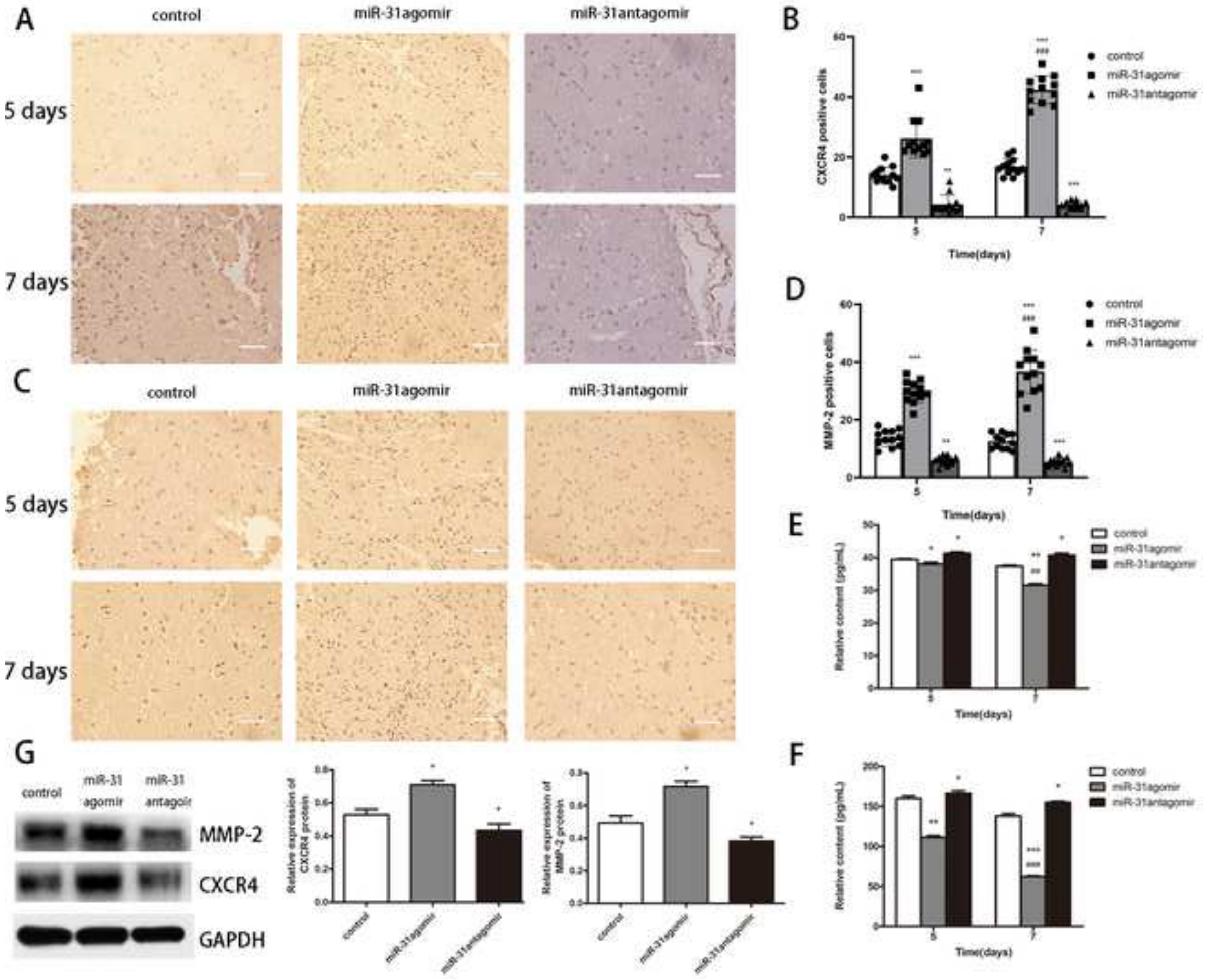

Supplement: Attachment — Submitted filename: Reviewer KE_PONE-D-21-40503.pdf [file pone.0272499.s003.pdf]
